# An Anionic Dinuclear Ruthenium Dihydrogen Complex of Relevance for Alkyne *gem*‐Hydrogenation

**DOI:** 10.1002/anie.202201311

**Published:** 2022-04-19

**Authors:** Tobias Biberger, Nils Nöthling, Markus Leutzsch, Christopher P. Gordon, Christophe Copéret, Alois Fürstner

**Affiliations:** ^1^ Max-Planck-Institut für Kohlenforschung 45470 Mülheim/Ruhr Germany; ^2^ Department of Chemistry and Applied Biosciences, ETH Zürich Vladimir-Prelog-Weg 1–5 8093 Zürich Switzerland

**Keywords:** Dihydrogen Complexes, *gem*-Hydrogenation, Metathesis, NMR Spectroscopy, Ruthenium

## Abstract

During an investigation into the fate of ruthenium precatalysts used for light‐driven alkyne *gem*‐hydrogenation reactions with formation of Grubbs‐type ruthenium catalysts, it was found that the reaction of [(IPr)(η^6^‐cymene)RuCl_2_] with H_2_ under UV‐irradiation affords an anionic dinuclear σ‐dihydrogen complex, which is thermally surprisingly robust. Not only are anionic σ‐complexes in general exceedingly rare, but the newly formed species seems to be the first example lacking any structural attributes able to counterbalance the negative charge and, in so doing, prevent oxidative insertion of the metal centers into the ligated H_2_ from occurring.

## Graphical abstract

Cationic and neutral non‐classical σ‐H_2_ complexes are plentiful, but anionic ones remain exceedingly rare. It was during an investigation into the fate of a Ru‐precatalyst in the light‐driven alkyne *gem*‐hydrogenation that such a species was encountered, which seems to be “first‐in‐class”: it lacks any substructures that counteract the negative charge and prevent oxidative insertion of the metal centers into the ligated H−H bond from occurring.

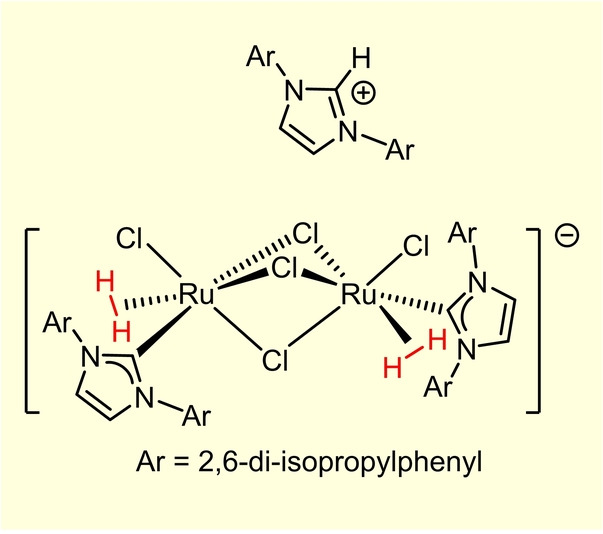

The ability to transfer both H‐atoms of H_2_ to one and the same C‐atom of an internal alkyne (“*gem*‐hydrogenation”) is a reactivity mode that was discovered only recently; it leads to the formation of a methylene group and concomitant generation of a discrete metal carbene at the adjacent position (Scheme 1).^[1]^ This transformation is arguably versatile as it allows potentially hazardous carbene sources such as diazo derivatives to be replaced by an appropriate acetylene derivative and H_2_. This conceptually novel entry into metal carbene complexes was initially observed with [Cp*Ru]‐based catalysts.^[2]^ The mechanism by which they operate is now fairly well understood,[^2^, ^3^, ^4^] and the ability to generate reactive piano stool ruthenium carbene intermediates by catalytic *gem*‐hydrogenation has already served the development of a host of new transformations.[^3^, ^4^, ^5^, ^6^, ^7^, ^8^, ^9^, ^10^]

**Scheme 1:**
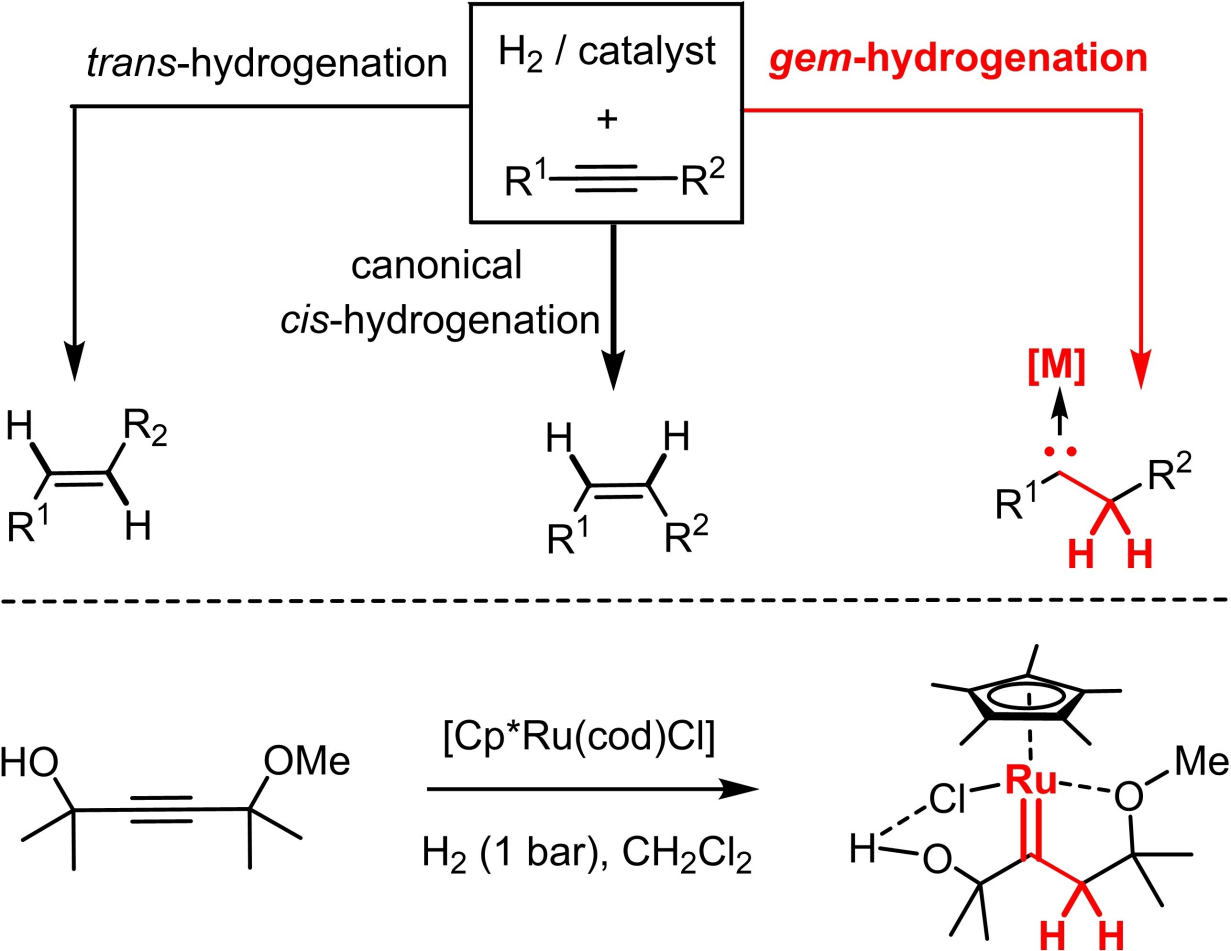
The concept of alkyne *gem*‐hydrogenation and the first fully characterized metal carbene complex made in this way;[^2^, ^3^] cod=1,5‐cyclooctadiene; Cp*=pentamethylcyclopentadienyl.

The currently only other confirmed case uses complexes of type [(NHC)(η^6^‐arene)MCl_2_] (**1**, M=Ru, Os; NHC=N‐heterocyclic carbene); strikingly, they mandate photochemical activation for reasons that are not entirely clear.^[11]^ This light‐driven system constitutes an orthogonal gateway to second generation Grubbs‐type catalysts for olefin metathesis and therefore merits close attention.^[12]^ Initially, however, the yield of complex **3** **a** was rather low, likely because of the clash between the ethyl substituent on the incipient carbene ligand and the N‐aryl group of the NHC (Scheme 2A).^[11]^ Therefore, an indirect route was pursued, in which the actual *gem*‐hydrogenation step affords the iodo‐chelate complex **4** in the first place, wherein the ruthenacyclic array is turned by 90 °C to prevent any such interligand repulsion; indeed, this reaction proved much more efficient (Scheme 2B).^[13]^ Subsequent cross metathesis of **4** with styrenes of type **5** leads to prototype Hoveyda‐Grubbs‐type catalysts **3** **b**, **c** in good overall yield.[^12^, ^14^]

**Scheme 2:**
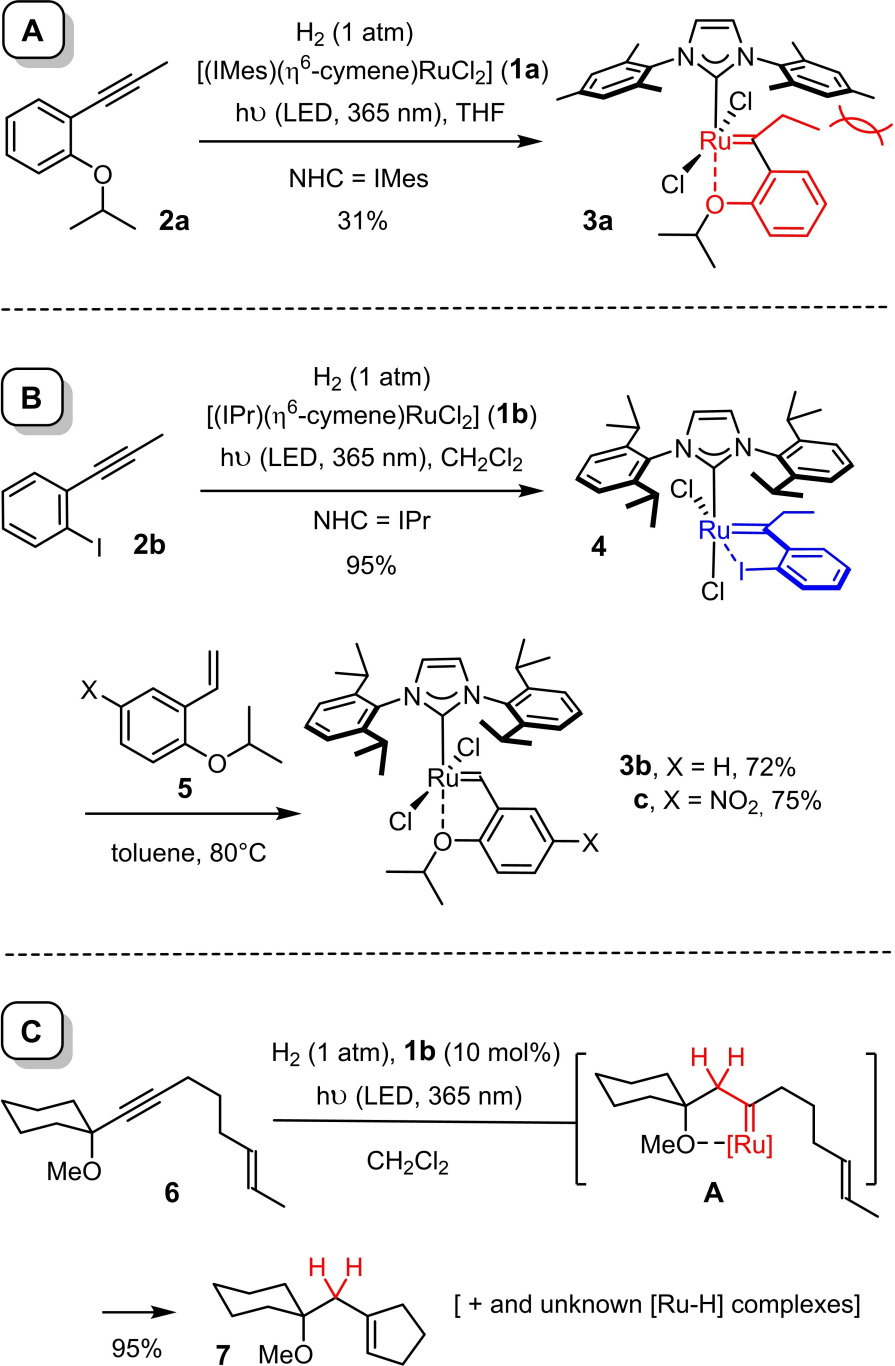
A) Initial preparation of a Hoveyda–Grubbs‐type complex by light‐driven *gem*‐hydrogentation; B) improved approach; C) prototype example of a catalytic hydrogenative metathesis; IMes=1,3‐bis(2,4,6‐trimethylphenyl)‐1,3‐dihydro‐2*H*‐imidazol‐2‐ylidene; IPr=1,3‐bis(2,6‐diisopropylphenyl)‐1,3‐dihydro‐2*H*‐imidazol‐2‐ylidene.

Despite this significant advance, it seemed equally relevant to investigate the fate of the ruthenium, because a better understanding for potentially competing processes might also help to improve the reaction. To this end, enyne **6** was subjected to hydrogenative metathesis to afford cyclopentene **7** in excellent yield (Scheme 2C). The ^1^H NMR spectrum of the crude material showed several signals in the high‐field region, indicating the concomitant formation of different ruthenium hydride species (see the Supporting Information). Various control experiments were carried out to identify at least the major constituents. In a first foray, a solution of **1** **b** was stirred in the absence of the enyne under H_2_ atmosphere in the dark to give a ruthenium hydride resonating at −6.06 ppm (Scheme 3). This sharp signal had also been present at the end of the catalytic reaction leading to **7**; it belongs to complex **8**, which was isolated in 42 % yield. If one considers that the formation of **8** releases one equivalent of HCl that will decompose the starting complex **1** **b** from which it derives, this yield actually indicates an almost quantitative transformation.^[15]^

**Scheme 3:**
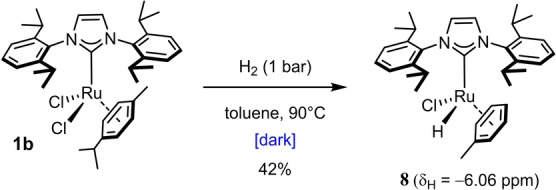
Hydrogenation of the precatalyst **1** **b** in the dark.

Figure 1 shows the structure of **8** in the solid state. It is of note that the cymene ligand of the starting material has been replaced by toluene used as the solvent.[^16^, ^17^] The hydride ligand was located on the difference Fourier map: with 1.51(2) Å, the Ru1−H1 distance falls into the typical range of ruthenium hydride complexes.^[18]^ Attempts to use isolated **8** as precatalyst for the *gem*‐hydrogenation of **6** (and related substrates) met with failure: although the formation of the corresponding *E*‐alkenes by *trans*‐hydrogenation of the triple bond could be confirmed by comparison with authentic samples,^[19]^ the mixtures proved too complex to be fully analyzed. We can hence conclude that complex **8** is generated in the dark as well as upon UV irradiation in the catalytic set‐up, but this pathway constitutes a dead end as far as *gem*‐hydrogenation is concerned.

**Figure 1:**
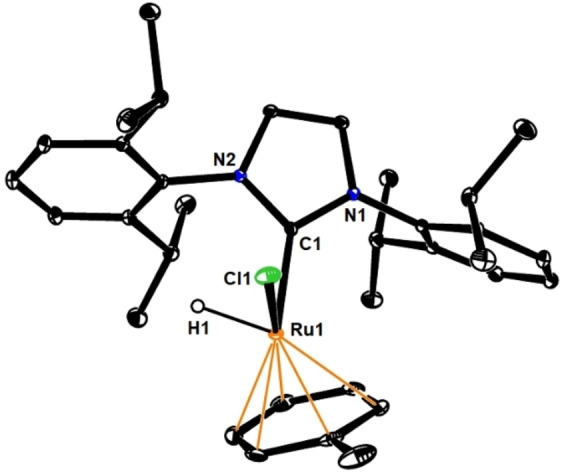
Structure of complex **8** in the solid state (pentane solute in the unit cell and all H‐atoms other than Ru−H omitted for clarity); selected bond lengths [Å]: Ru1−H1 1.51(2), Ru1−C1 2.0483(13), Ru1−Cl1 2.4018(4).

When the hydrogenation of **1** **b** was repeated under constant UV‐irradiation (LED, 365 nm), a different hydride species was obtained as the major product, which had basically been absent when the reaction was carried out in the dark (Scheme 4). This air‐ and moisture sensitive compound resonating at *δ*_H_ ([D_8_]‐THF)=−11.5 ppm (br s, 4*H*) could be isolated in analytically pure form but in low yield (22 %); importantly, it is this complex which corresponds to the major ruthenium hydride species in the crude material of the hydrogenative metathesis of enyne **6** with formation of **7** (Scheme 2C). The NMR spectra were too complex to allow for an unambiguous structure assignment. After considerable experimentation, however, we managed to grow crystals suitable for X‐ray diffraction from a solution in CH_2_Cl_2_/C_6_F_6_, which was gradually cooled from +20 °C to −50 °C over the course of two weeks.^[20]^ The structure of the complex in the solid state is truly striking (Figure 2):^[17]^ complex **9** is a salt comprised of an anionic diruthenium bis‐dihydrogen entity escorted by the imidazolium cation [IPr−H]^+^. This net composition implies that the NHC ligand of an additional equivalent of **1** **b** must have been protonated off by HCl generated in situ in order to generate the observed cation and deliver the additional chloride contained in the ruthenate unit. Therefore, addition of [IPr−H]Cl at the outset should improve the result; indeed, **9** was isolated in 73 % yield when the hydrogenation was performed in the presence of this additive under otherwise identical conditions. With now ample material in hand, a control experiment could be carried out which showed that **9** is capable of catalyzing the light‐driven *gem*‐hydrogenation of **6** with formation of cyclopentene **7**, even though the reaction rate is substantially slower than that observed with parent **1** **b** (see the Supporting Information). Therefore complex **9** cannot be an on‐cycle intermediate but likely represents an off‐cycle “reservoir”, from which the actual (probably mononuclear) catalyst can be released in situ.[^21^, ^22^]

**Scheme 4:**
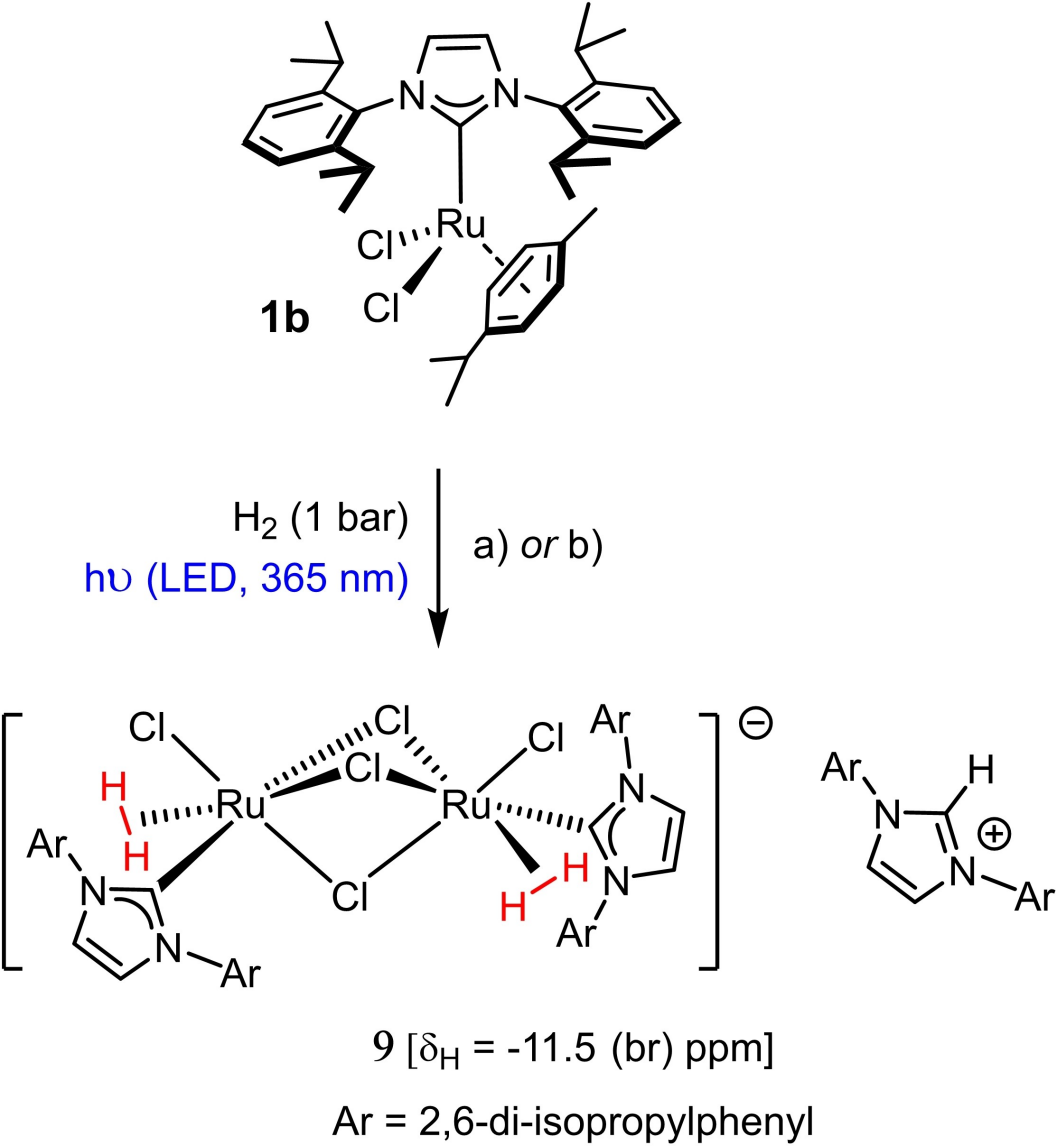
a) H_2_ (1 bar), UV (365 nm, LED), toluene, RT, 22 %; b) [IPr‐H]Cl (1 equiv), H_2_ (1 bar), UV (365 nm, LED), toluene, RT, 73 %.

**Figure 2:**
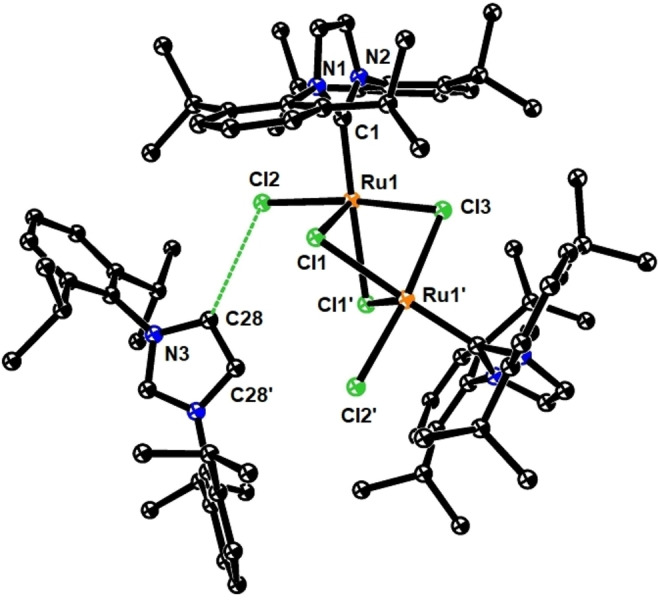
Structure of complex **9** in the solid state; solute solvent molecules, disorder of two isopropyl groups, and all H‐atoms omitted for clarity (for the full structure, see the Supporting Information); selected bond lengths [Å] and angles [°]: Ru1−C1 2.004(3), Ru1−Cl1 2.4288(7), Ru1−Cl2 2.3941(7), Ru1−Cl3 2.4118(7), Cl2⋅⋅⋅C28 3.39, Ru1⋅⋅⋅Ru1′ 3.27(1), C1−Ru1−Cl1′ 177.0, Cl1−Ru1−Cl2 92.6, Cl1−Ru1−Cl3 82.6, Cl2−Ru1−Cl3 172.9.

What makes complex **9** truly special, however, is its unprecedented composition and structure. The X‐ray data (Figure 2)^[17]^ show that the anion consists of a [Ru_2_Cl_5_]^−^ entity held together by three μ‐bridging chlorides; terminal chlorides on each metal center in *cis*‐arrangement relative to the Ru⋅⋅⋅Ru vector and two NHC ligands complete the bimetallic core. Each Ru atom adopts a distorted octahedral coordination geometry: although the X‐ray data did not allow the bound H_2_ to be located, high residual electron density of 3.18 e Å^−3^ in the region of the yet remaining seemingly vacant site can be taken as indirect evidence (see the Supporting Information). No less striking is the close contact between the ruthenate and its cationic companion, as manifested in short intermolecular contacts (≈3.39 Å) between the terminal chlorides (Cl2/Cl2′) and the olefinic sites of the imidazolium ring (C28/C28′). The proximity is retained in [D_8_]‐THF solution, as can be deduced from the strong intermolecular NOE signals between the ions of this salt (see the Supporting Information).

Although the presence of bound H_2_ could not be confirmed by crystallographic means, the ^1^H NMR spectra are unequivocal. Moreover, NMR allows insights into the actual bonding mode to be gained. To this end, the HD isotopomer [D_2_]‐**9** was prepared, which features a triplet of 1 : 1 : 1 intensity in lieu of the broad singlet of unlabeled **9** in the hydride region of the spectrum (Figure 3).^[23]^ In‐depth studies by the groups of Morris^[24]^ and Heinekey^[25]^ had shown that the coupling constant ^1^ *J*_HD_ is innately correlated with the distance between the bound H‐atoms. In the present case, the recorded ^1^ *J*_HD_=29.4 Hz indicates a *d*_H,H_=0.93 Å (Morris) or 0.95 Å (Heinekey). These calculated values well below 1 Å imply that the H−H σ‐bond is intact and **9** must therefore be classified as a true dihydrogen complex (at each of the two Ru centers) rather than a classical dihydride species.[^26^, ^27^, ^28^, ^29^, ^30^, ^31^, ^32^, ^33^, ^34^] The formal oxidation state of the two identical Ru‐centers is hence +2 each.

**Figure 3:**
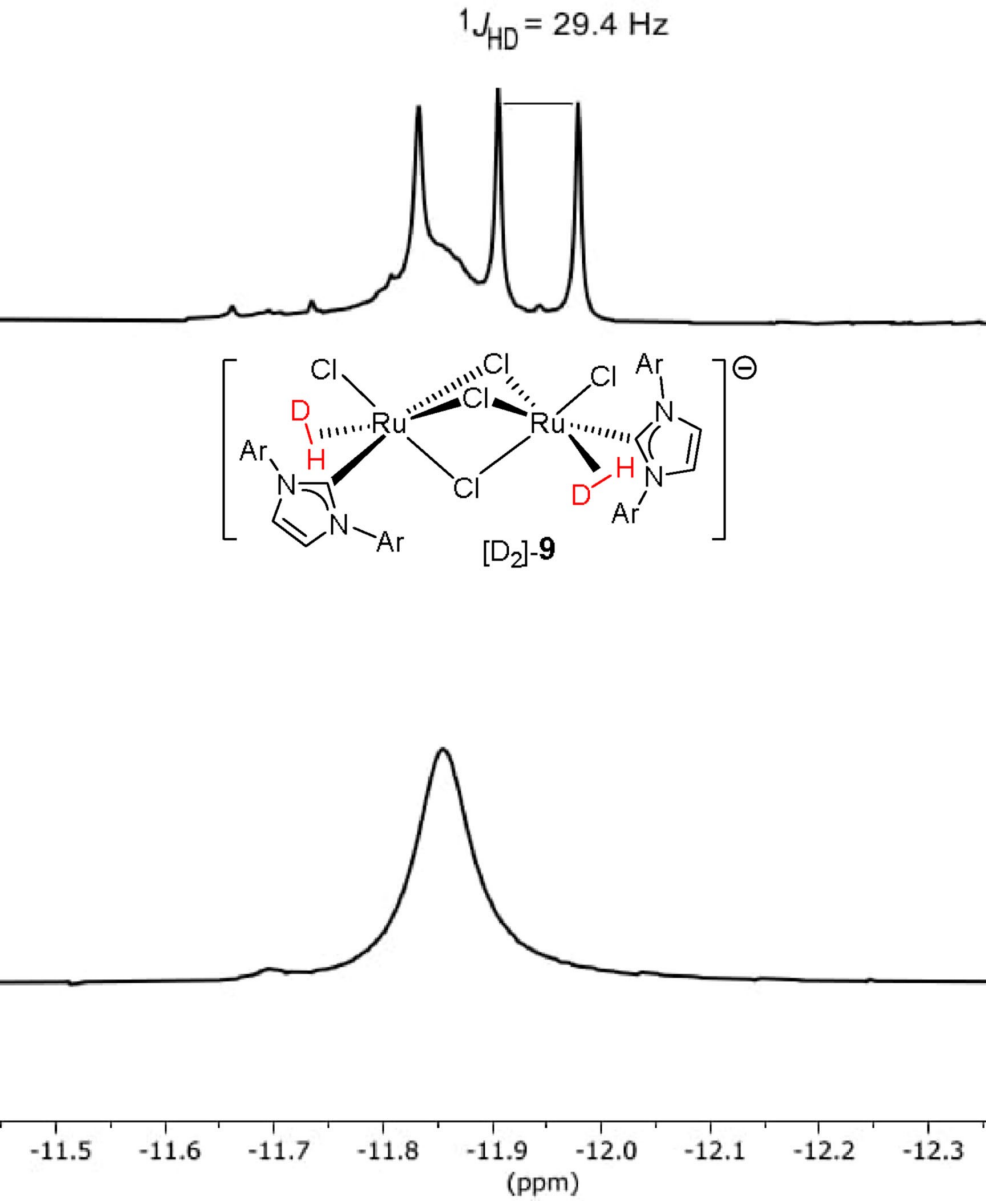
Hydride region of the ^1^H NMR (CD_2_Cl_2_) spectrum of **9** (bottom) and the corresponding isotopomer [D]_2_‐**9** (top), in which both H_2_ ligands are replaced by (H−D).

This conclusion is perplexing in view of the fact that the dimetallic unit is negatively charged, which increases the net electron density at [Ru] and should hence facilitate cleavage of the H−H bond by oxidative insertion.^[35]^ Therefore, additional evidence was sought to confirm that **9** is indeed a genuine homobimetallic bis‐σ‐dihydrogen complex. To this end, the temperature dependence of the minimum spin‐lattice relaxation time *T*_1_(min) was determined, which has been shown to be another probe for the bonding situation in hydrogen complexes.^[36]^ The plot of ln(*T*_1_) against T^−1^ features flat parabolic curves rather than sharp V‐shaped minima (Figure 4); this feature suggests that rotation of the H_2_ ligand is close to the Larmor frequency of the NMR spectrometers.^[37]^ With this boundary condition in mind, the recorded *T*_1_(min)=11.8 ms (300 MHz) can be correlated to a H−H distance of 0.96 Å,^[38]^ which is in excellent agreement with the data derived from the ^1^ *J*_HD_ experiment. Moreover, it fits very well to previous observations in the literature that H_2_ ligands *trans* to chloride in bridging ruthenium dihydrogen complexes have slightly elongated H−H distances and rotate in an “intermediate motion regime”.^[38]^

**Figure 4:**
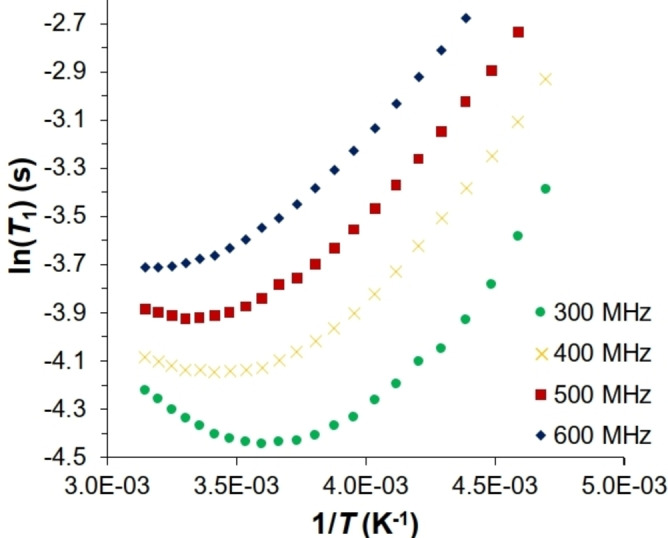
Plot of ln(*T*_1_) against T^−1^ for different NMR spectrometer frequencies.

Finally, DFT calculations at the PBE0/Def2‐SVP/SDD level of theory provided additional support. Under the premise that H_2_ is bound to each Ru center, geometry optimization reproduced the crystal structure of complex **9** very well and resulted in a computed H−H distance of 0.93 Å (Figure 5). The notion that the bound H_2_ forms a σ‐complex is nicely visualized by the calculated electron density map, which shows significant electron density in the H−H rather than the Ru−H vectors, which speaks for a largely intact σ‐bond.

**Figure 5:**
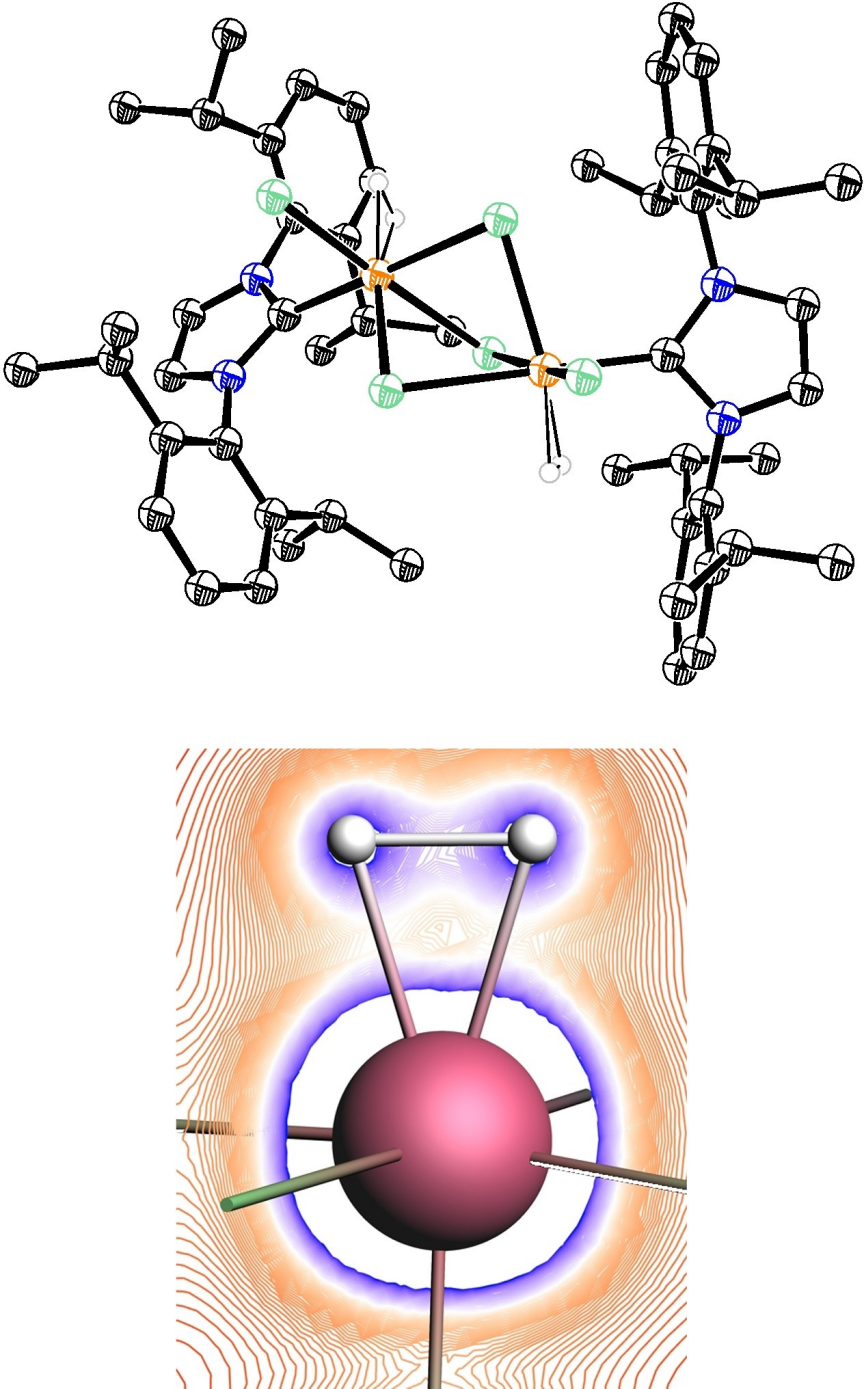
Top: Computed structure of **9** comprising σ‐bound H_2_ ligands at each Ru center; bottom: plot of the computed electron density in the Ru/H_2_ plane; blue: high e‐density, red: low e‐density (dark blue: 1.0 a.u., white: 0.5 a.u., red: <0.5 a.u).

In the aftermath of Kubas’ groundbreaking discovery,[^39^, ^40^] innumerous H_2_ complexes were characterized in great detail. The observed bonding modes represent a continuum between truly “nonclassical” complexes with an intact σ‐H−H bond (*d*_H,H_≈0.8–1.0 Å) and typical dihydride species (*d*_H,H_≥1.6 Å).[^26^, ^27^, ^28^, ^29^, ^30^, ^31^, ^32^, ^33^, ^34^] As expected, low electron density at the central metal helps to prevent oxidative insertion from occurring: many true σ‐complexes are therefore cationic entities, or, if neutral, often carry strong π‐acceptor ligands. A high oxidation state is also clearly helpful, and first row transition metals are more likely to form σ‐complexes than their higher homologues. Conversely, anionic σ‐complexes were unknown until very recently and continue to be exceedingly rare (Figure 6): except for **10**,^[41]^ which has only been observed in an Ar‐matrix, the few well‐characterized examples (**11**–**13**) are distinguished by the presence of Lewis‐acidic metallo‐ligands that exert an “inverse *trans*‐influence” and hence deprive the anionic center of net charge density.[^42^, ^43^, ^44^] None of these structural and electronic criteria applies to **9**, which is therefore unique at this point. It is the anionic charge that also sets **9** apart from otherwise close relatives such as **14**–**17**, which comprise similar diruthenium cores with three μ‐bridging anionic ligands, but all of which are neutral entities (Figure 7).[^45^, ^46^, ^47^, ^48^, ^49^, ^50^, ^51^, ^52^, ^53^, ^54^]

**Figure 6:**
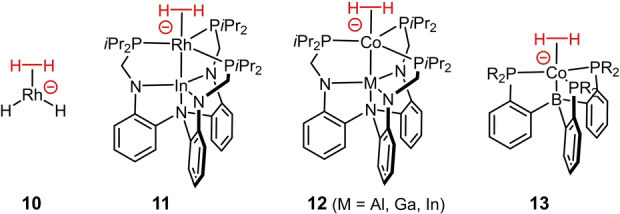
Known anionic σ‐H_2_ complexes.

**Figure 7:**
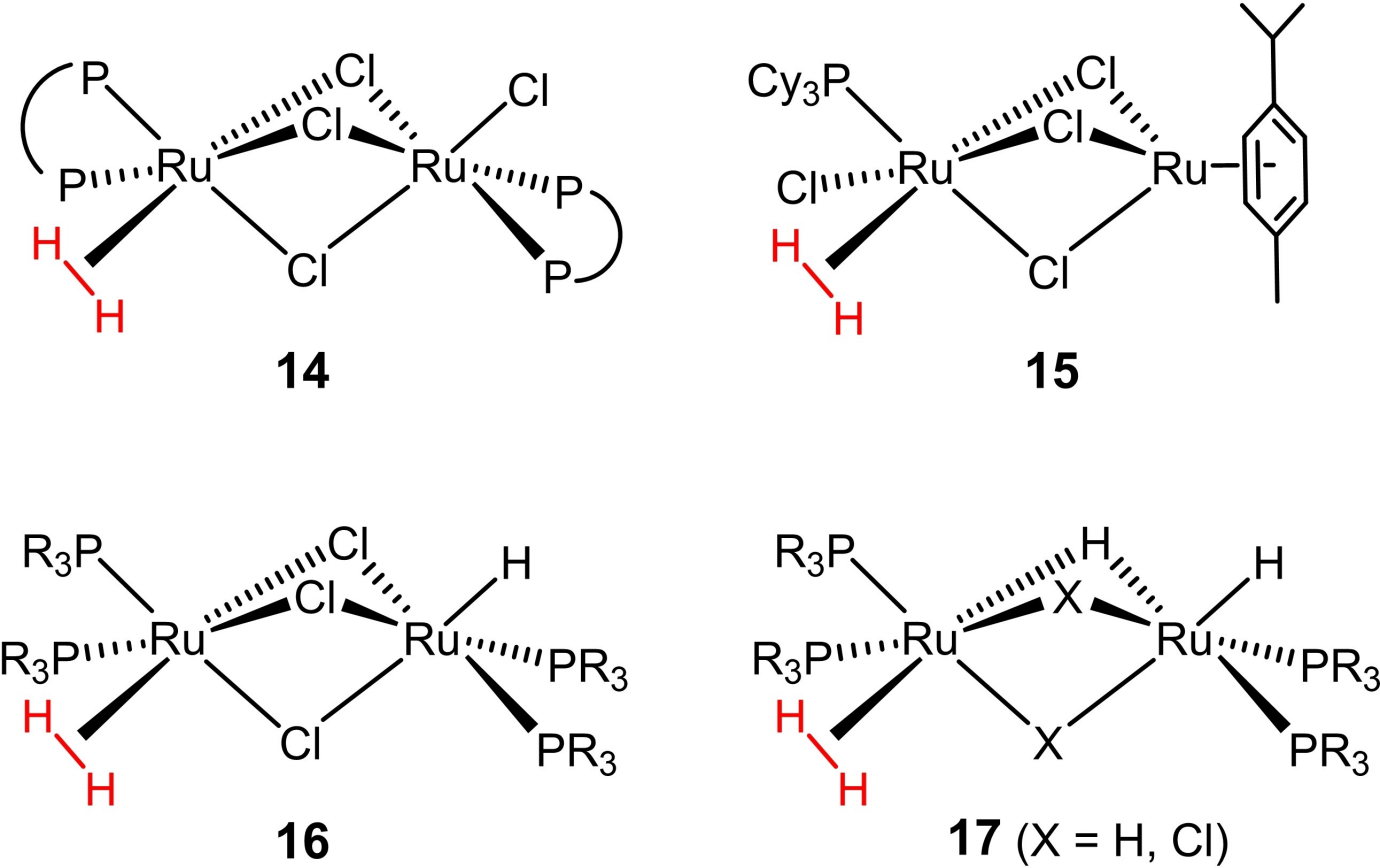
Neutral relatives of complex **9**.

With the foregoing in mind, the structure of **9** was re‐inspected in an attempt to explain why this complex is formed so easily and found thermally quite stable (under inert conditions). With 169.6°, the angle between the *trans*‐disposed chloride ligand and the bound H_2_ (at the computed site) is far from linear (Figure 5). This arrangement reflects the distorted octahedral coordination geometry about the central metal; it infringes on p_π_→d_π_ electron donation, which in turn translates into reduced electron back‐donation from the metal into the σ*‐orbital of the H_2_‐ligand. An arguably even more conspicuous feature is the Ru1−C1 distance: with only 2.004(3) Å, it is the shortest [Ru‐(IPr)] bond known to date (the average of the 84 symmetry‐independent bond lengths of 52 different complexes is 2.098 Å; the Ru1−C1 distance in **8** is 2.0483(13) Å; for details, see the Supporting Information). It is hence the NHC ligand, which seems to exert non‐negligible π‐acceptor properties and helps to counterbalance the negative charge delocalized over the ruthenate entity. Although certainly not unprecedented, the present case clearly illustrates this perhaps still underappreciated chemical virtue of this ligand class.[^55^, ^56^, ^57^, ^58^]

In summary, two different decomposition reactions of [(IPr)(η^6^‐cymene)RuCl_2_] have been unraveled, which concur and/or compete with the formation of Grubbs‐type carbenes by light‐driven *gem*‐hydrogenation of internal alkynes. Whereas the classical hydride complex **8** marks a dead end, the dinuclear non‐classical σ‐H_2_ complex **9** retains catalytic activity, even though it is likely an off‐cycle reservoir. All available spectroscopic and computational evidence suggests that the σ‐bond of the H_2_ ligands is intact, despite the net anionic charge and the lack of prototypical π‐acceptor ancillary ligands; actually, it is the N‐heterocyclic carbene that seems to exert this role. Therefore, **9** is clearly distinguished from the very few other anionic dihydrogen complexes known to date and represents the “first‐in‐class” of a new type, which may have been deemed elusive in the past.

## Conflict of interest

The authors declare no conflict of interest.

## Supporting information

As a service to our authors and readers, this journal provides supporting information supplied by the authors. Such materials are peer reviewed and may be re‐organized for online delivery, but are not copy‐edited or typeset. Technical support issues arising from supporting information (other than missing files) should be addressed to the authors.

Supporting Information

Supporting Information

## Data Availability

The data that support the findings of this study are available in the Supporting Information of this article.
