# Supplementary material for: An Anionic Dinuclear Ruthenium Dihydrogen Complex of Relevance for Alkyne gem‐Hydrogenation
Source: Angew Chem Int Ed Engl. 2022 Apr 19;61(24):e202201311. doi: 10.1002/anie.202201311 (PMC9322539; doi:10.1002/anie.202201311)
Supplement: Supplementary file 2 — Supporting Information [file ANIE-61-0-s002.pdf]

Angewandte Chemie
Eine Zeitschrift der Gesellschaft Deutscher Chemiker

Supporting Information

An Anionic Dinuclear Ruthenium Dihydrogen Complex of Relevance for Alkyne *gem*-Hydrogenation

*T. Biberger, N. Nöthling, M. Leutzsch, C. P. Gordon, C. Copéret, A. Fürstner**

Supporting Crystallographic Data

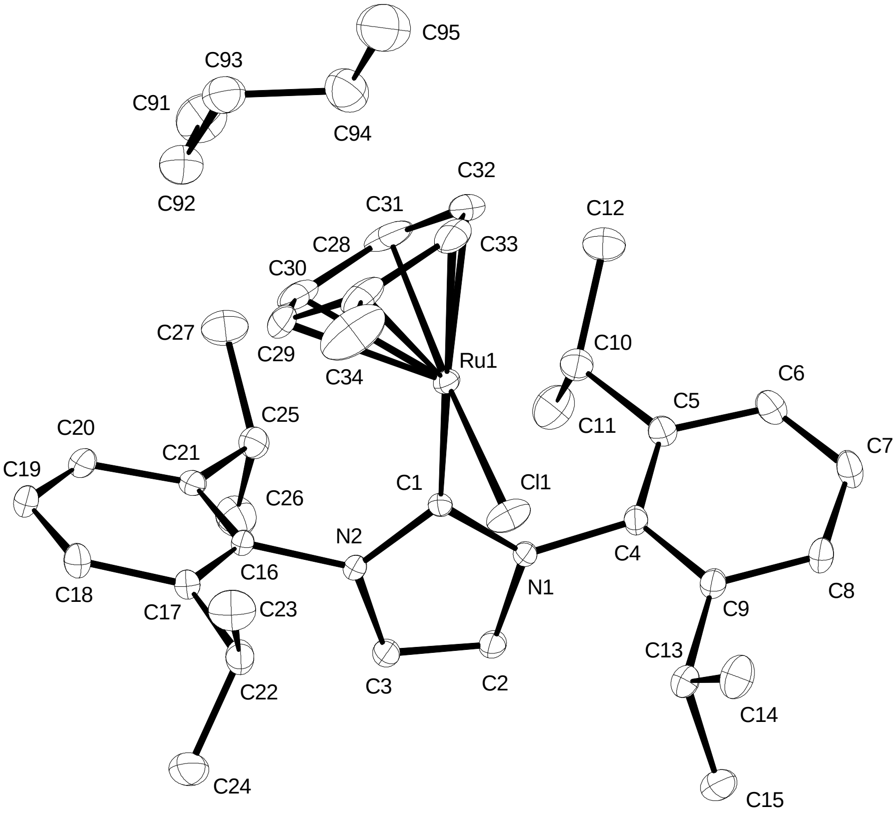

Figure S1. The molecular structure of complex **8·(pentane)** in the solid state; H-atoms were removed for clarity.

X-ray Crystal Structure Analysis of Complex 8·(pentane): $C_{39}H_{57}ClN_2Ru$, M_r = 690.38 g mol^{-1}, yellow plate, crystal size 0.100 x 0.048 x 0.042 mm^3, monoclinic, space group $P2_1/n$ [14], a = 13.1675(9) Å, b = 12.1818(8) Å, c = 22.5540(15) Å, β = 100.054(3)°, V = 3562.2(4) Å^3, T = 100(2) K, Z = 4, D_{calc} = 1.287 g·cm^3, λ = 0.71073 Å, $\mu(Mo\text{-}K_\alpha)$ = 0.544 mm^{-1}, Gaussian absorption correction (T_{min} = 0.96165, T_{max} = 0.98642), Bruker-AXS Kappa Mach3 with APEX-II detector and IµS microfocus source, 1.675 < θ < 31.744°, 117341 measured reflections, 11966 independent reflections, 10447 reflections with $I > 2\sigma(I)$, R_{int} = 0.0357. The structure was solved by *SHELXT* and refined by full-matrix least-squares (*SHELXL*) against F^2 to R_1 = 0.0330 [$I > 2\sigma(I)$], wR_2 = 0.0708, 403 parameters, 0 restraints. **CCDC-2143698**

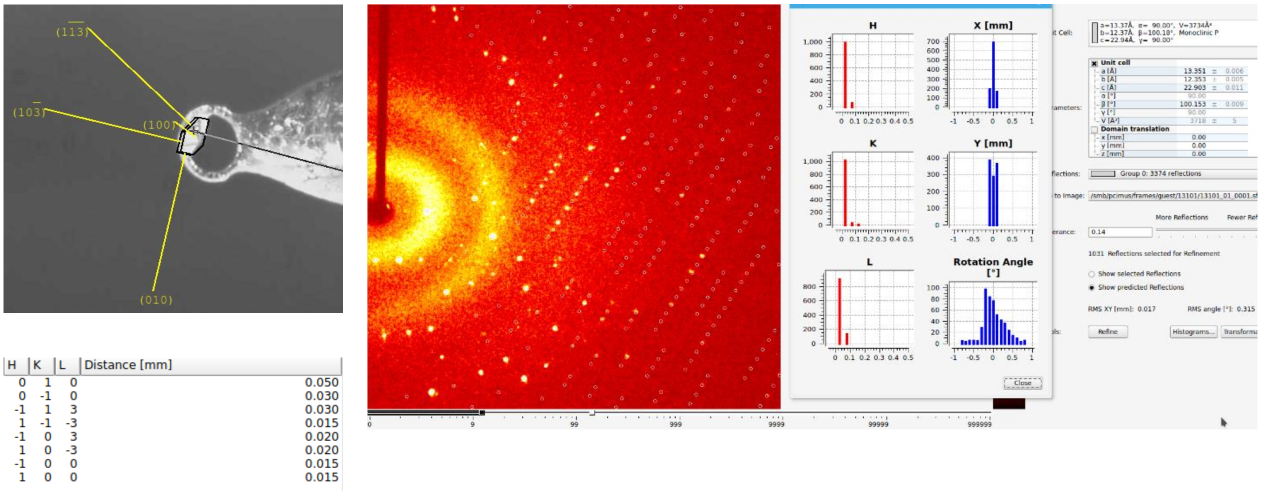

Figure S2. Crystal faces and unit cell determination/refinement of **8·(pentane)**

INTENSITY STATISTICS FOR DATASET

Resolution	#Data	#Theory	%Complete	Redundancy	Mean I	Mean I/s	Rmerge	Rsigma
Inf - 2.88	187	187	100.0	15.40	94.37	111.67	0.0177	0.0070
2.88 - 1.88	441	441	100.0	16.95	52.38	100.13	0.0192	0.0072
1.88 - 1.48	630	630	100.0	17.11	37.22	86.94	0.0213	0.0080
1.48 - 1.29	627	627	100.0	17.17	22.71	71.45	0.0271	0.0097
1.29 - 1.17	616	616	100.0	16.72	19.39	64.29	0.0329	0.0111
1.17 - 1.08	665	665	100.0	15.17	16.67	54.70	0.0378	0.0132
1.08 - 1.01	684	684	100.0	11.65	16.83	45.10	0.0383	0.0160
1.01 - 0.96	633	633	100.0	9.95	14.66	36.86	0.0435	0.0195
0.96 - 0.92	595	595	100.0	8.71	10.52	27.92	0.0546	0.0264
0.92 - 0.88	694	694	100.0	7.91	10.17	25.69	0.0566	0.0289
0.88 - 0.85	632	632	100.0	7.56	8.80	22.94	0.0631	0.0339
0.85 - 0.83	461	461	100.0	7.32	7.98	20.87	0.0684	0.0377
0.83 - 0.80	792	792	100.0	7.08	7.40	18.30	0.0756	0.0417
0.80 - 0.78	574	574	100.0	6.79	6.92	17.02	0.0822	0.0465
0.78 - 0.76	679	679	100.0	6.59	6.71	16.01	0.0871	0.0498
0.76 - 0.74	737	737	100.0	6.49	6.35	14.71	0.0926	0.0545
0.74 - 0.73	374	374	100.0	6.22	6.63	14.75	0.0961	0.0548
0.73 - 0.71	863	863	100.0	6.14	5.72	12.78	0.1056	0.0639
0.71 - 0.70	482	482	100.0	5.87	5.41	11.81	0.1092	0.0705
0.70 - 0.68	1110	1244	89.2	4.40	5.10	9.90	0.1198	0.0890
0.78 - 0.68	4245	4379	96.9	5.75	5.87	12.94	0.1013	0.0650
Inf - 0.68	12476	12610	98.9	9.52	14.39	34.67	0.0353	0.0223

Table S1. Crystal Data and Structure Refinement.

Identification code	**8·(pentane)** (internal: 13101)	
Empirical formula	$C_{39} H_{57} Cl N_2 Ru$	
Color	yellow	
Formula weight	690.38 g·mol^{-1}	
Temperature	100(2) K	
Wavelength	0.71073 Å	
Crystal system	MONOCLINIC	
Space group	$P2_1$/n, (no. 14)	
Unit cell dimensions	a = 13.1675(9) Å	α= 90°
	b = 12.1818(8) Å	β= 100.054(3)°
	c = 22.5540(15) Å	γ = 90°
Volume	3562.2(4) Å^3	
Z	4	
Density (calculated)	1.287 Mg · m^{-3}	
Absorption coefficient	0.544 mm^{-1}	
F(000)	1464 e	
Crystal size	0.100 x 0.048 x 0.042 mm^3	
θ range for data collection	1.675 to 31.744°.	
Index ranges	-19 ≤ h ≤ 19, -17≤ k ≤ 17, -33 ≤ l ≤ 32	
Reflections collected	117341	
Independent reflections	11966 [R_{int} = 0.0357]	
Reflections with I>2σ(I)	10447	
Completeness to θ = 25.242°	100.0 %	
Absorption correction	Gaussian	
Max. and min. transmission	0.99 and 0.96	
Refinement method	Full-matrix least-squares on F^2	
Data / restraints / parameters	11966 / 0 / 403	
Goodness-of-fit on F^2	1.119	
Final R indices [I>2σ(I)]	R_1 = 0.0330	wR^2 = 0.0708
R indices (all data)	R_1 = 0.0415	wR^2 = 0.0736
Largest diff. peak and hole	0.8 and -0.7 e · Å^{-3}	

Table S2. Complex 8·(pentane): Bond lengths [Å] and Angles [°].

Ru(1)-H	1.51(2)	Ru(1)-Cl(1)	2.4018(4)
Ru(1)-C(1)	2.0483(13)	Ru(1)-C(28)	2.2809(16)
Ru(1)-C(29)	2.3191(15)	Ru(1)-C(30)	2.2168(16)
Ru(1)-C(31)	2.1829(17)	Ru(1)-C(32)	2.1789(17)
Ru(1)-C(33)	2.2417(16)	N(1)-C(1)	1.3730(17)
N(1)-C(2)	1.3916(17)	N(1)-C(4)	1.4433(18)
N(2)-C(1)	1.3731(17)	N(2)-C(3)	1.3900(18)
N(2)-C(16)	1.4407(18)	C(2)-H(2)	0.9500
C(2)-C(3)	1.343(2)	C(3)-H(3)	0.9500
C(4)-C(5)	1.410(2)	C(4)-C(9)	1.398(2)
C(5)-C(6)	1.393(2)	C(5)-C(10)	1.519(2)
C(6)-H(6)	0.9500	C(6)-C(7)	1.391(2)
C(7)-H(7)	0.9500	C(7)-C(8)	1.379(2)
C(8)-H(8)	0.9500	C(8)-C(9)	1.402(2)
C(9)-C(13)	1.517(2)	C(10)-H(10)	1.0000
C(10)-C(11)	1.528(2)	C(10)-C(12)	1.535(2)
C(11)-H(11A)	0.9800	C(11)-H(11B)	0.9800
C(11)-H(11C)	0.9800	C(12)-H(12A)	0.9800
C(12)-H(12B)	0.9800	C(12)-H(12C)	0.9800
C(13)-H(13)	1.0000	C(13)-C(14)	1.535(2)
C(13)-C(15)	1.538(2)	C(14)-H(14A)	0.9800
C(14)-H(14B)	0.9800	C(14)-H(14C)	0.9800
C(15)-H(15A)	0.9800	C(15)-H(15B)	0.9800
C(15)-H(15C)	0.9800	C(16)-C(17)	1.396(2)
C(16)-C(21)	1.407(2)	C(17)-C(18)	1.402(2)
C(17)-C(22)	1.521(2)	C(18)-H(18)	0.9500
C(18)-C(19)	1.380(2)	C(19)-H(19)	0.9500
C(19)-C(20)	1.390(2)	C(20)-H(20)	0.9500
C(20)-C(21)	1.398(2)	C(21)-C(25)	1.520(2)
C(22)-H(22)	1.0000	C(22)-C(23)	1.527(2)
C(22)-C(24)	1.531(2)	C(23)-H(23A)	0.9800
C(23)-H(23B)	0.9800	C(23)-H(23C)	0.9800

C(24)-H(24A)	0.9800	C(24)-H(24B)	0.9800
C(24)-H(24C)	0.9800	C(25)-H(25)	1.0000
C(25)-C(26)	1.531(2)	C(25)-C(27)	1.531(2)
C(26)-H(26A)	0.9800	C(26)-H(26B)	0.9800
C(26)-H(26C)	0.9800	C(27)-H(27A)	0.9800
C(27)-H(27B)	0.9800	C(27)-H(27C)	0.9800
C(28)-C(29)	1.406(2)	C(28)-C(33)	1.410(3)
C(28)-C(34)	1.507(3)	C(29)-H(29)	0.9500
C(29)-C(30)	1.401(3)	C(30)-H(30)	0.9500
C(30)-C(31)	1.413(3)	C(31)-H(31)	0.9500
C(31)-C(32)	1.424(3)	C(32)-H(32)	0.9500
C(32)-C(33)	1.397(3)	C(33)-H(33)	0.9500
C(34)-H(34A)	0.9800	C(34)-H(34B)	0.9800
C(34)-H(34C)	0.9800	C(91)-H(91A)	0.9800
C(91)-H(91B)	0.9800	C(91)-H(91C)	0.9800
C(91)-C(92)	1.508(3)	C(92)-H(92A)	0.9900
C(92)-H(92B)	0.9900	C(92)-C(93)	1.528(3)
C(93)-H(93A)	0.9900	C(93)-H(93B)	0.9900
C(93)-C(94)	1.530(3)	C(94)-H(94A)	0.9900
C(94)-H(94B)	0.9900	C(94)-C(95)	1.528(3)
C(95)-H(95A)	0.9800	C(95)-H(95B)	0.9800
C(95)-H(95C)	0.9800		

Cl(1)-Ru(1)-H	84.6(9)	C(1)-Ru(1)-H	77.7(8)
C(1)-Ru(1)-Cl(1)	84.81(4)	C(1)-Ru(1)-C(28)	146.12(6)
C(1)-Ru(1)-C(29)	115.51(6)	C(1)-Ru(1)-C(30)	99.52(6)
C(1)-Ru(1)-C(31)	108.19(7)	C(1)-Ru(1)-C(32)	138.27(8)
C(1)-Ru(1)-C(33)	175.00(7)	C(28)-Ru(1)-H	135.0(8)
C(28)-Ru(1)-Cl(1)	89.45(5)	C(28)-Ru(1)-C(29)	35.58(6)
C(29)-Ru(1)-H	162.7(8)	C(29)-Ru(1)-Cl(1)	106.91(5)
C(30)-Ru(1)-H	135.0(9)	C(30)-Ru(1)-Cl(1)	140.29(5)
C(30)-Ru(1)-C(28)	65.11(6)	C(30)-Ru(1)-C(29)	35.90(7)
C(30)-Ru(1)-C(33)	77.80(6)	C(31)-Ru(1)-H	100.0(8)
C(31)-Ru(1)-Cl(1)	166.83(5)	C(31)-Ru(1)-C(28)	78.55(7)
C(31)-Ru(1)-C(29)	66.03(7)	C(31)-Ru(1)-C(30)	37.46(7)
C(31)-Ru(1)-C(33)	67.11(7)	C(32)-Ru(1)-H	85.0(8)

C(32)-Ru(1)-Cl(1)	131.30(6)	C(32)-Ru(1)-C(28)	66.15(8)
C(32)-Ru(1)-C(29)	77.70(6)	C(32)-Ru(1)-C(30)	67.35(7)
C(32)-Ru(1)-C(31)	38.12(8)	C(32)-Ru(1)-C(33)	36.81(8)
C(33)-Ru(1)-H	101.1(8)	C(33)-Ru(1)-Cl(1)	99.96(5)
C(33)-Ru(1)-C(28)	36.32(7)	C(33)-Ru(1)-C(29)	64.68(6)
C(1)-N(1)-C(2)	111.81(11)	C(1)-N(1)-C(4)	128.51(11)
C(2)-N(1)-C(4)	119.59(11)	C(1)-N(2)-C(3)	111.89(11)
C(1)-N(2)-C(16)	126.82(11)	C(3)-N(2)-C(16)	120.40(11)
N(1)-C(1)-Ru(1)	127.39(10)	N(2)-C(1)-Ru(1)	129.44(10)
N(2)-C(1)-N(1)	102.76(11)	N(1)-C(2)-H(2)	126.6
C(3)-C(2)-N(1)	106.77(12)	C(3)-C(2)-H(2)	126.6
N(2)-C(3)-H(3)	126.6	C(2)-C(3)-N(2)	106.76(12)
C(2)-C(3)-H(3)	126.6	C(5)-C(4)-N(1)	118.32(13)
C(9)-C(4)-N(1)	118.42(13)	C(9)-C(4)-C(5)	122.95(13)
C(4)-C(5)-C(10)	121.55(13)	C(6)-C(5)-C(4)	117.22(14)
C(6)-C(5)-C(10)	121.23(14)	C(5)-C(6)-H(6)	119.4
C(7)-C(6)-C(5)	121.16(15)	C(7)-C(6)-H(6)	119.4
C(6)-C(7)-H(7)	119.9	C(8)-C(7)-C(6)	120.16(15)
C(8)-C(7)-H(7)	119.9	C(7)-C(8)-H(8)	119.3
C(7)-C(8)-C(9)	121.40(15)	C(9)-C(8)-H(8)	119.3
C(4)-C(9)-C(8)	117.10(14)	C(4)-C(9)-C(13)	123.18(13)
C(8)-C(9)-C(13)	119.69(13)	C(5)-C(10)-H(10)	107.2
C(5)-C(10)-C(11)	111.30(13)	C(5)-C(10)-C(12)	112.61(13)
C(11)-C(10)-H(10)	107.2	C(11)-C(10)-C(12)	110.93(15)
C(12)-C(10)-H(10)	107.2	C(10)-C(11)-H(11A)	109.5
C(10)-C(11)-H(11B)	109.5	C(10)-C(11)-H(11C)	109.5
H(11A)-C(11)-H(11B)	109.5	H(11A)-C(11)-H(11C)	109.5
H(11B)-C(11)-H(11C)	109.5	C(10)-C(12)-H(12A)	109.5
C(10)-C(12)-H(12B)	109.5	C(10)-C(12)-H(12C)	109.5
H(12A)-C(12)-H(12B)	109.5	H(12A)-C(12)-H(12C)	109.5
H(12B)-C(12)-H(12C)	109.5	C(9)-C(13)-H(13)	108.1
C(9)-C(13)-C(14)	111.85(13)	C(9)-C(13)-C(15)	110.43(13)
C(14)-C(13)-H(13)	108.1	C(14)-C(13)-C(15)	110.25(13)
C(15)-C(13)-H(13)	108.1	C(13)-C(14)-H(14A)	109.5
C(13)-C(14)-H(14B)	109.5	C(13)-C(14)-H(14C)	109.5

H(14A)-C(14)-H(14B)	109.5	H(14A)-C(14)-H(14C)	109.5
H(14B)-C(14)-H(14C)	109.5	C(13)-C(15)-H(15A)	109.5
C(13)-C(15)-H(15B)	109.5	C(13)-C(15)-H(15C)	109.5
H(15A)-C(15)-H(15B)	109.5	H(15A)-C(15)-H(15C)	109.5
H(15B)-C(15)-H(15C)	109.5	C(17)-C(16)-N(2)	119.58(13)
C(17)-C(16)-C(21)	123.06(13)	C(21)-C(16)-N(2)	117.19(13)
C(16)-C(17)-C(18)	117.05(14)	C(16)-C(17)-C(22)	122.80(13)
C(18)-C(17)-C(22)	120.10(14)	C(17)-C(18)-H(18)	119.4
C(19)-C(18)-C(17)	121.23(15)	C(19)-C(18)-H(18)	119.4
C(18)-C(19)-H(19)	119.7	C(18)-C(19)-C(20)	120.58(14)
C(20)-C(19)-H(19)	119.7	C(19)-C(20)-H(20)	119.7
C(19)-C(20)-C(21)	120.55(15)	C(21)-C(20)-H(20)	119.7
C(16)-C(21)-C(25)	121.97(13)	C(20)-C(21)-C(16)	117.48(14)
C(20)-C(21)-C(25)	120.53(14)	C(17)-C(22)-H(22)	108.1
C(17)-C(22)-C(23)	111.84(13)	C(17)-C(22)-C(24)	109.77(13)
C(23)-C(22)-H(22)	108.1	C(23)-C(22)-C(24)	110.67(15)
C(24)-C(22)-H(22)	108.1	C(22)-C(23)-H(23A)	109.5
C(22)-C(23)-H(23B)	109.5	C(22)-C(23)-H(23C)	109.5
H(23A)-C(23)-H(23B)	109.5	H(23A)-C(23)-H(23C)	109.5
H(23B)-C(23)-H(23C)	109.5	C(22)-C(24)-H(24A)	109.5
C(22)-C(24)-H(24B)	109.5	C(22)-C(24)-H(24C)	109.5
H(24A)-C(24)-H(24B)	109.5	H(24A)-C(24)-H(24C)	109.5
H(24B)-C(24)-H(24C)	109.5	C(21)-C(25)-H(25)	107.8
C(21)-C(25)-C(26)	111.01(13)	C(21)-C(25)-C(27)	113.44(13)
C(26)-C(25)-H(25)	107.8	C(27)-C(25)-H(25)	107.8
C(27)-C(25)-C(26)	108.64(14)	C(25)-C(26)-H(26A)	109.5
C(25)-C(26)-H(26B)	109.5	C(25)-C(26)-H(26C)	109.5
H(26A)-C(26)-H(26B)	109.5	H(26A)-C(26)-H(26C)	109.5
H(26B)-C(26)-H(26C)	109.5	C(25)-C(27)-H(27A)	109.5
C(25)-C(27)-H(27B)	109.5	C(25)-C(27)-H(27C)	109.5
H(27A)-C(27)-H(27B)	109.5	H(27A)-C(27)-H(27C)	109.5
H(27B)-C(27)-H(27C)	109.5	C(29)-C(28)-Ru(1)	73.69(9)
C(29)-C(28)-C(33)	120.16(17)	C(29)-C(28)-C(34)	120.6(2)
C(33)-C(28)-Ru(1)	70.32(10)	C(33)-C(28)-C(34)	119.23(19)
C(34)-C(28)-Ru(1)	127.78(12)	Ru(1)-C(29)-H(29)	134.0

C(28)-C(29)-Ru(1)	70.73(9)	C(28)-C(29)-H(29)	120.4
C(30)-C(29)-Ru(1)	68.07(9)	C(30)-C(29)-C(28)	119.18(16)
C(30)-C(29)-H(29)	120.4	Ru(1)-C(30)-H(30)	126.7
C(29)-C(30)-Ru(1)	76.03(9)	C(29)-C(30)-H(30)	119.3
C(29)-C(30)-C(31)	121.48(16)	C(31)-C(30)-Ru(1)	69.97(10)
C(31)-C(30)-H(30)	119.3	Ru(1)-C(31)-H(31)	127.9
C(30)-C(31)-Ru(1)	72.57(10)	C(30)-C(31)-H(31)	120.8
C(30)-C(31)-C(32)	118.39(18)	C(32)-C(31)-Ru(1)	70.79(10)
C(32)-C(31)-H(31)	120.8	Ru(1)-C(32)-H(32)	126.9
C(31)-C(32)-Ru(1)	71.09(10)	C(31)-C(32)-H(32)	119.9
C(33)-C(32)-Ru(1)	74.04(10)	C(33)-C(32)-C(31)	120.22(17)
C(33)-C(32)-H(32)	119.9	Ru(1)-C(33)-H(33)	130.2
C(28)-C(33)-Ru(1)	73.35(9)	C(28)-C(33)-H(33)	119.8
C(32)-C(33)-Ru(1)	69.15(10)	C(32)-C(33)-C(28)	120.36(16)
C(32)-C(33)-H(33)	119.8	C(28)-C(34)-H(34A)	109.5
C(28)-C(34)-H(34B)	109.5	C(28)-C(34)-H(34C)	109.5
H(34A)-C(34)-H(34B)	109.5	H(34A)-C(34)-H(34C)	109.5
H(34B)-C(34)-H(34C)	109.5	H(91A)-C(91)-H(91B)	109.5
H(91A)-C(91)-H(91C)	109.5	H(91B)-C(91)-H(91C)	109.5
C(92)-C(91)-H(91A)	109.5	C(92)-C(91)-H(91B)	109.5
C(92)-C(91)-H(91C)	109.5	C(91)-C(92)-H(92A)	108.8
C(91)-C(92)-H(92B)	108.8	C(91)-C(92)-C(93)	113.70(17)
H(92A)-C(92)-H(92B)	107.7	C(93)-C(92)-H(92A)	108.8
C(93)-C(92)-H(92B)	108.8	C(92)-C(93)-H(93A)	108.8
C(92)-C(93)-H(93B)	108.8	C(92)-C(93)-C(94)	113.76(16)
H(93A)-C(93)-H(93B)	107.7	C(94)-C(93)-H(93A)	108.8
C(94)-C(93)-H(93B)	108.8	C(93)-C(94)-H(94A)	109.5
C(93)-C(94)-H(94B)	109.5	H(94A)-C(94)-H(94B)	108.0
C(95)-C(94)-C(93)	110.89(18)	C(95)-C(94)-H(94A)	109.5
C(95)-C(94)-H(94B)	109.5	C(94)-C(95)-H(95A)	109.5
C(94)-C(95)-H(95B)	109.5	C(94)-C(95)-H(95C)	109.5
H(95A)-C(95)-H(95B)	109.5	H(95A)-C(95)-H(95C)	109.5
H(95B)-C(95)-H(95C)	109.5		

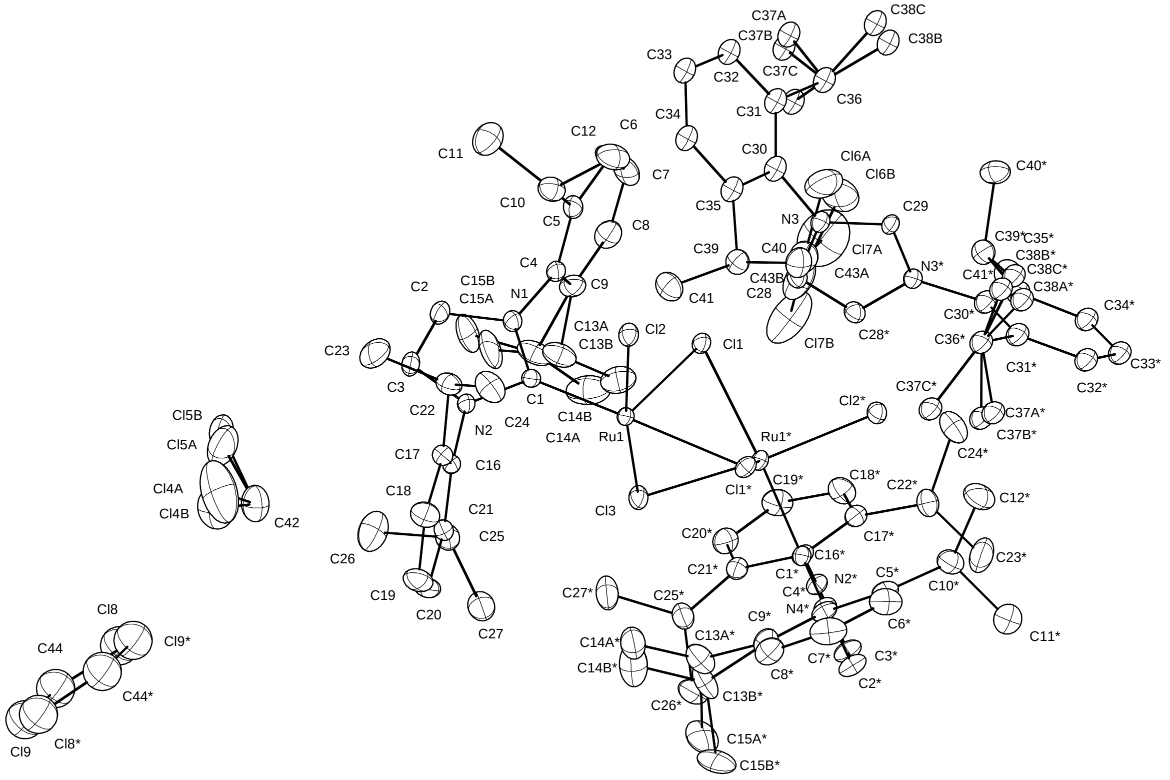

Figure S3. The molecular structure of complex **9·5(CH$_2$Cl$_2$)**; H-atoms removed for clarity.

X-ray Crystal Structure Analysis of complex 9·5(CH$_2$Cl$_2$): $C_{86}H_{121}Cl_{15}N_6Ru_2$, M_r = 1972.77 g mol^{-1}, orange prism, crystal size 0.037 x 0.032 x 0.011 mm^3, monoclinic, space group *C*2/c [15], a = 22.8887(10) Å, b = 15.1400(6) Å, c = 27.8398(12) Å, β = 94.106(2)°, V = 9622.7(7) Å^3, T = 100(2) K, Z = 4, D_{calc} = 1.362 g·cm^3, λ = 0.71073 Å, $\mu(Mo\text{-}K_\alpha)$ = 0.775 mm^{-1}, Gaussian absorption correction ($T_{\min}$ = 0.97086, $T_{\max}$ = 0.99056), Bruker-AXS Kappa Mach3 with APEX-II detector and IµS microfocus source, 1.467 < θ < 28.280°, 139529 measured reflections, 11946 independent reflections, 10004 reflections with $I > 2\sigma(I)$, R_{int} = 0.0543. The structure was solved by *SHELXT* and refined by full-matrix least-squares (*SHELXL*) against F^2 to R_1 = 0.0474 [$I > 2\sigma(I)$], wR_2 = 0.1126, 559 parameters, 10 restraints. **CCDC-2143699**

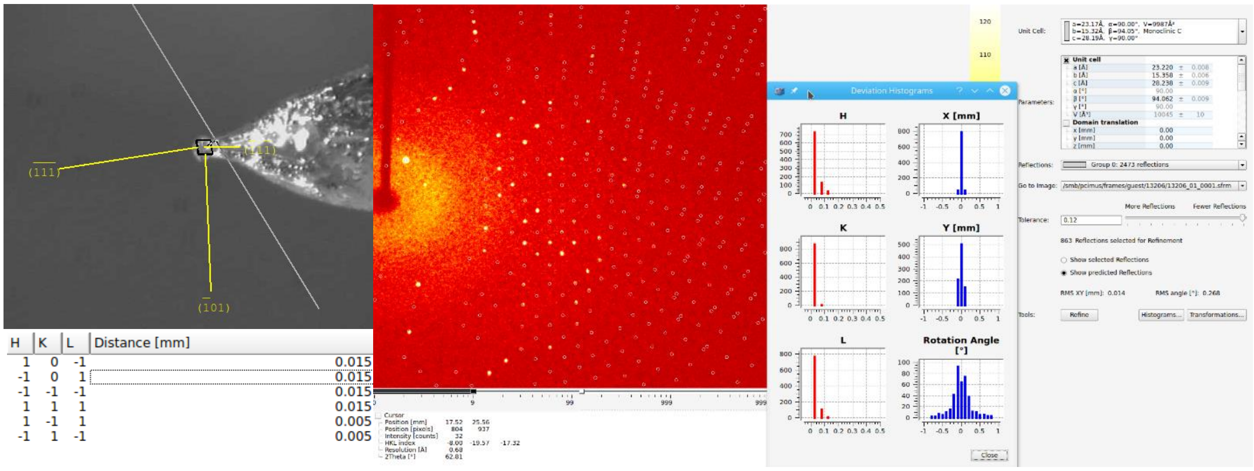

Figure S4. Crystal faces and unit cell determination/refinement of complex **9·5(CH₂Cl₂)**.

INTENSITY STATISTICS FOR DATASET

Resolution	#Data	#Theory	%Complete	Redundancy	Mean I	Mean I/s	Rmerge	Rsigma
Inf - 2.83	251	251	100.0	16.79	65.57	77.11	0.0240	0.0096
2.83 - 1.87	589	589	100.0	18.01	35.97	67.89	0.0289	0.0102
1.87 - 1.48	834	834	100.0	18.22	23.22	56.73	0.0356	0.0121
1.48 - 1.29	834	834	100.0	18.16	15.15	45.00	0.0458	0.0150
1.29 - 1.16	904	904	100.0	17.55	12.53	38.50	0.0550	0.0178
1.16 - 1.08	806	806	100.0	15.82	12.04	34.13	0.0604	0.0207
1.08 - 1.01	926	926	100.0	12.21	10.47	26.92	0.0689	0.0274
1.01 - 0.96	825	825	100.0	10.36	8.43	20.57	0.0836	0.0360
0.96 - 0.92	802	802	100.0	9.11	6.40	15.79	0.1012	0.0477
0.92 - 0.88	951	951	100.0	8.19	5.29	12.76	0.1219	0.0608
0.88 - 0.85	824	824	100.0	7.74	4.66	10.67	0.1362	0.0715
0.85 - 0.83	649	649	100.0	7.55	4.74	10.39	0.1334	0.0731
0.83 - 0.80	1035	1035	100.0	7.24	4.76	10.02	0.1435	0.0779
0.80 - 0.78	826	826	100.0	7.06	4.48	9.34	0.1562	0.0857
0.78 - 0.76	832	832	100.0	6.78	4.49	8.99	0.1609	0.0904
0.76 - 0.74	1007	1007	100.0	6.61	3.78	7.62	0.1890	0.1083
0.74 - 0.73	531	531	100.0	6.47	3.57	7.15	0.2047	0.1180
0.73 - 0.71	1160	1160	100.0	6.29	3.06	6.03	0.2267	0.1402
0.71 - 0.70	630	630	100.0	6.10	2.94	5.60	0.2454	0.1510
0.70 - 0.69	620	620	100.0	5.94	2.67	5.05	0.2638	0.1687
0.69 - 0.68	839	1028	81.6	3.33	2.27	3.58	0.2749	0.2844
0.78 - 0.68	5619	5808	96.7	5.85	3.27	6.34	0.2100	0.1397
Inf - 0.68	16675	16864	98.9	9.92	9.08	20.53	0.0598	0.0399

Three low-angle reflections [1 1 3] [2 0 6] [-1 1 7] where shadowed by the beamstop and removed from the data set before the final refinement cycles. The compound is highly air- and temperature sensitive. The investigated crystal was small and the structure contains several disordered regions. One isopropyl group of the anion exhibits a 60:40 % disorder over two positions. A second isopropyl group of the cation shows a disorder over three positions with 33.3 % occupancy for each component. All three CH_2Cl_2 solute molecules are disordered over two positions with varying occupancies (50:50 %, 75:25 % and 70:30 %).

A resolution cut off of 0.75 Å was applied to exclude poor diffraction intensity data at high resolution. The atomic displacement parameters of the disordered atoms were constrained to be equal: EADP C37C C37B C37A C38B C38A C38C C36 C31 C32 C33 C34 C35 C30 and EADP C44 Cl8 Cl9. The atomic distances in disorder solute molecules have been restrained to the following target values with an estimated standard deviation of 0.02: DFIX 1.76 Cl6A C43A, DFIX 1.76 Cl7A C43A, DFIX 1.76 Cl6B C43B, DFIX 1.76 Cl7B C43B. One CH_2Cl_2 molecule could only be satisfactorily described by isotropic displacement parameters.

High residual density of 3.18 eÅ^{-3} in the region of one of the octahedral coordination sites on each Ru atom in the absence of H_2 in the model is a potential sign for the presence of H_2 attached to Ru1. The quality of data set does not allow experimental localization of the attached H_2 molecule (see below).

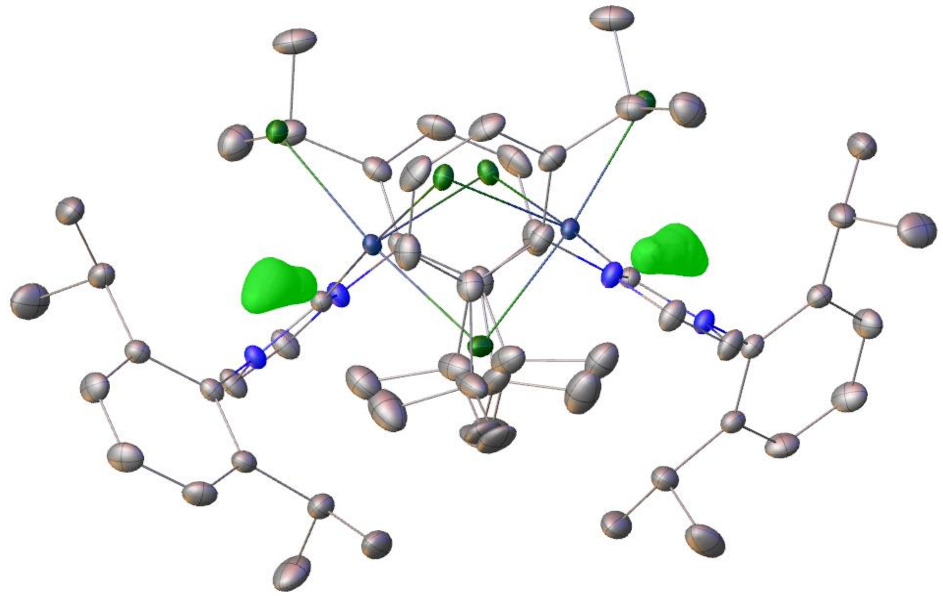

Figure S5. The molecular structure (carbon = grey, nitrogen = blue, chlorine = dark green, ruthenium = dark blue, positive electron density = light green) of the anion with residual electron density around the Ru atoms (drawn from: difference map Olex2, total min = –2.524 eÅ^{-3}, total max. = 3.673 eÅ^{-3}, 1.4 eÅ^{-3} and 0.1 Å resolution). Cation, solute molecules, disorder and H-atoms have been removed for clarity.

Supramolecular Features. Complex **9·5(CH_2Cl_2)** crystallizes in the centrosymmetric space group *C*2/c and the molecules are located about crystallographic special positions (two-fold rotational axes). Dense packing of the molecules can be inferred from the fact that there are no remaining voids in the unit cell (Mercury: probe radius = 1.2 Å, grid spacing = 0.7 Å).

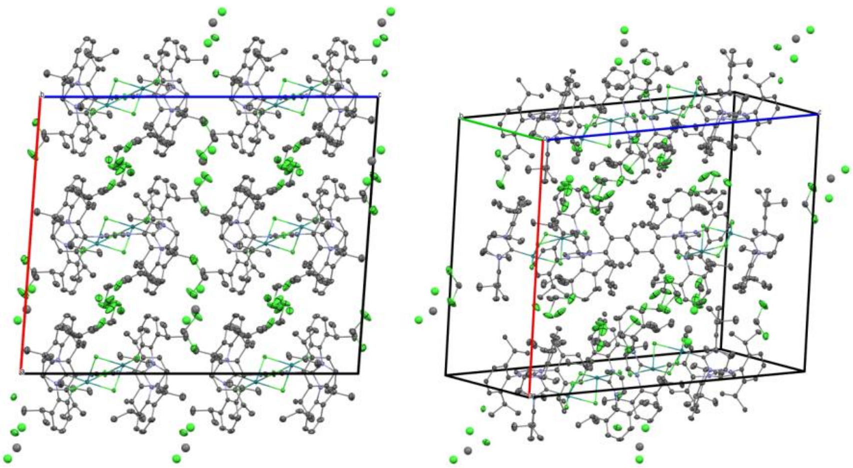

Figure S6. The packing of molecules relative to the unit cell viewed along the unique *b*-axis (left) and in a random orientation (right); H-atoms and disorder have been removed for clarity.

A short contact (3.39 Å) between C28 and Cl2 indicates an ionic interaction between $[HIPr]^+$ and the diruthenium anion. There are no hydrogen bond motifs found in this structure.

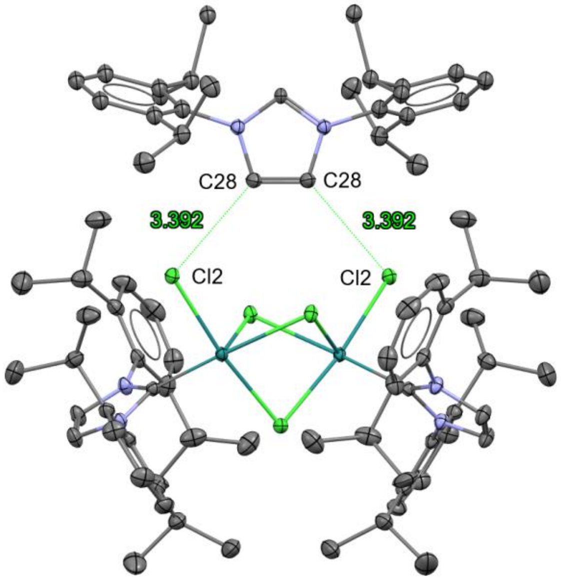

Figure S7. Ionic interaction via short contacts between C28 of $[HIPr]^+$ and Cl2 of the diruthenium anion; H-atoms, solute molecules and disorder have been removed for clarity.

Refinement of the Structure with Theoretically Calculated H_2 Positions. Inclusion of H_2 at theoretically calculated positions did not significantly improve the model. The presence of H_2 attached to Ru was shown by NMR spectroscopy and the H–H distance found to be ≈ 0.94 Å. Theoretical calculations on DFT level show a H–H bond distance of 0.93Å and where in good agreement with experimental observations. The two H-atoms were manually placed on the theoretically predicted positions at the Ru atom and restrained on these positions using EQIV and DFIX instructions: EQIV $1 1-X,+Y,0.5-Z, DFIX 1.635 0.0002 Hb Ru1, DFIX 0.93 0.0002 Ha Hb, DFIX 2.55 0.0002 Ha Cl2, DFIX 1.635 0.0002 Ha Ru1, DFIX 3.32 0.0002 Ha Cl1_$1, DFIX 3.25 0.0002 Hb Cl2. Refinement by full-matrix least-squares (*SHELXL*) against F^2 with Ru–H and H–H distances fixed at 1.635 Å and 0.930 Å, respectively, resulted in $R_1 = 0.0474$ [$I > 2\sigma(I)$], $wR_2 = 0.1126$, 559 parameters and a residual electron density peak of 3.16 eÅ^{-3} Å from Ru1 (drawn from: difference map Olex2, total min = –2.518 eÅ^{-3}, total max. = 3.648 eÅ^{-3}, 1.4 eÅ^{-3} and 0.1 Å resolution). The H-atoms attached to Ru1 could therefore not be satisfactorily resolved from the experimental X-ray dataset.

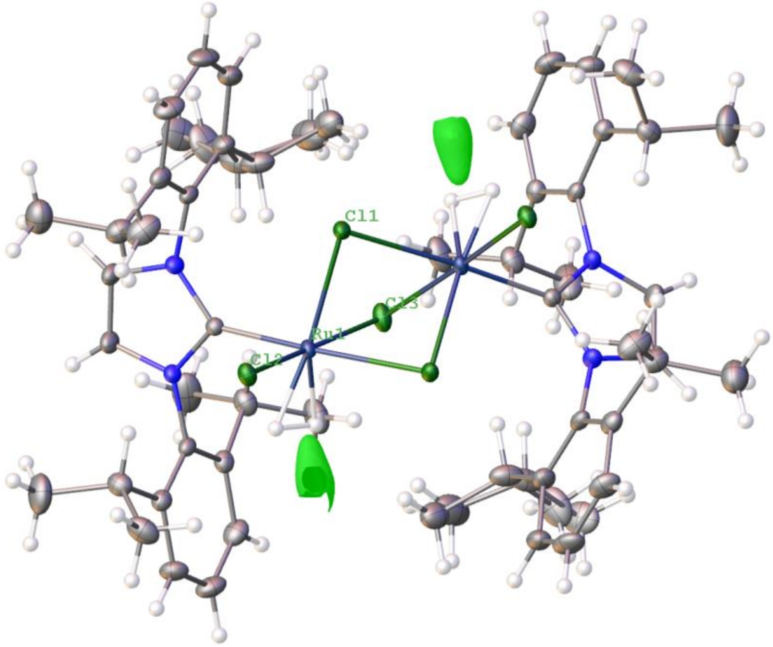

Figure S8. The molecular structure (carbon = grey, nitrogen = blue, chlorine = dark green, ruthenium = dark blue, hydrogen = white, positive electron density = light green, q-peaks = light brown) of the anionic part of the structure with manually placed H-atoms on the theoretically predicted positions at the Ru central atom. Unrefined electron density around the Ru atoms (drawn from: difference map Olex2, total min = –2.518 eÅ^{-3}, total max. = 3.648 eÅ^{-3}, 1.4 eÅ^{-3} and 0.1 Å resolution) indicates that the manually inserted H_2 moieties attached to the Ru atoms do not fully describe the residual electron density. Cationic part, solute molecules, disorders and other H-atoms are omitted for clarity.

Table S3. Crystal Data and Structure Refinement of Complex 9·5(CH_2Cl_2).

Identification code	**9·5(CH_2Cl_2)** (internal code: 13206)	
Empirical formula	C_{86} H_{121} Cl_{15} N_6 Ru_2	
Color	orange	
Formula weight	1972.77 g·mol^{-1}	
Temperature	100(2) K	
Wavelength	0.71073 Å	
Crystal system	Monoclinic	
Space group	*C*2/c, (no. 15)	
Unit cell dimensions	a = 22.8887(10) Å	α= 90°
	b = 15.1400(6) Å	β= 94.106(2)°
	c = 27.8398(12) Å	γ = 90°
Volume	9622.7(7) Å^3	
Z	4	
Density (calculated)	1.362 Mg·m^{-3}	
Absorption coefficient	0.775 mm^{-1}	
F(000)	4088 e	
Crystal size	0.037 x 0.032 x 0.011 mm^3	
θ range for data collection	1.467 to 28.280°.	
Index ranges	$-30 \le h \le 30$, $-20\le k \le 20$, $-37 \le l \le 37$	
Reflections collected	139529	
Independent reflections	11946 [R_{int} = 0.0543]	
Reflections with I>2σ(I)	10004	
Completeness to θ = 25.242°	100.0 %	
Absorption correction	Gaussian	
Max. and min. transmission	0.99056 and 0.97086	
Refinement method	Full-matrix least-squares on F^2	
Data / restraints / parameters	11946 / 10 / 559	
Goodness-of-fit on F^2	1.043	
Final R indices [I>2σ(I)]	R_1 = 0.0474	wR^2 = 0.1126
R indices (all data)	R_1 = 0.0598	wR^2 = 0.1198
Extinction coefficient	n/a	
Largest diff. peak and hole	3.156 and -2.096 e·Å^{-3}	

Table S4. Complex 9·5(CH_2Cl_2): Bond lengths [Å] and Angles [°].

Ru(1)-HA	1.6350(2)	Ru(1)-HB	1.6350(2)
Ru(1)-Cl(1)#1	2.5302(7)	Ru(1)-Cl(1)	2.4288(7)
Ru(1)-Cl(2)	2.3941(7)	Ru(1)-Cl(3)	2.4118(7)
Ru(1)-C(1)	2.004(3)	N(1)-C(1)	1.379(4)
N(1)-C(2)	1.386(4)	N(1)-C(4)	1.449(4)
N(2)-C(1)	1.379(3)	N(2)-C(3)	1.390(4)
N(2)-C(16)	1.439(4)	C(2)-H(2)	0.9500
C(2)-C(3)	1.341(4)	C(3)-H(3)	0.9500
C(4)-C(5)	1.400(4)	C(4)-C(9)	1.396(4)
C(5)-C(6)	1.397(4)	C(5)-C(10)	1.520(5)
C(6)-H(6)	0.9500	C(6)-C(7)	1.382(5)
C(7)-H(7)	0.9500	C(7)-C(8)	1.378(5)
C(8)-H(8)	0.9500	C(8)-C(9)	1.388(4)
C(9)-C(13A)	1.464(19)	C(9)-C(13B)	1.64(2)
C(10)-H(10)	1.0000	C(10)-C(11)	1.536(5)
C(10)-C(12)	1.531(5)	C(11)-H(11A)	0.9800
C(11)-H(11B)	0.9800	C(11)-H(11C)	0.9800
C(12)-H(12A)	0.9800	C(12)-H(12B)	0.9800
C(12)-H(12C)	0.9800	C(13A)-H(13A)	1.0000
C(13A)-C(14A)	1.54(2)	C(13A)-C(15A)	1.518(14)
C(13B)-H(13B)	1.0000	C(13B)-C(14B)	1.51(3)
C(13B)-C(15B)	1.53(2)	C(14A)-H(14A)	0.9800
C(14A)-H(14B)	0.9800	C(14A)-H(14C)	0.9800
C(14B)-H(14D)	0.9800	C(14B)-H(14E)	0.9800
C(14B)-H(14F)	0.9800	C(15A)-H(15A)	0.9800
C(15A)-H(15B)	0.9800	C(15A)-H(15C)	0.9800
C(15B)-H(15D)	0.9800	C(15B)-H(15E)	0.9800
C(15B)-H(15F)	0.9800	C(16)-C(17)	1.398(4)
C(16)-C(21)	1.401(4)	C(17)-C(18)	1.394(4)
C(17)-C(22)	1.518(4)	C(18)-H(18)	0.9500
C(18)-C(19)	1.379(5)	C(19)-H(19)	0.9500
C(19)-C(20)	1.389(5)	C(20)-H(20)	0.9500
C(20)-C(21)	1.388(4)	C(21)-C(25)	1.522(4)

C(22)-H(22)	1.0000	C(22)-C(23)	1.531(5)
C(22)-C(24)	1.525(5)	C(23)-H(23A)	0.9800
C(23)-H(23B)	0.9800	C(23)-H(23C)	0.9800
C(24)-H(24A)	0.9800	C(24)-H(24B)	0.9800
C(24)-H(24C)	0.9800	C(25)-H(25)	1.0000
C(25)-C(26)	1.530(5)	C(25)-C(27)	1.523(5)
C(26)-H(26A)	0.9800	C(26)-H(26B)	0.9800
C(26)-H(26C)	0.9800	C(27)-H(27A)	0.9800
C(27)-H(27B)	0.9800	C(27)-H(27C)	0.9800
Cl(4A)-C(42)	1.820(17)	Cl(4B)-C(42)	1.724(8)
Cl(5A)-C(42)	1.60(4)	Cl(5B)-C(42)	1.824(15)
Cl(8)-C(44)	1.765(11)	Cl(9)-C(44)	1.745(11)
C(42)-H	1.08(5)	C(42)-HC	0.93(5)
C(44)-HD	0.9900	C(44)-HE	0.9900
Cl(6A)-C(43A)	1.770(7)	Cl(6B)-C(43B)	1.769(16)
Cl(7A)-C(43A)	1.761(6)	Cl(7B)-C(43B)	1.737(15)
C(43A)-HF	0.9900	C(43A)-HG	0.9900
C(43B)-HH	0.9900	C(43B)-HI	0.9900
N(3)-C(28)	1.388(4)	N(3)-C(29)	1.331(3)
N(3)-C(30)	1.454(4)	C(38C)-HJ	0.9800
C(38C)-HK	0.9800	C(38C)-HL	0.9800
C(38C)-C(36)	1.527(12)	C(28)-C(28)#1	1.345(6)
C(28)-HM	0.9500	C(29)-HN	0.9500
C(30)-C(31)	1.395(4)	C(30)-C(35)	1.400(4)
C(31)-C(32)	1.396(4)	C(31)-C(36)	1.518(4)
C(32)-HO	0.9500	C(32)-C(33)	1.381(4)
C(33)-HP	0.9500	C(33)-C(34)	1.387(4)
C(34)-HQ	0.9500	C(34)-C(35)	1.391(4)
C(35)-C(39)	1.516(4)	C(36)-C(37C)	1.587(10)
C(36)-C(37B)	1.519(14)	C(36)-C(37A)	1.500(14)
C(36)-C(38B)	1.458(13)	C(36)-C(38A)	1.678(12)
C(37C)-HR	0.9800	C(37C)-HS	0.9800
C(37C)-HT	0.9800	C(37B)-HU	0.9800
C(37B)-HV	0.9800	C(37B)-HW	0.9800
C(37A)-HX	0.9800	C(37A)-HY	0.9800

C(37A)-HZ	0.9800	C(38B)-H(1)	0.9800
C(38B)-H(4)	0.9800	C(38B)-H(5)	0.9800
C(38A)-H(9)	0.9800	C(38A)-H(11)	0.9800
C(38A)-H(12)	0.9800	C(39)-H(13)	1.0000
C(39)-C(40)	1.543(4)	C(39)-C(41)	1.530(4)
C(40)-H(14)	0.9800	C(40)-H(15)	0.9800
C(40)-H(16)	0.9800	C(41)-H(17)	0.9800
C(41)-H(21)	0.9800	C(41)-H(23)	0.9800

HA-Ru(1)-HB	33.046(8)	Cl(1)#1-Ru(1)-HA	103.61(3)
Cl(1)-Ru(1)-HA	168.37(3)	Cl(1)#1-Ru(1)-HB	88.40(5)
Cl(1)-Ru(1)-HB	158.02(3)	Cl(1)-Ru(1)-Cl(1)#1	78.73(3)
Cl(2)-Ru(1)-HA	75.94(2)	Cl(2)-Ru(1)-HB	106.00(3)
Cl(2)-Ru(1)-Cl(1)	92.61(2)	Cl(2)-Ru(1)-Cl(1)#1	93.38(2)
Cl(2)-Ru(1)-Cl(3)	172.85(2)	Cl(3)-Ru(1)-HA	108.99(3)
Cl(3)-Ru(1)-HB	77.71(4)	Cl(3)-Ru(1)-Cl(1)#1	80.51(2)
Cl(3)-Ru(1)-Cl(1)	82.60(2)	C(1)-Ru(1)-HA	78.40(8)
C(1)-Ru(1)-HB	92.22(9)	C(1)-Ru(1)-Cl(1)	99.76(8)
C(1)-Ru(1)-Cl(1)#1	176.98(8)	C(1)-Ru(1)-Cl(2)	89.30(8)
C(1)-Ru(1)-Cl(3)	96.73(8)	Ru(1)-Cl(1)-Ru(1)#1	82.33(2)
Ru(1)#1-Cl(3)-Ru(1)	85.20(3)	C(1)-N(1)-C(2)	111.7(2)
C(1)-N(1)-C(4)	130.7(2)	C(2)-N(1)-C(4)	117.5(2)
C(1)-N(2)-C(3)	112.0(2)	C(1)-N(2)-C(16)	128.1(2)
C(3)-N(2)-C(16)	119.9(2)	N(1)-C(1)-Ru(1)	131.5(2)
N(1)-C(1)-N(2)	102.4(2)	N(2)-C(1)-Ru(1)	126.1(2)
N(1)-C(2)-H(2)	126.3	C(3)-C(2)-N(1)	107.3(3)
C(3)-C(2)-H(2)	126.3	N(2)-C(3)-H(3)	126.7
C(2)-C(3)-N(2)	106.5(3)	C(2)-C(3)-H(3)	126.7
C(5)-C(4)-N(1)	118.2(3)	C(9)-C(4)-N(1)	118.7(3)
C(9)-C(4)-C(5)	122.7(3)	C(4)-C(5)-C(10)	122.8(3)
C(6)-C(5)-C(4)	117.2(3)	C(6)-C(5)-C(10)	120.0(3)
C(5)-C(6)-H(6)	119.4	C(7)-C(6)-C(5)	121.1(3)
C(7)-C(6)-H(6)	119.4	C(6)-C(7)-H(7)	119.9
C(8)-C(7)-C(6)	120.1(3)	C(8)-C(7)-H(7)	119.9
C(7)-C(8)-H(8)	119.4	C(7)-C(8)-C(9)	121.3(3)

C(9)-C(8)-H(8)	119.4	C(4)-C(9)-C(13A)	123.1(8)
C(4)-C(9)-C(13B)	119.9(10)	C(8)-C(9)-C(4)	117.6(3)
C(8)-C(9)-C(13A)	119.1(8)	C(8)-C(9)-C(13B)	120.9(10)
C(5)-C(10)-H(10)	108.1	C(5)-C(10)-C(11)	110.3(3)
C(5)-C(10)-C(12)	111.7(3)	C(11)-C(10)-H(10)	108.1
C(12)-C(10)-H(10)	108.1	C(12)-C(10)-C(11)	110.3(3)
C(10)-C(11)-H(11A)	109.5	C(10)-C(11)-H(11B)	109.5
C(10)-C(11)-H(11C)	109.5	H(11A)-C(11)-H(11B)	109.5
H(11A)-C(11)-H(11C)	109.5	H(11B)-C(11)-H(11C)	109.5
C(10)-C(12)-H(12A)	109.5	C(10)-C(12)-H(12B)	109.5
C(10)-C(12)-H(12C)	109.5	H(12A)-C(12)-H(12B)	109.5
H(12A)-C(12)-H(12C)	109.5	H(12B)-C(12)-H(12C)	109.5
C(9)-C(13A)-H(13A)	108.5	C(9)-C(13A)-C(14A)	114.4(10)
C(9)-C(13A)-C(15A)	107.7(12)	C(14A)-C(13A)-H(13A)	108.5
C(15A)-C(13A)-H(13A)	108.5	C(15A)-C(13A)-C(14A)	109.1(13)
C(9)-C(13B)-H(13B)	106.9	C(14B)-C(13B)-C(9)	109.5(14)
C(14B)-C(13B)-H(13B)	106.9	C(14B)-C(13B)-C(15B)	112.1(18)
C(15B)-C(13B)-C(9)	114.1(19)	C(15B)-C(13B)-H(13B)	106.9
C(13A)-C(14A)-H(14A)	109.5	C(13A)-C(14A)-H(14B)	109.5
C(13A)-C(14A)-H(14C)	109.5	H(14A)-C(14A)-H(14B)	109.5
H(14A)-C(14A)-H(14C)	109.5	H(14B)-C(14A)-H(14C)	109.5
C(13B)-C(14B)-H(14D)	109.5	C(13B)-C(14B)-H(14E)	109.5
C(13B)-C(14B)-H(14F)	109.5	H(14D)-C(14B)-H(14E)	109.5
H(14D)-C(14B)-H(14F)	109.5	H(14E)-C(14B)-H(14F)	109.5
C(13A)-C(15A)-H(15A)	109.5	C(13A)-C(15A)-H(15B)	109.5
C(13A)-C(15A)-H(15C)	109.5	H(15A)-C(15A)-H(15B)	109.5
H(15A)-C(15A)-H(15C)	109.5	H(15B)-C(15A)-H(15C)	109.5
C(13B)-C(15B)-H(15D)	109.5	C(13B)-C(15B)-H(15E)	109.5
C(13B)-C(15B)-H(15F)	109.5	H(15D)-C(15B)-H(15E)	109.5
H(15D)-C(15B)-H(15F)	109.5	H(15E)-C(15B)-H(15F)	109.5
C(17)-C(16)-N(2)	118.7(2)	C(17)-C(16)-C(21)	122.8(3)
C(21)-C(16)-N(2)	118.4(3)	C(16)-C(17)-C(22)	122.7(3)
C(18)-C(17)-C(16)	117.3(3)	C(18)-C(17)-C(22)	119.9(3)
C(17)-C(18)-H(18)	119.4	C(19)-C(18)-C(17)	121.2(3)
C(19)-C(18)-H(18)	119.4	C(18)-C(19)-H(19)	119.9

C(18)-C(19)-C(20)	120.2(3)	C(20)-C(19)-H(19)	119.9
C(19)-C(20)-H(20)	119.5	C(21)-C(20)-C(19)	121.0(3)
C(21)-C(20)-H(20)	119.5	C(16)-C(21)-C(25)	121.5(3)
C(20)-C(21)-C(16)	117.5(3)	C(20)-C(21)-C(25)	121.0(3)
C(17)-C(22)-H(22)	108.0	C(17)-C(22)-C(23)	109.5(3)
C(17)-C(22)-C(24)	112.8(3)	C(23)-C(22)-H(22)	108.0
C(24)-C(22)-H(22)	108.0	C(24)-C(22)-C(23)	110.4(3)
C(22)-C(23)-H(23A)	109.5	C(22)-C(23)-H(23B)	109.5
C(22)-C(23)-H(23C)	109.5	H(23A)-C(23)-H(23B)	109.5
H(23A)-C(23)-H(23C)	109.5	H(23B)-C(23)-H(23C)	109.5
C(22)-C(24)-H(24A)	109.5	C(22)-C(24)-H(24B)	109.5
C(22)-C(24)-H(24C)	109.5	H(24A)-C(24)-H(24B)	109.5
H(24A)-C(24)-H(24C)	109.5	H(24B)-C(24)-H(24C)	109.5
C(21)-C(25)-H(25)	108.2	C(21)-C(25)-C(26)	109.3(3)
C(21)-C(25)-C(27)	113.4(3)	C(26)-C(25)-H(25)	108.2
C(27)-C(25)-H(25)	108.2	C(27)-C(25)-C(26)	109.4(3)
C(25)-C(26)-H(26A)	109.5	C(25)-C(26)-H(26B)	109.5
C(25)-C(26)-H(26C)	109.5	H(26A)-C(26)-H(26B)	109.5
H(26A)-C(26)-H(26C)	109.5	H(26B)-C(26)-H(26C)	109.5
C(25)-C(27)-H(27A)	109.5	C(25)-C(27)-H(27B)	109.5
C(25)-C(27)-H(27C)	109.5	H(27A)-C(27)-H(27B)	109.5
H(27A)-C(27)-H(27C)	109.5	H(27B)-C(27)-H(27C)	109.5
Cl(4A)-C(42)-H	116(3)	Cl(4A)-C(42)-HC	106(3)
Cl(4B)-C(42)-Cl(5B)	113.1(4)	Cl(4B)-C(42)-H	106(3)
Cl(4B)-C(42)-HC	111(3)	Cl(5A)-C(42)-Cl(4A)	110.2(13)
Cl(5A)-C(42)-H	96(3)	Cl(5A)-C(42)-HC	109(3)
Cl(5B)-C(42)-H	101(3)	Cl(5B)-C(42)-HC	106(3)
H-C(42)-HC	120(4)	Cl(8)-C(44)-HD	108.3
Cl(8)-C(44)-HE	108.3	Cl(9)-C(44)-Cl(8)	116.1(6)
Cl(9)-C(44)-HD	108.3	Cl(9)-C(44)-HE	108.3
HD-C(44)-HE	107.4	Cl(6A)-C(43A)-HF	109.5
Cl(6A)-C(43A)-HG	109.5	Cl(7A)-C(43A)-Cl(6A)	110.9(4)
Cl(7A)-C(43A)-HF	109.5	Cl(7A)-C(43A)-HG	109.5
HF-C(43A)-HG	108.0	Cl(6B)-C(43B)-HH	109.3
Cl(6B)-C(43B)-HI	109.3	Cl(7B)-C(43B)-Cl(6B)	111.5(10)

Cl(7B)-C(43B)-HH	109.3	Cl(7B)-C(43B)-HI	109.3
HH-C(43B)-HI	108.0	C(28)-N(3)-C(30)	125.1(2)
C(29)-N(3)-C(28)	108.6(2)	C(29)-N(3)-C(30)	126.2(3)
HJ-C(38C)-HK	109.5	HJ-C(38C)-HL	109.5
HK-C(38C)-HL	109.5	C(36)-C(38C)-HJ	109.5
C(36)-C(38C)-HK	109.5	C(36)-C(38C)-HL	109.5
N(3)-C(28)-HM	126.5	C(28)#1-C(28)-N(3)	107.10(15)
C(28)#1-C(28)-HM	126.5	N(3)#1-C(29)-N(3)	108.5(3)
N(3)#1-C(29)-HN	125.7	N(3)-C(29)-HN	125.7
C(31)-C(30)-N(3)	117.7(3)	C(31)-C(30)-C(35)	124.3(3)
C(35)-C(30)-N(3)	117.9(3)	C(30)-C(31)-C(32)	116.5(3)
C(30)-C(31)-C(36)	123.0(3)	C(32)-C(31)-C(36)	120.5(3)
C(31)-C(32)-HO	119.5	C(33)-C(32)-C(31)	121.0(3)
C(33)-C(32)-HO	119.5	C(32)-C(33)-HP	119.7
C(32)-C(33)-C(34)	120.7(3)	C(34)-C(33)-HP	119.7
C(33)-C(34)-HQ	119.5	C(33)-C(34)-C(35)	121.0(3)
C(35)-C(34)-HQ	119.5	C(30)-C(35)-C(39)	122.0(3)
C(34)-C(35)-C(30)	116.4(3)	C(34)-C(35)-C(39)	121.5(3)
C(38C)-C(36)-C(37C)	135.0(6)	C(31)-C(36)-C(38C)	109.1(5)
C(31)-C(36)-C(37C)	112.0(4)	C(31)-C(36)-C(37B)	111.9(6)
C(31)-C(36)-C(38A)	104.6(4)	C(37A)-C(36)-C(31)	113.1(6)
C(37A)-C(36)-C(38A)	112.2(7)	C(38B)-C(36)-C(31)	114.8(5)
C(38B)-C(36)-C(37B)	116.0(8)	C(36)-C(37C)-HR	109.5
C(36)-C(37C)-HS	109.5	C(36)-C(37C)-HT	109.5
HR-C(37C)-HS	109.5	HR-C(37C)-HT	109.5
HS-C(37C)-HT	109.5	C(36)-C(37B)-HU	109.5
C(36)-C(37B)-HV	109.5	C(36)-C(37B)-HW	109.5
HU-C(37B)-HV	109.5	HU-C(37B)-HW	109.5
HV-C(37B)-HW	109.5	C(36)-C(37A)-HX	109.5
C(36)-C(37A)-HY	109.5	C(36)-C(37A)-HZ	109.5
HX-C(37A)-HY	109.5	HX-C(37A)-HZ	109.5
HY-C(37A)-HZ	109.5	C(36)-C(38B)-H(1)	109.5
C(36)-C(38B)-H(4)	109.5	C(36)-C(38B)-H(5)	109.5
H(1)-C(38B)-H(4)	109.5	H(1)-C(38B)-H(5)	109.5
H(4)-C(38B)-H(5)	109.5	C(36)-C(38A)-H(9)	109.5

C(36)-C(38A)-H(11)	109.5	C(36)-C(38A)-H(12)	109.5
H(9)-C(38A)-H(11)	109.5	H(9)-C(38A)-H(12)	109.5
H(11)-C(38A)-H(12)	109.5	C(35)-C(39)-H(13)	107.8
C(35)-C(39)-C(40)	109.4(3)	C(35)-C(39)-C(41)	113.5(3)
C(40)-C(39)-H(13)	107.8	C(41)-C(39)-H(13)	107.8
C(41)-C(39)-C(40)	110.4(3)	C(39)-C(40)-H(14)	109.5
C(39)-C(40)-H(15)	109.5	C(39)-C(40)-H(16)	109.5
H(14)-C(40)-H(15)	109.5	H(14)-C(40)-H(16)	109.5
H(15)-C(40)-H(16)	109.5	C(39)-C(41)-H(17)	109.5
C(39)-C(41)-H(21)	109.5	C(39)-C(41)-H(23)	109.5
H(17)-C(41)-H(21)	109.5	H(17)-C(41)-H(23)	109.5
H(21)-C(41)-H(23)	109.5		

Symmetry transformations used to generate equivalent atoms:
#1 -x+1,y,-z+1/2

Database Survey: $Ru_2Cl_5X_2$

A Database (CSD Version 5.41, November 2019) survey was performed on 19th August 2020 in a search for related structures. The following search motive was used in ConQuest (Version 2020.1) program:

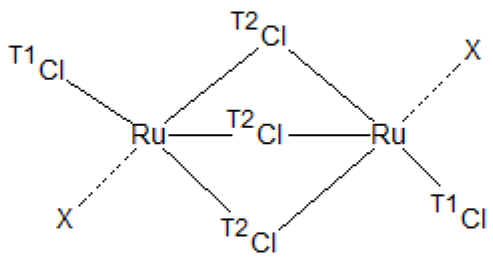

The initial search found 28 structures containing the predefined $Ru_2Cl_5X_2$ entity. Twelve of them appear to be a neutral complexes with mixed Ru oxidation states of +II and +III. The remaining sixteen structures show an ionic composition with Ru oxidation states of +II. These Ru species are negatively charged and interact with a various cationic entities.

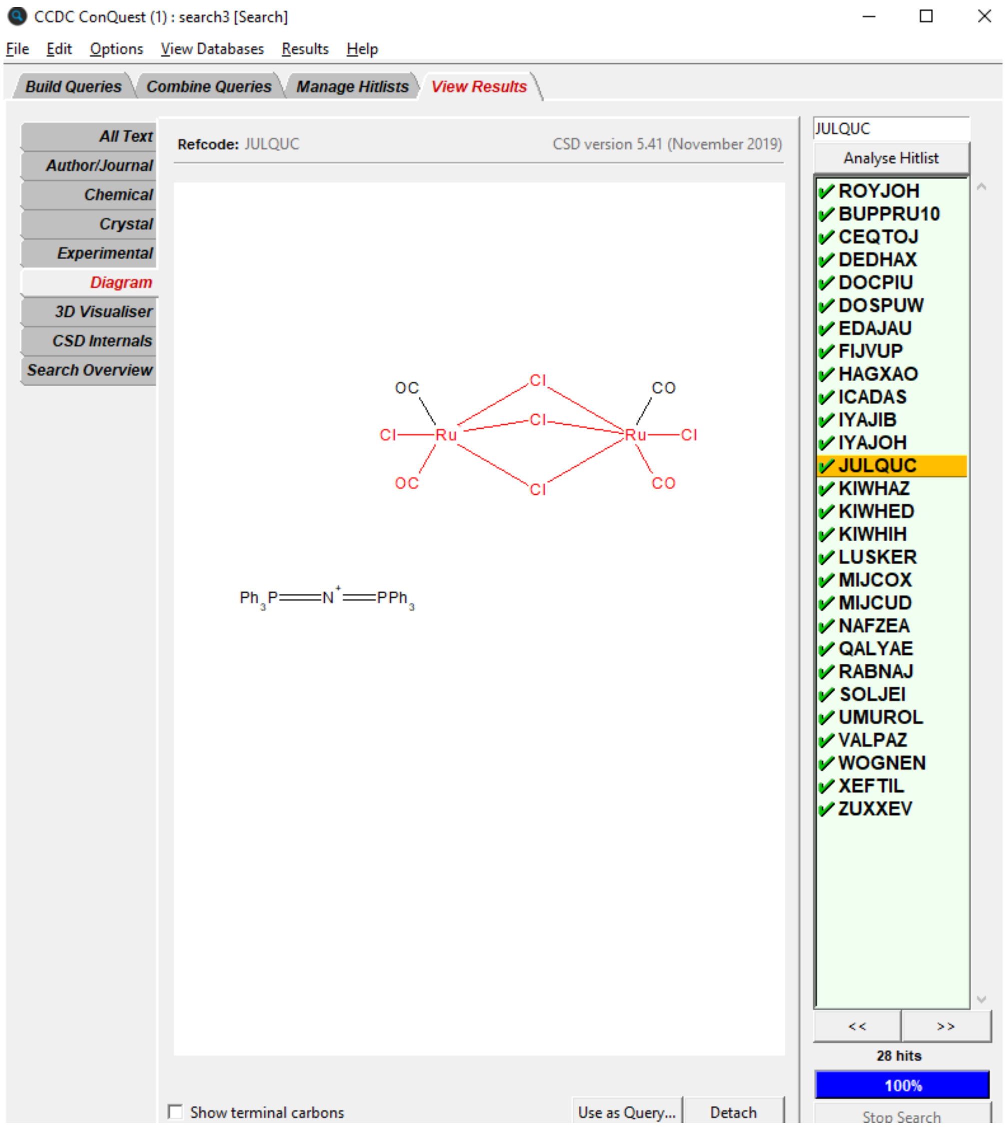

The most similar compounds are of the $[Ru_2X_5L_4]^-$ type. Most of them show a *cis*-arrangement of the non-bridging Cl atoms relative to the Ru···Ru vector. Short contacts indicate ionic interactions between the various escorting ammonium- or phosphonium cations and the pockets formed by two bridging and the non-bridging Cl atoms. Moreover, the observed Ru···Ru distances are similar to that in **9**·5(CH_2Cl_2) (3.265 Å).

However, it is pointed out that none of these known complexes contains any "vacant" coordination sites, to which H_2 could bind. Therefore their relationship to complex **9** discussed herein is merely structural rather than functional.

**ZUXXEV**[1] Ru1-Ru1B distance = 3.331 Å 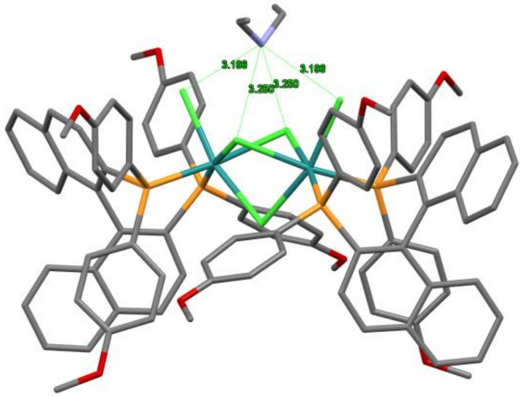	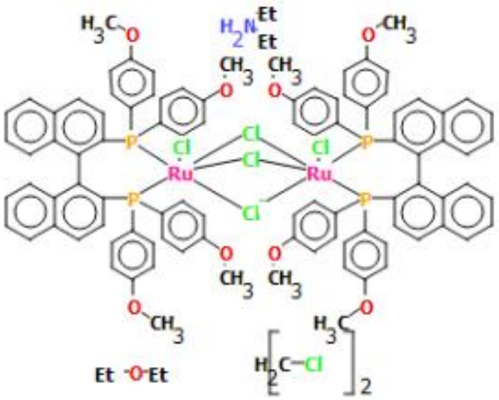
**XEFTIL**[2] Ru1-Ru2 distance = 3.271 Å; Ru3-Ru4 distance = 3.280 Å 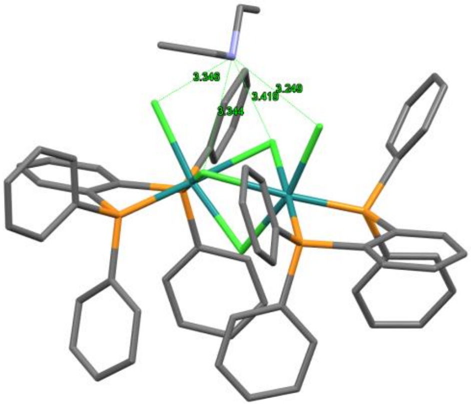	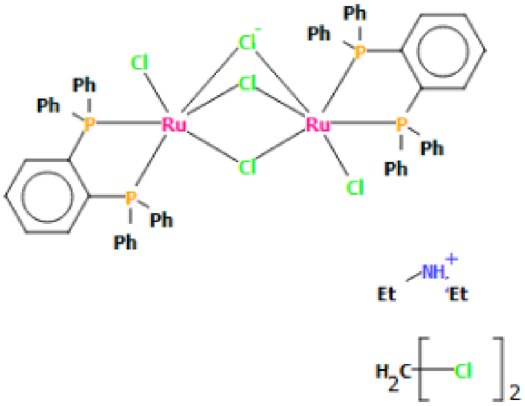
**LUSKER**[3] Ru1-Ru2 distance = 3.371 Å 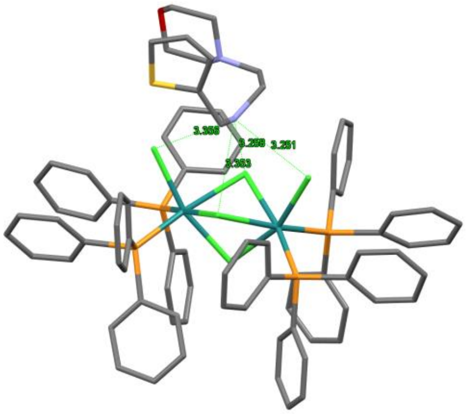	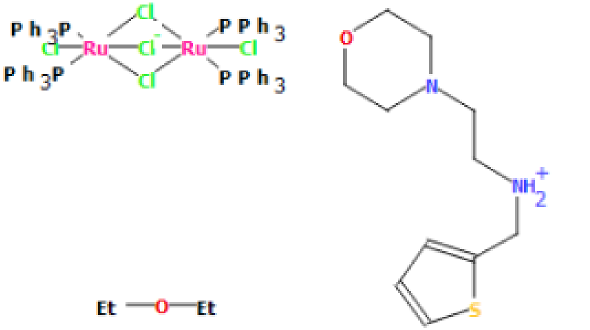

[1] T. Ohta, Y. Tonomura, K. Nozaki, H. Takaya, K. Mashima, *Organometallics* **1996**, 15, 1521 (DOI: 10.1021/om9508905)

[2] K. Mashima, T. Nakamura, Y. Matsuo, K. Tani, *J. Organomet.Chem.* **2000**, 607, 51 (DOI: 10.1016/S0022-328X(00)00192-3)

[3] P. A. Dub, B. L. Scott, J. C. Gordon, *Organometallics* **2015** *34*, 4464 (DOI: 10.1021/acs.organomet.5b00432)

**ICADAS**[4] Ru1-Ru2 distance = 3.370 Å 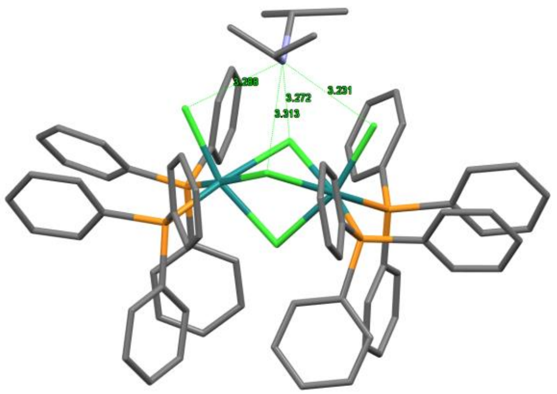	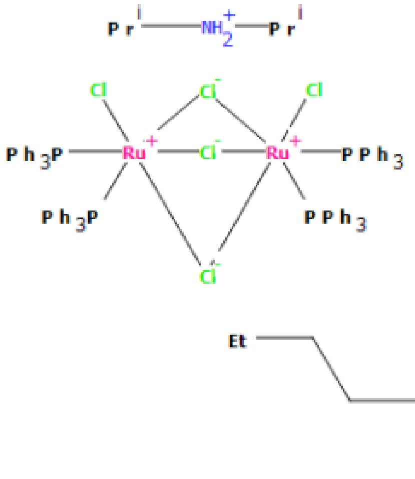
**FIJVUP**[5] Ru1-Ru1B distance = 3.365 Å 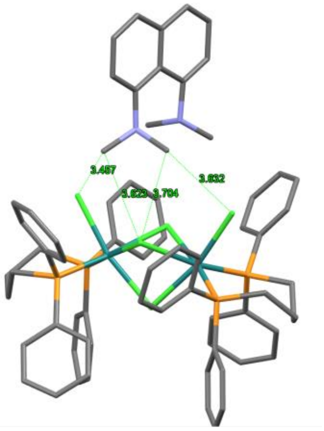	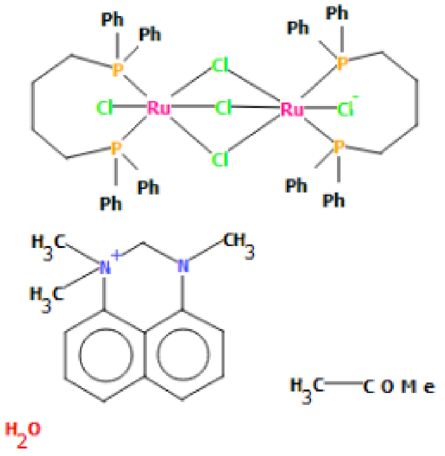
**DOSPUW**[6] Ru1-Ru2 distance = 3.258 Å 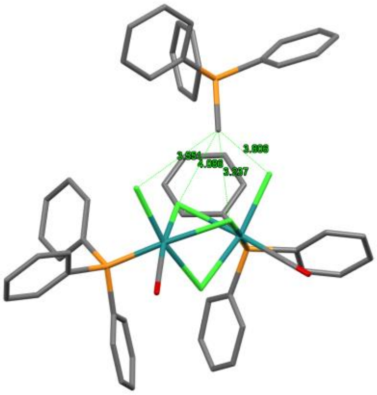	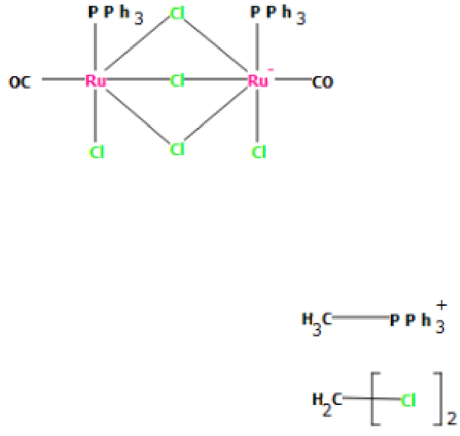

[4] D. B. Dell'Amico, F. Calderazzo, U. Englert, L. Labella, F. Marchetti, M. Specos, *Eur. J. Inorg. Chem.* **2004**, 3938 (DOI: 10.1002/ejic.200400014)

[5] S. N. Gamage, R. H. Morris, S. J. Rettig, D. C. Thackray, I. S. Thorburn, B. R. James, *Chem. Commun.* **1987**, 894 (DOI: 10.1039/c39870000894)

[6] R. A. Sanchez-Delgado, U. Thewalt, N. Valencia, A. Andriollo, R. L. Marquez-Silva, J. Puga, H. Schollhorn, H.-P. Klein, B. Fontal, *Inorg. Chem.* **1986**, 25, 1097 (DOI: 10.1021/ic00228a009)

Database Survey: Complexes Comprising a [Ru–iPr] Unit

A Database (CSD Version 5.42, September 2021) survey was performed on January 19, 2022, searching for related structures containing a ruthenium atom bonded to an iPr ligand. The following search motive was used in ConQuest (Version 2021.2.0) program:

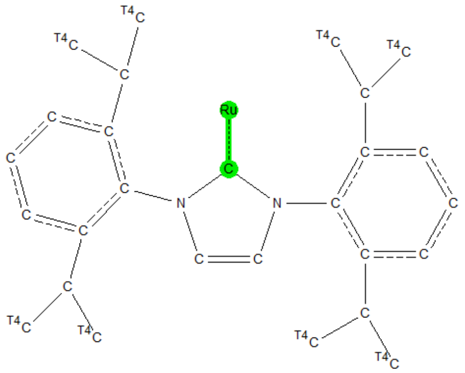

Searching without using predefined geometrical parameters (e.g. Ru–C distance) revealed in 58 structures but some of them are deposited without 3D coordinates. The initial search using predefined geometrical parameters found 52 structures containing the predefined Ru–(iPr) substructure. Within the dataset, 84 symmetry-independent bond lengths (caused by Z' > 1) are available. The average Ru–C distance is 2.098(38) Å. In complex **9**, the Ru-C distance of 2.004(2) Å appears to be the shortest described so far.

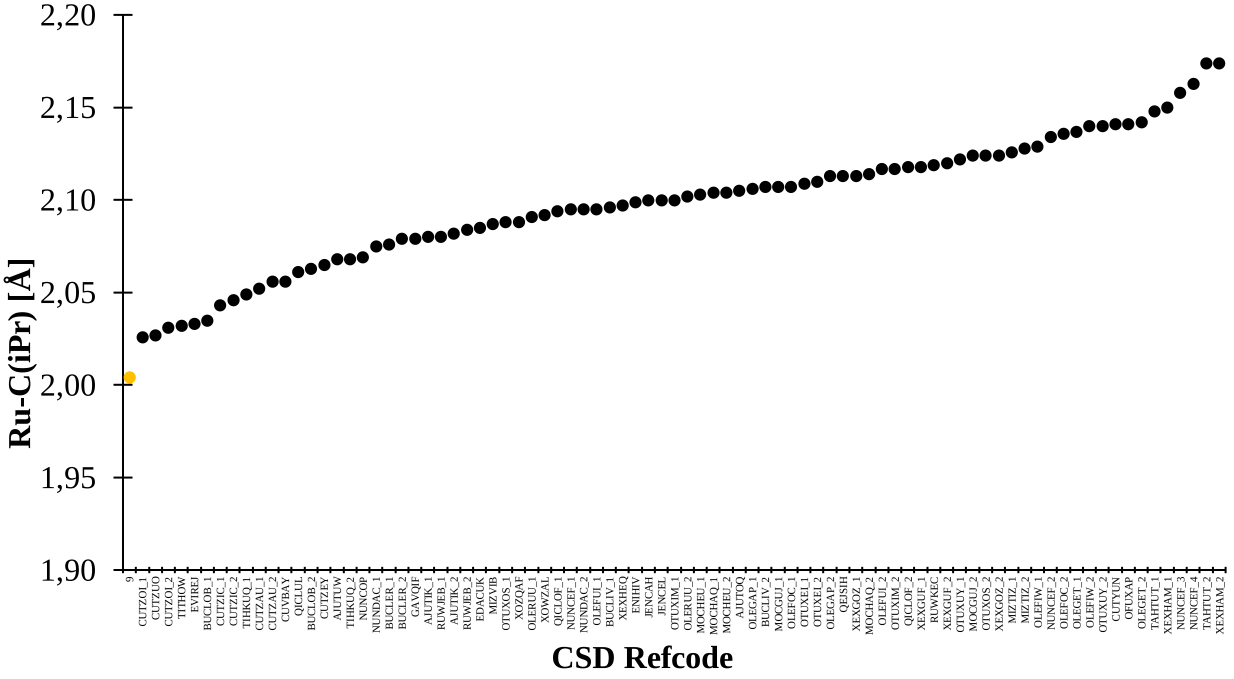

Figure S9. Comparison of the [Ru−C] bond lengths of crystallographically characterized complexes comprising this substructure (CCDC code); complex **9** is highlighted in orange.

Computed Coordinates of the Ruthenate Entity of Complex 9

C1	-0.5774400000	3.6642900000	1.8172100000
H2	0.1164300000	3.3299700000	1.0335900000
H3	-0.2468300000	3.2259300000	2.7712100000
H4	-0.4989300000	4.7595000000	1.9177100000
C5	-3.6498300000	3.1118900000	-1.9863000000
C6	-4.1582200000	-1.6456100000	1.3875600000
C7	-3.2505500000	2.7636900000	-0.6847100000
C8	-4.5029400000	2.2043300000	-2.8521200000
H9	-4.5095400000	1.2084800000	-2.3848600000
C10	-3.2391400000	0.3272900000	0.0964800000
C11	-2.9819300000	3.7185400000	2.5626800000
H12	-3.0275200000	4.8205500000	2.5607500000
H13	-2.6654400000	3.3962000000	3.5685300000
H14	-4.0012400000	3.3394400000	2.3946800000
C15	-4.6807900000	-2.7361000000	0.6638100000
C16	-5.9433100000	2.7153800000	-2.9420900000
H17	-6.4151500000	2.7959900000	-1.9503200000
H18	-6.5575000000	2.0314500000	-3.5501900000
H19	-5.9860600000	3.7123400000	-3.4124400000
C20	-5.2633700000	-2.5693300000	-0.7265500000
H21	-4.8452500000	-1.6442700000	-1.1532100000
C22	-4.2056700000	-0.1054600000	4.4603400000
H23	-5.1332500000	0.1691200000	3.9365100000
H24	-3.8522200000	0.7842400000	5.0059900000
H25	-4.4554200000	-0.8816300000	5.2027900000
C26	-2.0623500000	4.8357200000	-0.4378900000
H27	-1.4289100000	5.5054800000	0.1468900000
C28	-3.2353700000	4.3533900000	-2.4821300000
H29	-3.5243200000	4.6442000000	-3.4948400000
C30	-6.7875000000	-2.4334500000	-0.6625600000
H31	-7.2001600000	-2.2728700000	-1.6713800000
H32	-7.0979100000	-1.5832900000	-0.0364900000
H33	-7.2524700000	-3.3436100000	-0.2469800000
C34	-4.1571400000	-4.1590700000	2.5638700000
H35	-4.1515000000	-5.1507600000	3.0242500000
C36	-1.8256800000	-0.8956900000	4.2269400000
H37	-1.9562900000	-1.6532200000	5.0170300000
H38	-1.4570700000	0.0213200000	4.7123700000
H39	-1.0467100000	-1.2363900000	3.5303000000
C40	-4.8548800000	-3.6885200000	-1.6811100000
H41	-3.7610200000	-3.7863500000	-1.7303000000
H42	-5.1997600000	-3.4486700000	-2.6979400000
H43	-5.2903700000	-4.6626100000	-1.4013000000
C44	-4.6669900000	-3.9912500000	1.2814700000
H45	-5.0589700000	-4.8549500000	0.7400300000
C46	-2.4570500000	5.2110000000	-1.7177700000
H47	-2.1402700000	6.1742700000	-2.1267300000
C48	-3.8965500000	2.0097400000	-4.2411200000
H49	-3.8966600000	2.9426900000	-4.8294300000
H50	-4.4792700000	1.2616800000	-4.8010400000

H51	-2.8675000000	1.6330500000	-4.1595800000
C52	-3.6409000000	-1.7899100000	2.6911700000
C53	-2.0004100000	3.2124900000	1.5010500000
H54	-1.9859600000	2.1126100000	1.5346900000
C55	-5.0574500000	1.6639600000	0.4836800000
H56	-5.6402700000	2.5782900000	0.4298000000
C57	-5.3252600000	0.4686100000	1.0483700000
H58	-6.1928700000	0.1105100000	1.5937700000
C59	-3.6500600000	-3.0676700000	3.2597900000
H60	-3.2496800000	-3.2086900000	4.2662200000
C61	-3.1302100000	-0.6033300000	3.4892300000
H62	-2.9188000000	0.2057900000	2.7745800000
C63	-2.4453300000	3.6082900000	0.1049500000
Cl64	-0.0001300000	-0.0006300000	1.2112000000
Cl65	-0.5134500000	1.5222700000	-1.5685700000
Cl66	-2.8165000000	-0.8899600000	-2.6499500000
N67	-4.2206600000	-0.3327600000	0.8200100000
N68	-3.7963100000	1.5776200000	-0.0820700000
Ru69	-1.5807200000	-0.4784800000	-0.6262700000
H70	-1.9521200000	-1.7208900000	0.3743500000
H71	-2.0682900000	-2.0302700000	-0.4941200000
C72	0.5772000000	-3.6645700000	1.8170800000
H73	-0.1168200000	-3.3289200000	1.0341600000
H74	0.2473400000	-3.2269800000	2.7717000000
H75	0.4979000000	-4.7598200000	1.9164600000
C76	3.6478900000	-3.1108600000	-1.9874400000
C77	4.1591500000	1.6443500000	1.3890800000
C78	3.2494200000	-2.7634900000	-0.6853800000
C79	4.5005400000	-2.2027600000	-2.8531300000
H80	4.5075200000	-1.2072400000	-2.3851600000
C81	3.2391700000	-0.3274700000	0.0969800000
C82	2.9819400000	-3.7212400000	2.5615400000
H83	3.0267300000	-4.8232700000	2.5586000000
H84	2.6661000000	-3.3995700000	3.5678100000
H85	4.0014500000	-3.3427300000	2.3934600000
C86	4.6827000000	2.7349500000	0.6662200000
C87	5.9408100000	-2.7138500000	-2.9443300000
H88	6.4132200000	-2.7952000000	-1.9529000000
H89	6.5546900000	-2.0295300000	-3.5523100000
H90	5.9832100000	-3.7104900000	-3.4154200000
C91	5.2659200000	2.5687200000	-0.7239300000
H92	4.8477100000	1.6440100000	-1.1512500000
C93	4.2045900000	0.1022100000	4.4607200000
H94	5.1321200000	-0.1726000000	3.9369200000
H95	3.8504600000	-0.7876400000	5.0057000000
H96	4.4546600000	0.8777200000	5.2037600000
C97	2.0608600000	-4.8353900000	-0.4393500000
H98	1.4275900000	-5.5054200000	0.1453100000
C99	3.2328900000	-4.3519200000	-2.4839000000
H100	3.5211900000	-4.6420800000	-3.4969800000
C101	6.7899800000	2.4324000000	-0.6592600000
H102	7.2031000000	2.2721900000	-1.6679400000
H103	7.0998600000	1.5818700000	-0.0334300000
H104	7.2549800000	3.3422300000	-0.2430100000

C105	4.1589700000	4.1571200000	2.5668700000
H106	4.1536700000	5.1485500000	3.0278200000
C107	1.8251400000	0.8940800000	4.2272700000
H108	1.9560000000	1.6510500000	5.0178600000
H109	1.4558700000	-0.0230000000	4.7120400000
H110	1.0465400000	1.2356700000	3.5306600000
C111	4.8582000000	3.6885200000	-1.6781200000
H112	3.7643900000	3.7864800000	-1.7279500000
H113	5.2037100000	3.4492100000	-2.6948700000
H114	5.2936600000	4.6623800000	-1.3974700000
C115	4.6693300000	3.9897500000	1.2846100000
H116	5.0620800000	4.8535400000	0.7438600000
C117	2.4547900000	-5.2098800000	-1.7197100000
H118	2.1375500000	-6.1728000000	-2.1291600000
C119	3.8932700000	-2.0070800000	-4.2415800000
H120	3.8930100000	-2.9395700000	-4.8306300000
H121	4.4756200000	-1.2585600000	-4.8012800000
H122	2.8642600000	-1.6304700000	-4.1591000000
C123	3.6413400000	1.7881800000	2.6925500000
C124	2.0003700000	-3.2135300000	1.5007500000
H125	1.9867500000	-2.1136700000	1.5353700000
C126	5.0569300000	-1.6651300000	0.4833600000
H127	5.6393700000	-2.5796700000	0.4289100000
C128	5.3253000000	-0.4701800000	1.0486400000
H129	6.1931000000	-0.1127400000	1.5941700000
C130	3.6509400000	3.0656000000	3.2619100000
H131	3.2501900000	3.2062600000	4.2682400000
C132	3.1296800000	0.6014000000	3.4897000000
H133	2.9179300000	-0.2071100000	2.7744500000
C134	2.4444400000	-3.6084300000	0.1041300000
Cl135	0.5140200000	-1.5201200000	-1.5703600000
Cl136	2.8173600000	0.8933000000	-2.6480700000
N137	4.2210300000	0.3317900000	0.8207800000
N138	3.7957800000	-1.5779500000	-0.0822400000
Ru139	1.5810400000	0.4793900000	-0.6251900000
H140	1.9522100000	1.7205500000	0.3770600000
H141	2.0688300000	2.0309300000	-0.4909800000

General

All reactions were carried out under argon in flame-dried glassware, ensuring rigorously inert conditions. The solvents were purified by distillation over the indicated drying agents and were stored and handled under argon: CH_2Cl_2 (CaH_2), MeCN (CaH_2), pentane (Na/K alloy), THF (Na/K alloy).

Hydrogen gas (N50, ≥99.999 Vol.%) was purchased from AirLiquide and was used without further purification. Deuterium hydride (HD) (96 mol% DH, 98% atom % D) was purchased from Sigma-Aldrich. Hydrogen and HD were handled with standard balloon techniques.

NMR spectra were recorded on Bruker AVIII 400, AVIII 500 or AVneo 600 MHz NMR spectrometers at 298 K unless otherwise indicated; chemical shift (δ) given in ppm relative to TMS, coupling constants (J) in Hz. The solvent signals were used as references and the chemical shifts converted to the TMS scale (CD_2Cl_2: 5.32 ppm (^1H), 53.8 ppm (^{13}C); [D_3]-MeCN: 1.94 ppm (^1H), 118.26 ppm (^{13}C); [D_6]-acetone: 2.05 ppm (^1H), 29.8 ppm (^{13}C).

IR spectra: Alpha Platinum ATR (Bruker) at room temperature, wavenumbers ($\tilde{\nu}$) are given in cm^{-1}.

Mass spectrometry: MS (EI): Finnigan MAT 8200 (70 eV), ESI-MS: Bruker ESQ3000, accurate mass determinations: Bruker APEX III FT-MS (7 T magnet) or MAT 95 (Finnigan); GC-MS: Shimadzu GCMS-QP2010 Ultra instrument.

Photolysis experiments: PhotoRedOxBox TC (HepatoChem) equipped with an EvoluChem™ LED (365 nm, 18 W) (Figure S10). When the cooling function was used, water was the cooling agent (23 °C).

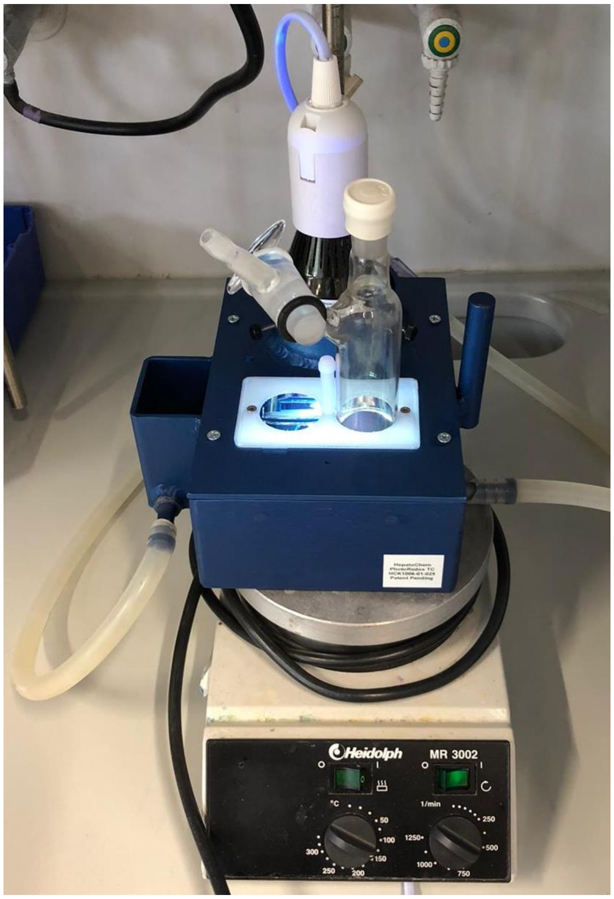

Figure S10. PhotoRedOxBox TC (HepatoChem)

Hydride Intermediates

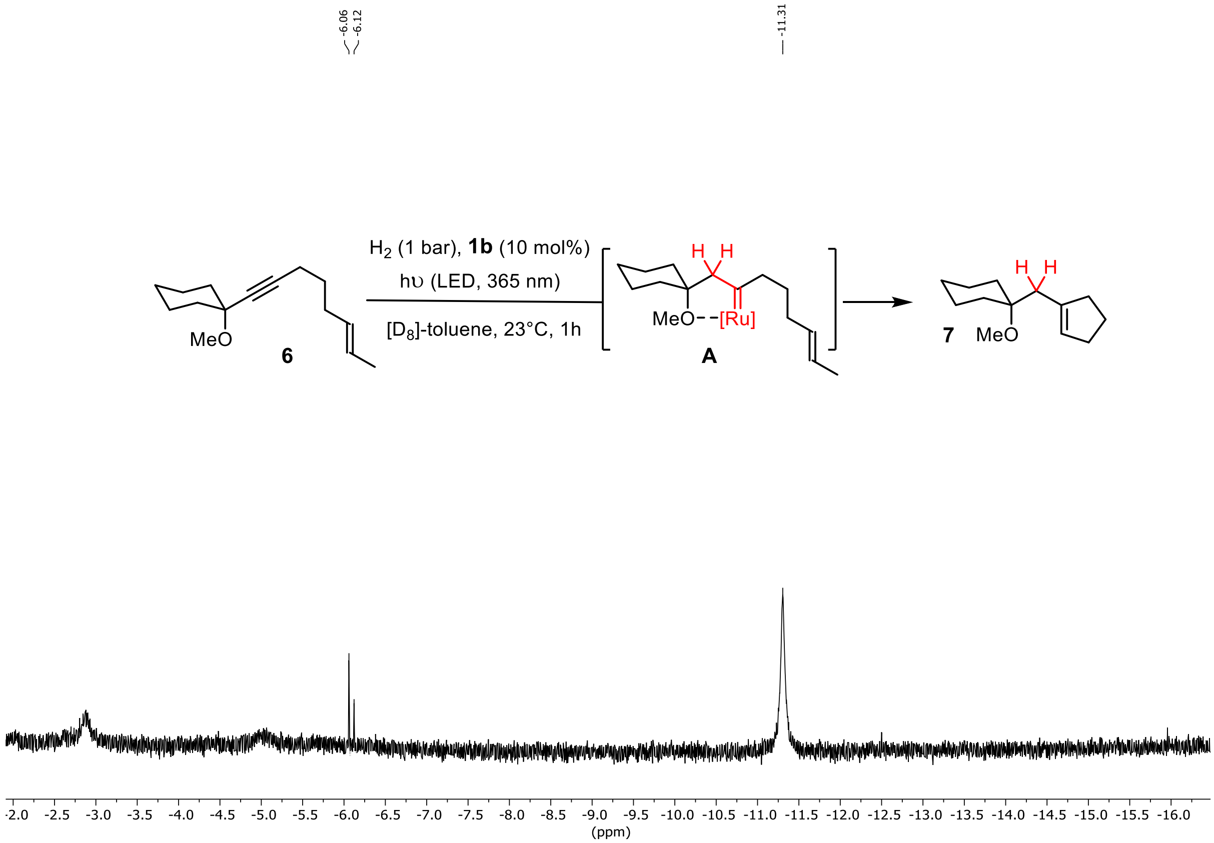

Figure S11. Hydride region of the ^{1}H NMR ([D_8]-toluene) spectrum recorded at the end of the light-driven *gem*-hydrogenation of enyne **6** with formation of cyclopentene **7**

Complex 9. In a flame-dried quartz Schlenk tube under argon, [(IPr)Ru(*p*-cymene)Cl_2] (**1b**)[1] (134 mg, 192 µmol) and IPrHCl (163 mg, 385 µmol) were suspended in toluene (15 mL). The Schlenk tube was closed

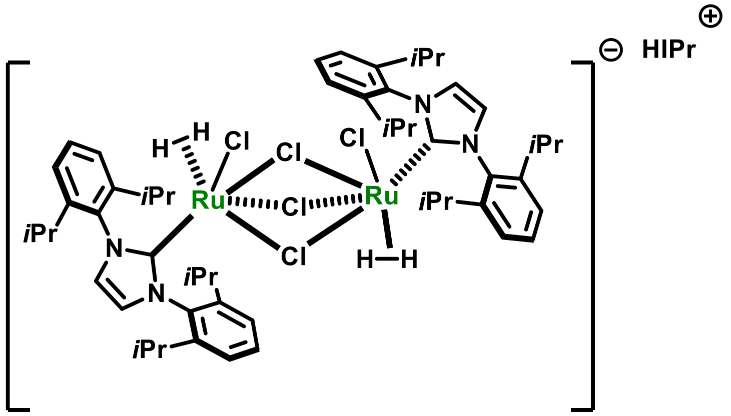

with a septum and then transferred into the PhotoRedOxBox. A hydrogen-filled balloon was connected to a needle, which was pierced through the septum. The Schlenk tube was flushed with hydrogen for 2 min through an outlet cannula (the cannula did not reach into the solution to ensure that only the head space of the tube was flushed). The exit cannula was removed but the hydrogen-filled balloon remained attached to the Schlenk tube to ensure constant hydrogen pressure throughout the reaction. The light

source was switched on and the reaction mixture was stirred for 20 min under hydrogen atmosphere. After this time, the yellow suspension was filtered via a filter cannula into another Schlenk tube and the filtrate was concentrated in vacuum. The remaining material was washed with pentane (3 x 10 mL). Drying under high vacuum afforded the title compound (110 mg, 73%) as a yellow solid. Orange crystals suitable for X-ray diffraction were grown by slow cooling of a saturated solution in CH_2Cl_2:C_6F_6 (1:1, *v/v*) from +20 to –50°C over the course of 2 weeks.

^{1}H NMR (600 MHz, 233 K, THF-[D_8]) δ 9.10 (s, 2H), 8.86 (s, 1H), 7.49 (t, *J* = 7.6 Hz, 2H), 7.43 (t, *J* = 7.6 Hz, 2H), 7.36 (d, *J* = 7.6 Hz, 2H), 7.32 (d, *J* = 6.5 Hz, 2H), 7.31 (d, *J* = 7.6 Hz, 2H), 7.28 (d, *J* = 7.7 Hz, 2H), 7.14 (s, 2H), 7.10 (t, *J* = 7.4 Hz, 2H), 6.96 (d, *J* = 7.8 Hz, 2H), 6.88 (d, *J* = 7.5 Hz, 2H), 6.84 (s, 2H), 3.31 (hept, *J* = 6.6 Hz, 2H), 2.96 (hept, *J* = 6.6 Hz, 2H), 2.85 (hept, *J* = 6.6 Hz, 2H), 2.80 (hept, *J* = 6.6 Hz, 2H), 2.61 (hept, *J* = 6.6 Hz, 2H), 2.33 (hept, *J* = 6.6 Hz, 2H), 1.33 (d, *J* = 6.3 Hz, 6H), 1.31 (d, *J* = 6.3 Hz, 6H), 1.24 (d, *J* = 6.5 Hz, 6H), 1.08 (d, *J* = 6.5 Hz, 6H), 1.06 (d, *J* = 6.9 Hz, 6H), 1.00 (s, 12H), 0.97 (d, *J* = 6.5 Hz, 6H), 0.91 – 0.86 (m, 12H), 0.82 (d, *J* = 6.5 Hz, 6H), 0.45 (d, *J* = 5.9 Hz, 6H), –11.54 (s, 4H). ^{13}C NMR (151 MHz, 233 K, THF-[D_8]) δ 189.6, 148.9, 148.2, 147.5, 146.8, 146.7, 146.3, 140.7, 140.5, 135.7, 132.0, 131.7, 131.0, 129.7, 128.5, 127.4, 124.9, 124.8, 124.5, 124.3, 124.2, 123.6, 123.0, 67.4, 29.4, 29.3, 29.2, 29.1, 28.5, 28.4, 26.8, 26.4, 26.2, 25.3, 24.4, 23.9, 23.8, 23.4, 23.2, 23.0, (two signals missing due to overlap with solvent signals, see assignment-table on the next page). IR (film) $\tilde{\nu}$ 3068, 2963, 2928, 2867, 2125, 1534, 1465, 1447, 1387, 1290, 1261, 1102, 953, 933, 801, 756, 697, 443 cm^{-1}. Anal. (%) calcd for $C_{81}H_{112}Cl_5N_6Ru_2$ C 62.80, H 7.29, Cl 11.43, N 5.42, Ru 13.04; found: C 61.57, H 7.39, Cl 11.07, N 5.29, Ru 12.61.

^{1}H NMR (400 MHz, 298 K, toluene-[D_8]) δ 9.27 (d, *J* = 1.9 Hz, 2H), 7.38 (t, *J* = 7.7 Hz, 1H), 7.28 (q, *J* = 6.2, 4.8 Hz, 5H), 7.17 (dd, *J* = 6.2, 2.6 Hz, 12H), 6.53 (bs, 4H), 6.24 (t, *J* = 1.8 Hz, 1H), 3.30 (ddt, *J* = 18.0, 13.4, 6.8 Hz, 4H), 3.15 (t, *J* = 6.9 Hz, 4H), 2.57 (p, *J* = 6.8 Hz, 2H), 2.34 (p, *J* = 6.8 Hz, 2H), 1.35 (d, *J* = 6.7 Hz, 8H), 1.26 (d, *J* = 6.5 Hz, 18H), 1.17 (d, *J* = 6.8 Hz, 8H), 1.09 (d, *J* = 6.8 Hz, 14H), 1.07 – 1.00 (m, 12H), 0.94 (dd, *J* = 9.1, 6.9 Hz, 12H), –11.26 (s, 4H).

^{1}H NMR (400 MHz, 298 K, CD_2Cl_2) δ 8.73 (s, 2H), 7.85 (s, 1H), 7.51 (t, *J* = 7.8 Hz, 2H), 7.39 – 7.28 (m, 9H), 7.20 (d, *J* = 7.7 Hz, 4H), 7.11 (s, 3H), 6.82 (s, 4H), 3.01 – 2.89 (m, 4H), 2.81 (p, *J* = 6.7 Hz, 4H), 2.43 (p, *J* = 6.6 Hz, 4H), 1.33 (d, *J* = 6.3 Hz, 14H), 1.05 (dd, *J* = 13.5, 6.6 Hz, 30H), 0.91 (dd, *J* = 7.1, 4.7 Hz, 28H), –11.86 (s, 4H). ^{13}C NMR (101 MHz, 298 K, CD_2Cl_2) δ 188.5, 148.3, 146.9, 146.0, 139.7, 131.7, 130.8, 128.8, 124.7, 123.6, 28.9, 28.5, 28.3, 26.3, 25.6, 23.6, 23.0, 23.0.

NMR data supports the following structure

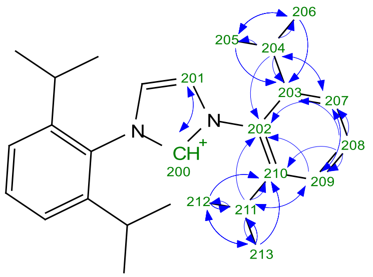

observed HMBC cross peaks

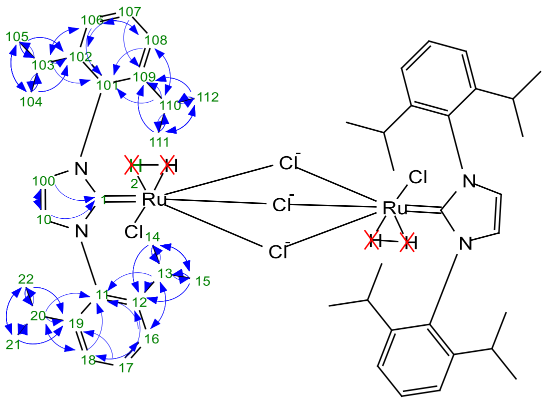

A few comments to the struture:
- Sample was measured at -40°C as at higher temperatures only broad signals were observed.
- At -40°C there are multiple EXSY peaks visible in the 2D ROESY, e.g. H100 <-> H10, H18<->H108, H16<->H106, but not between protons on the same ring (e.g. H18 and H16).
This can either be explained by a rotation around the C1-Ru-bond or more likely a flip on of the briging Cl-
- Intermolecular NOEs (eg. H201 <-> H105 and H-203<->16/106) show the close spacial proximity of the cation and the anion in solution
-Due to the exchange of the two sites in the cation, some NOEs might be explained by the chem. exchange as well (e.g. H14 sees H105). Therefore also the assignment of CH100 and CH 10 might be exchangable. Due to probe limitations on the cryoprobe the sample could not be cooled further.
- The sample might contain some minor amount of free NHC ligand

User Report
BIU-BB-709-21

Atom	δ (ppm)	COSY	HSQC	HMBC	NOESY
1 C	189.630			10, 100	
2 H	-11.544				
10 C	124.374		10	100	
H	7.140	100	10	1, 100	13, 14, 20, 21, 103, 104, 110, 111
11 C	140.718			13, 16, 18, 20	
12 C	147.504			13, 14, 15, 17	
13 C	29.136		13	14, 15, 16	
H	2.851	14, 15	13	11, 12, 14, 15, 16	10, 14, 15, 100, 104, 105
14 C	26.847		14	13, 15	
H3	1.010	13	14	12, 13, 15	10, 13, 16, 100, 103, 105, 106
15 C	23.412		15	13, 14	
H3	1.325	13	15	12, 13, 14	13, 16, 103, 106
16 C	124.233		16	13, 18	
H	7.323	17	16	11, 13, 18	14, 15, 104, 105, 213
17 C	129.790		17		
H	7.429	16, 18	17	12, 19	
18 C	124.523		18	16, 20	
H	7.275	17	18	11, 16, 20	21, 22, 112
19 C	148.963			17, 20, 21, 22	
20 C	28.553		20	18, 21, 22	
H	2.799	21, 22	20	11, 18, 19, 21, 22	10, 21, 22, 100, 112
21 C	26.240		21	20, 22	
H3	0.876	20	21	19, 20, 22	10, 18, 20
22 C	23.210		22	20, 21	
H3	1.079	20	22	19, 20, 21	18, 20, 108, 110

Atom	δ (ppm)	COSY	HSQC	HMBC	NOESY
100 C	127.424		100	10	
H	6.841	10	100	1, 10	13, 14, 20, 103, 104, 110, 111
101 C	140.566			103, 106, 108, 110	
102 C	146.717			103, 104, 105, 107	
103 C	28.396		103	104, 105, 106	
H	2.959	104, 105	103	101, 102, 104, 105, 106	10, 14, 15, 100, 104, 105
104 C	26.448		104	103, 105	
H3	0.818	103	104	102, 103, 105	10, 13, 16, 100, 103, 105, 106
105 C	23.954		105	103, 104	
H3	0.455	103	105	102, 103, 104	13, 14, 16, 103, 104, 106, 201
106 C	123.640		106	103, 108	
H	6.876	107	106	101, 103, 108	14, 15, 104, 105, 213
107 C	128.557		107		
H	7.096	106, 108	107	102, 109	
108 C	123.066		108	106, 110	
H	6.962	107	108	101, 106, 110	22, 111, 112
109 C	148.255			107, 110, 111, 112	
110 C	29.404		110	108, 111, 112	
H	3.310	111, 112	110	101, 108, 109, 111, 112	10, 22, 100, 111, 112
111 C*	25.470		111	110, 112	
H3	0.876	110	111	109, 110, 112	10, 100, 108, 110
112 C	23.845		112	110, 111	
H3	1.000	110	112	109, 110, 111	18, 20, 108, 110
200 C	135.745		200	201	
H	8.865	201	200	201	204, 205, 211, 212

Atom	δ (ppm)	COSY	HSQC	HMBC	NOESY
201 C	131.080		201	200, 201	
H	9.104	200	201	200, 201	105, 204, 206, 211
202 C	132.074			204, 207, 209, 211	
203 C	146.847			204, 205, 206, 208	
204 C	29.369		204	207	
H	2.611	205, 206	204	202, 203, 205, 206, 207	200, 201, 205, 206
205 C	26.240		205	204, 206	
H3	0.971	204	205	203, 206	200, 204, 207
206 C	22.997		206	204, 205	
H3	1.314	204	206	203, 205	201, 204, 207
207 C	124.916		207	204, 208, 209	
H	7.311	208	207	202, 204, 209	205, 206
208 C	131.783		208		
H	7.495	207, 209	208	203, 207, 209, 210	
209 C	124.840		209	207, 208, 211	
H	7.358	208	209	202, 207, 211	212, 213
210 C	146.348			208, 211, 212, 213	
211 C	29.290		211	209, 212, 213	
H	2.326	212, 213	211	202, 209, 210, 212, 213	200, 201, 212, 213
212 C*	25.530		212	211, 213	
H3	1.060	211	212	210, 211, 213	200, 209, 211
213 C	24.475		213	211, 212	
H3	1.243	211	213	210, 211, 212	16, 106, 209, 211

* 13C signals of C212 and C111 were extracted from the 2D HSQC

Deuterium-Labelled Ruthenate σ-HD complex [D_2]-9. In a flame-dried quartz Schlenk tube under argon,

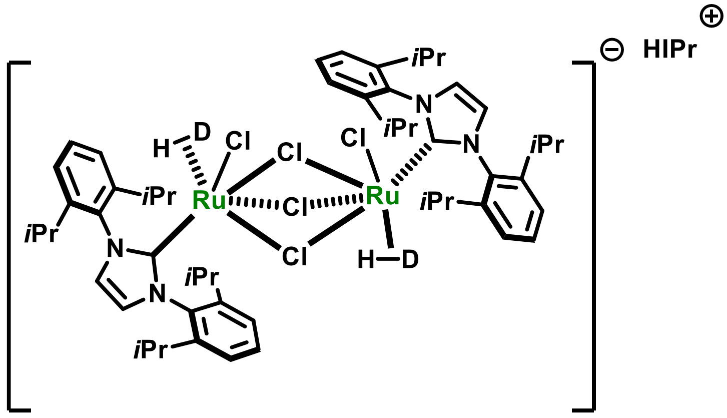

[(IPr)Ru(*p*-cymene)Cl_2] (**1b**)[1] (119 mg, 171 µmol) and IPrHCl (145 mg, 342 µmol) were suspended in toluene (15 mL). The Schlenk tube was closed with a septum and then transferred into the PhotoRedOxBox. A balloon filled with H–D gas was connected to a needle, which was pierced through the septum. The Schlenk tube was flushed with HD for 2 min through an outlet cannula (the cannula did not reach into the solution to ensure that only the head space of the tube was flushed). The exit cannula was removed but the balloon remained attached to the Schlenk tube to ensure constant pressure of HD gas throughout the reaction. The light source was switched on and the reaction mixture was stirred for 20 min under HD atmosphere. After this time, the yellow suspension was filtered via a filter canula into another Schlenk tube and the filtrate was concentrated in vacuum. The remaining material was washed with pentane (3 x 10 mL). Drying under high vacuum afforded the title compound (101 mg, 76%) as a yellow solid.

^{1}H NMR (400 MHz, CD_2Cl_2) δ 8.53 (s, 2H), 7.91 (s, 1H), 7.52 (t, *J* = 7.8 Hz, 2H), 7.37 – 7.29 (m, 9H), 7.20 (d, *J* = 7.8 Hz, 4H), 7.16 – 7.08 (m, 3H), 6.81 (s, 4H), 2.96 – 2.88 (m, 4H), 2.88 – 2.77 (m, 4H), 2.53 – 2.39 (m, 4H), 1.35 (d, *J* = 6.2 Hz, 14H), 1.05 (dd, *J* = 9.8, 6.5 Hz, 30H), 0.91 (dd, *J* = 7.4, 4.3 Hz, 28H), –11.91 (t, *J* = 29.4 Hz, 2H). ^{13}C NMR (101 MHz, CD_2Cl_2) δ 188.6, 148.3, 146.9, 146.0, 139.7, 131.7, 130.8, 128.8, 124.7, 123.6, 28.9, 28.5, 28.3, 26.3, 25.6, 23.6, 23.0, 23.0.

As can be seen in Figure S12, the 1:1:1 triplet in the hydride region of this complex is not perfectly symmetrical because of overlapping signal of undeuterated **9**. From the recorded $^1J_{HD}$ = 29.4 Hz of [D_2]-**9** one can deduced the following H–H distances ($d_{H,H}$) for the ligated H_2 molecule:

$d_{HH} = 1.42 - 0.0167 J_{HD}$ (Morris et al.)[2]

$d_{HH} = 1.44 - 0.0168 J_{HD}$ (Heinekey et al.)[3]

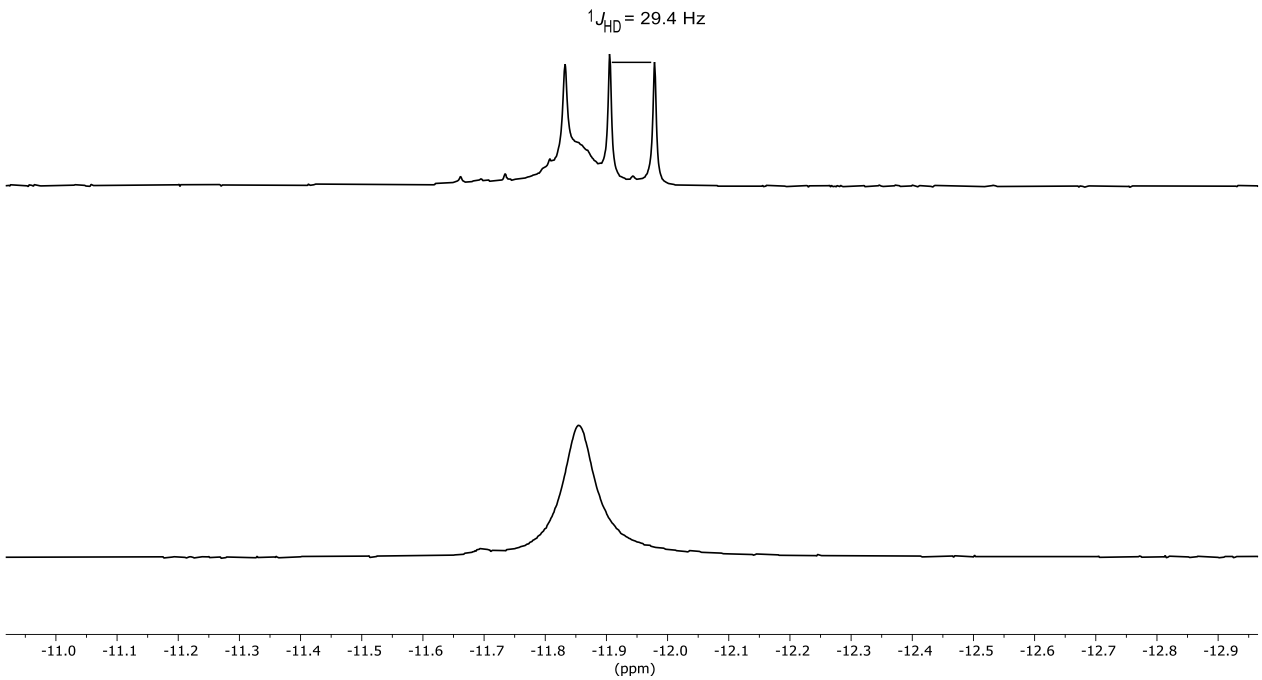

Figure S12. Hydride region of the ^{1}H NMR spectra (CD_2Cl_2) of [D_2]-**9** (top) and unlabeled **9** (bottom).

Complex 8. In a flame-dried Schlenk tube under argon, [(IPr)(*p*-cymene)$RuCl_2$] (**1b**)[1] (100 mg, 143 µmol) was dissolved in toluene (15 mL). The Schlenk tube was closed with a septum and a hydrogen-filled balloon was connected to a needle, which was pierced through the septum. The Schlenk tube was flushed with hydrogen for 2 min through an outlet cannula (the cannula did not reach into the solution to ensure that only the head space of the tube was flushed). The exit cannula was removed but the hydrogen-filled balloon remained attached to the Schlenk tube to ensure constant hydrogen pressure throughout the reaction. The mixture was heated to 90 °C for 1 h under a hydrogen atmosphere in the dark. After this time, the red solution was evaporated *in vacuo*. The remaining material was suspended in pentane and filtered with a filter canula into another Schlenk flask. The filtrate was concentrated in vacuum to provide the title compound as a red crystalline solid (34 mg, 42%). Red

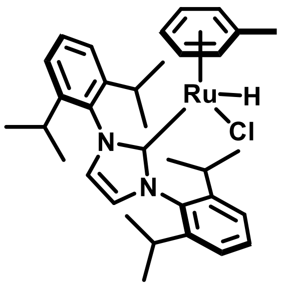

crystals suitable for X-ray diffraction were grown upon slowly cooling a saturated pentane solution from room temperature to –20 °C.

^{1}H NMR (600 MHz, toluene-[D_8]) δ 7.29 (t, *J* = 7.7 Hz, 2H), 7.21 (d, *J* = 7.8 Hz, 2H), 7.14 (dd, *J* = 7.7, 1.5 Hz, 2H), 6.61 (s, 2H), 4.83 (t, *J* = 5.5 Hz, 1H), 4.64 (d, *J* = 5.8 Hz, 1H), 4.35 (d, *J* = 5.5 Hz, 1H), 4.15 (t, *J* = 5.5 Hz, 1H), 3.63 (td, *J* = 5.1, 0.8 Hz, 1H), 3.34 (hept, *J* = 7.0 Hz, 2H), 2.93 (hept, *J* = 7.0 Hz, 2H), 1.75 (s, 3H), 1.34 (d, *J* = 6.8 Hz, 7H), 1.33 (d, *J* = 6.7 Hz, 6H), 1.05 (d, *J* = 6.9 Hz, 6H), 0.98 (d, *J* = 6.9 Hz, 6H), –6.06 (s, 1H). ^{13}C NMR (151 MHz, toluene-[D_8]) δ 190.0, 146.8, 146.7, 139.0, 123.4, 123.3, 106.9, 93.4, 86.1, 82.6, 79.8, 66.7, 28.4, 28.1, 26.0, 25.5, 22.8, 22.0, 18.6.

NMR data supports the following structure:

User Report
BIU-BB-661-01

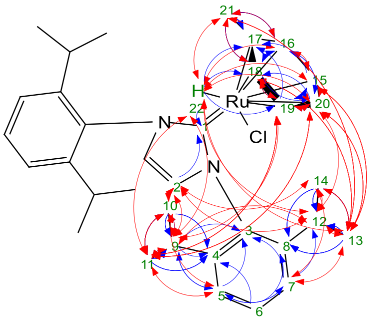

All the observed NOE cross correlation peaks and multiple HMBC agree with the given structure. Remarkably, the hydride H22 shows HMBC to the tolyl ligand and C1 and NOE crosspeaks to the iPr groups. The 13C shifts of the toluene are all below 100 ppm indicating their η^6 coordination to the Ru.

VT measurements show broadening if the iPr groups at lower temperature

The structure could be additionally confirmed in the solid state by X-Ray crystallography.

P-ID: ML00000
Measured on: 21/05/2020
CHIFFRE:BIU-BB-661-01
ELNA#:3994
Client: Tobias Biberger
Group: Fürstner
Spectroscopist: Leutzsch
Analysed on: 03/06/2020
Analysed by: Leutzsch
Amount: 15.0 mg
Solvent: Tol
Reference: solvent
Temperature: 298 K
Spectrometer: av600neo
Probe: cryBBO
Experiments: 1H-zg30, 13C-zgpg30, [13C, 1H]-hsqcedetgpsisp2.4, [13C, 1H]-hmbcetgpl3nd, [13C, 1H]-hmbcetgpl3nd, [1H, 1H]-clipcosy, 1H-zg30, 1H-t1ir, [1H, 1H]-noesygpph, 1H-zg30

Atom	δ (ppm)	J	COSY	HSQC	HMBC	NOESY
1 C	190.07			1'	2, 22	
2 C	123.42			2	2	
H	6.61			2	1, 2	9, 10, 12, 14
3 C	139.02				5, 7, 9	
4 C	146.85				6, 9, 10, 11	
5 C	123.68			5	7, 9	
H	7.14	1.50(7), 7.70(6)	6, 7	5	3, 7, 9	10, 11
6 C	129.35			6		
H	7.29	7.70(5), 7.80(7)	5, 7	6	4, 8	
7 C	123.35			7	5	
H	7.21	1.50(5), 7.80(6)	5, 6	7	3, 5, 12	13, 14
8 C	146.77				6, 13, 14	
9 C	28.13			9	5, 10, 11	
H	2.93	6.90(10), 6.80(11)	10, 11	9	3, 4, 5, 10, 11	2, 10, 11, 12, 19, 22
10 C	26.07			10	9, 11	
H3	1.05	6.90(9)	9	10	4, 9, 11	2, 5, 9, 11
11 C	22.88			11	9, 10	
H3	1.34	6.80(9)	9	11	4, 9, 10	5, 9, 10, 19, 20, 22
12 C	28.43			12	7, 13, 14	
H	3.34	7.00(14), 6.70(13)	13, 14	12		2, 9, 13, 14, 20, 22

Atom	δ (ppm)	J	COSY	HSQC	HMBC	NOESY
13 C	22.05			13	14	
H3	1.33	6.70(12)	12	13	8, 12, 14	7, 12, 14, 15, 18, 20, 21, 22
14 C	25.35			14	13	
H3	0.98	7.00(12)	12	14	8, 12, 13	2, 7, 12, 13, 14
15 C	79.82			15	19, 20	
H	4.83	5.50(16)	16, 20	15	17, 19, 20	13, 16, 20, 22
16 C	82.61			16	18, 20, 21	
H	4.35	5.50(15)	15	16	18, 20, 21	15, 21, 22
17 C	106.93				15, 19, 21, 22	
18 C	93.48			18	16, 20, 21, 22	
H	4.64	5.50(19)	19	18	16, 19, 20	13, 19, 21
19 C	86.12			19	15, 18, 20, 22	
H	4.15	5.50(18), 5.50(20)	18, 20	19	15, 17, 20	9, 11, 18, 20
20 C	66.73			20	15, 16, 18, 19	
H	3.63	5.50(19)	15, 19	20	15, 16, 18, 19	11, 12, 13, 15, 19, 22
21 C	18.66			21	16	
H3	1.75			21	16, 17, 18	13, 16, 18, 22
22 H	-6.06				1, 17, 18, 19	9, 11, 12, 13, 15, 16, 20, 21

Kinetic Profiling. The catalytic performance of complexes **8** and **9** was evaluated by means of the standard hydrogenative metathesis reaction of enyne **6**. In case of complex **8**, presence of cyclopentene **7** in the resulting complex crude reaction mixture could not be confirmed.

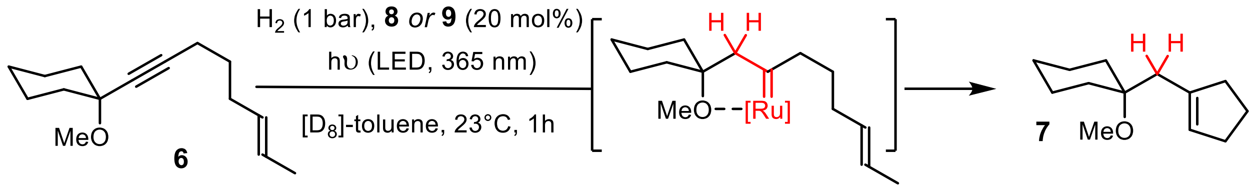

A flame-dried quartz Schlenk tube was charged with **9** (77.6 mg, 0.05 mmol, 20 mol%) [or **1b** (69.6 mg, 0.1 mmol, 20 mol%)], enyne **6** (110 mg, 0.5 mmol) and toluene (5 mL). The Schlenk tube was closed with a septum and then transferred into the PhotoRedOxBox TC, cooled to approx. 23 °C. A hydrogen-filled balloon was connected to a needle, which was pierced through the septum. The Schlenk tube was flushed with hydrogen for 2 min through an outlet cannula (the cannula did not reach into the solution to ensure that only the head space of the tube was flushed). The exit cannula was removed but the hydrogen-filled balloon remained attached to the Schlenk tube to ensure constant hydrogen pressure throughout the reaction. The light source was switched on and samples were taken manually with oven-dried syringes every 2 mins; the samples were filtered through a short pad of silica and the filtrates analyzed by GC-MS.

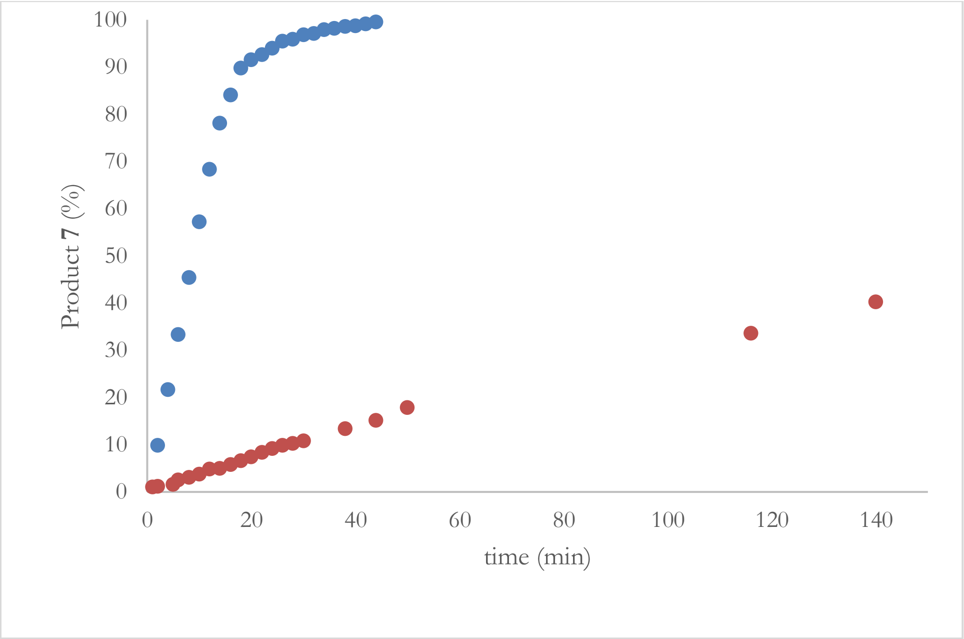

Figure S13. Formation of product **7** by light-driven hydrogenative metathesis of enyne **6** catalyzed by complex **1b** (blue) or complex **9** (red)

T_1(min) Measurements. The temperature-dependent T_1 relaxation times of complex **9** were measured on four different instruments (Bruker AvanceIII-300 MHz WB, Bruker AvanceIIIHD-400 MHz, Bruker AvanceIII-500 MHz, Bruker AvanceNeo 600 MHz NMR spectrometers). Temperatures for the variable temperature (VT) experiments from –60 to RT (25°C) were calibrated with 4% MeOH in [D_4]-MeOH using the implemented "calctemp" function in Bruker Topspin 3.6.1 or Topspin 4.1.2 (AVneo).[4] Temperatures above RT were calibrated with an 80% glycol in [D_6]-DMSO.[5]

The spin-lattice relaxation times (T_1) were determined with the inversion recovery sequence (Bruker pulse program: t1ir) using 32 non-linear spaced inversion times ranging from 0.001 ms to approx. 5 * T_1, which were adjusted individually to the different field strengths. The pulse offset during the measurement was set on-resonance on the hydride signal (≈ −11 ppm). The obtained 2D datasets were analyzed with the Bruker TOPSPIN T1T2 module by integrating the hydride signal and fitting it to the following equation:

$$I(\tau) = I_0 + P * e^{(\frac{-\tau}{T_1})}$$

I_0 describes the magnetization at thermal equilibrium, τ is the individual inversion time at each step in the 2D sequence, P is the polarization at t = 0.

Morris et al. developed equations that can be used to calculate an upper and lower limit of d_{HH}, depending on whether the H_2 ligand is spinning slower or faster than the spectrometer frequency:

$$d_{HH}^{slow} = 5.81\sqrt[6]{\frac{T_{1(min)}}{v}} \qquad d_{HH}^{fast} = 4.61\sqrt[6]{\frac{T_{1(min)}}{v}}$$

ν=spectrometer frequency in MHz

Application of the T_1(min) method to complex **9** finds a value of 11.773 ms (300 MHz). This value corresponds to a d_{HH} = 0.85 – 1.06 Å, depending on the rotational regime of the H_2 ligand. When the lower (0.85 Å) and the upper (1.06 Å) limit of d_{HH} are compared with the value obtained by the $^1J_{HD}$ method (0.94 Å) it can be deduced that the H_2 ligand is neither in the slow spinning nor in the fast spinning regime (see below).

More recently, Morris et al. have put forward a criterion to classify a H_2 complex into the slow or fast spinning regime.[6]

$$\frac{d_{HH}^{from\ J(HD)}}{581.5\sqrt[6]{\frac{T_{1(min)}}{v}}} = 1.0\ (if\ slow)\ or\ 0.794\ (if\ fast)$$

Applying this equation to the obtained data of **9**, a value of 0.89 is obtained. According to Morris, d_{HH} of complexes that fall *between* the values of 1.0 and 0.794 can be calculated by taking the average of d_{HH}^{slow} and d_{HH}^{fast}.[6] The average value of 0.96 Å is in excellent agreement with the value of 0.94 Å obtained by the $^{1}J_{HD}$ method.

Examination of the ln(T_1)/T^{-1} plot provides further evidence for the proposed rotational regime of **9**: ln(T_1)/T^{-1} plots for slow or fast spinning H_2 complexes are usually V-shaped with a sharp minimum. However, the shown plot exhibits an unusually *broad* minimum. Complexes with such distorted ln(T_1)/T^{-1} plots are suggestive of H_2 ligands that have a frequency of motion near the ^{1}H Larmor frequency and hence d_{HH} is between the values calculated from the T_1(min) value for fast spinning and slow spinning extremes.

Raw data:

T (K)	1/T	300 MHz				400 MHz			500 MHz			600 MHz		
		T_1 (ms)	R_1 (s^{-1})	ln (T_1)	LW	T_1 (ms)	R_1 (s^{-1})	ln (T_1)	T_1 (ms)	R_1 (s^{-1})	ln (T_1)	T_1 (ms)	R_1 (s^{-1})	ln (T_1)
213	4.69E-03	33.926	29.476	-3.384	160.91	53.347	18.745	-2.931	76.596	13.056	-2.569			
218	4.59E-03	27.902	35.840	-3.579	145.77	44.668	22.387	-3.108	64.854	15.419	-2.736			
223	4.48E-03	22.77	43.917	-3.782	124.27	38.803	25.771	-3.249	55.455	18.033	-2.892			
228	4.39E-03	19.72	50.710	-3.926	111.94	34.01	29.403	-3.381	48.516	20.612	-3.026	68.831	14.528	-2.676
233	4.29E-03	17.499	57.146	-4.046	101.57	29.974	33.362	-3.507	42.85	23.337	-3.150	60.402	16.556	-2.807
238	4.20E-03	16.546	60.438	-4.102	92	26.706	37.445	-3.623	38.38	26.055	-3.260	53.907	18.550	-2.920
243	4.12E-03	15.113	66.168	-4.192	83.76	23.993	41.679	-3.730	34.53	28.960	-3.366	48.283	20.711	-3.031
248	4.03E-03	14.137	70.736	-4.259		21.88	45.704	-3.822	31.133	32.120	-3.469	43.655	22.907	-3.131
253	3.95E-03	13.13	76.161	-4.333	73.91	20.211	49.478	-3.902	28.645	34.910	-3.553	39.8	25.126	-3.224
258	3.88E-03	12.696	78.765	-4.366		18.994	52.648	-3.964	26.471	37.777	-3.632	36.618	27.309	-3.307
263	3.80E-03	12.224	81.806	-4.404	62.82	17.971	55.645	-4.019	24.784	40.349	-3.698	34.013	29.401	-3.381
268	3.73E-03	11.96	83.612	-4.426		17.182	58.200	-4.064	23.436	42.669	-3.753	31.777	31.469	-3.449
273	3.66E-03	11.887	84.126	-4.432	56.03	16.589	60.281	-4.099	22.79	43.879	-3.781	29.992	33.342	-3.507
278	3.60E-03	11.773	84.940	-4.442	52.27	16.121	62.031	-4.128	21.55	46.404	-3.837	28.811	34.709	-3.547
283	3.53E-03	11.902	84.019	-4.431		15.931	62.771	-4.139	20.775	48.135	-3.874	27.509	36.352	-3.593
288	3.47E-03	12.052	82.974	-4.419	46.06	15.861	63.048	-4.144	20.353	49.133	-3.895	26.584	37.617	-3.627
293	3.41E-03	12.239	81.706	-4.403		15.846	63.107	-4.145	20.093	49.769	-3.907	25.674	38.950	-3.662
298	3.36E-03	12.68	78.864	-4.368	39.97	15.928	62.783	-4.140	19.865	50.340	-3.919	25.36	39.432	-3.675
303	3.30E-03	13.104	76.313	-4.335		15.979	62.582	-4.136	19.825	50.441	-3.921	24.882	40.190	-3.694
308	3.25E-03	13.579	73.643	-4.299	34.82	16.265	61.482	-4.119	20.057	49.858	-3.909	24.597	40.655	-3.705
313	3.19E-03	14.191	70.467	-4.255		16.531	60.492	-4.103	20.251	49.3803	-3.900	24.497	40.8213	-3.709
318	3.14E-03	14.726	67.907	-4.218	31.27	16.844	59.368	-4.084	20.596	48.5531	-3.883	24.521	40.7814	-3.708

B_0 (T)	v_0 (MHz)	$T_{1, min}$ (ms)	d^{slow}	d^{fast}	d^{lib}	dHD
7.05	300.13	11.77	1.07	0.85	0.94	0.94
9.39	400.13	15.85	1.07	0.85	0.94	
11.73	499.87	19.83	1.07	0.85	0.94	
14.09	600.20	24.50	1.08	0.86	0.94	

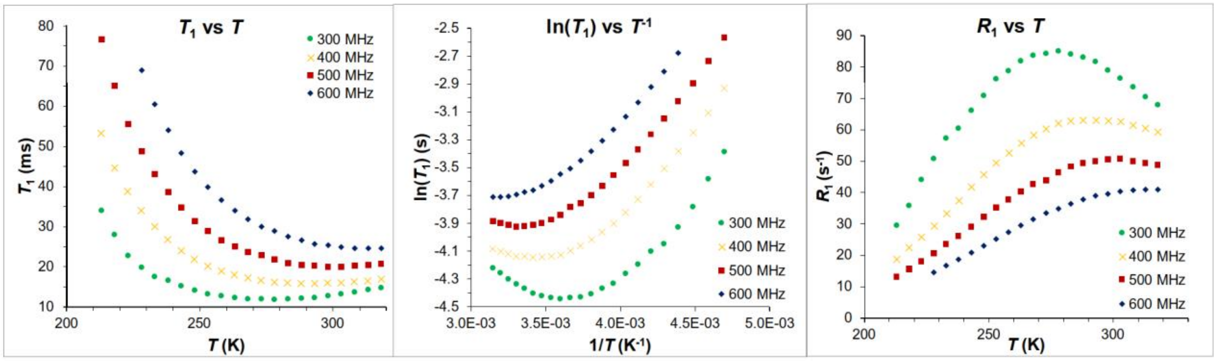

Solid State NMR

An attempt was made to determine the distance between the two hydrogen atoms via static solid-state NMR spectroscopy. In principle, the internuclear distance can be calculated directly from the dipolar coupling, which can be obtained from the Pake doublet line shape, as has been pointed out by Zilm et al.[7] Unfortunately, our attempts to detect the Pake pattern in static measurements of samples of **9** at 600 MHz between 100 K and room temperature were unsuccessful. In fact, even under MAS conditions (10 kHz) we were not able to detect a signal of the H_2 ligand, owing to the broad lines of the proton signals in the solid state.

DFT Calculations

The geometry of complex **9** was optimized with the Gaussian 09 program, using the PBE0 functional.[8] Ru was represented by the quasi-relativistic effective core from the Stuttgart group and the associated basis sets.[9,10,11] The remaining atoms (H, C, N, and Cl) were represented by a double-ζ Def2-SVP basis set.[12] A pruned (99,590) grid was used for the geometry optimization.

The SCF density was calculated with ADF 2014,[13] using the PBE0 functional and a Slater-type basis set of double-ζ quality (DZ). The density was visualized with the ADFView program.

^{1}H NMR of Complex 9, 600 MHz, THF-[D$_8$], −40 °C

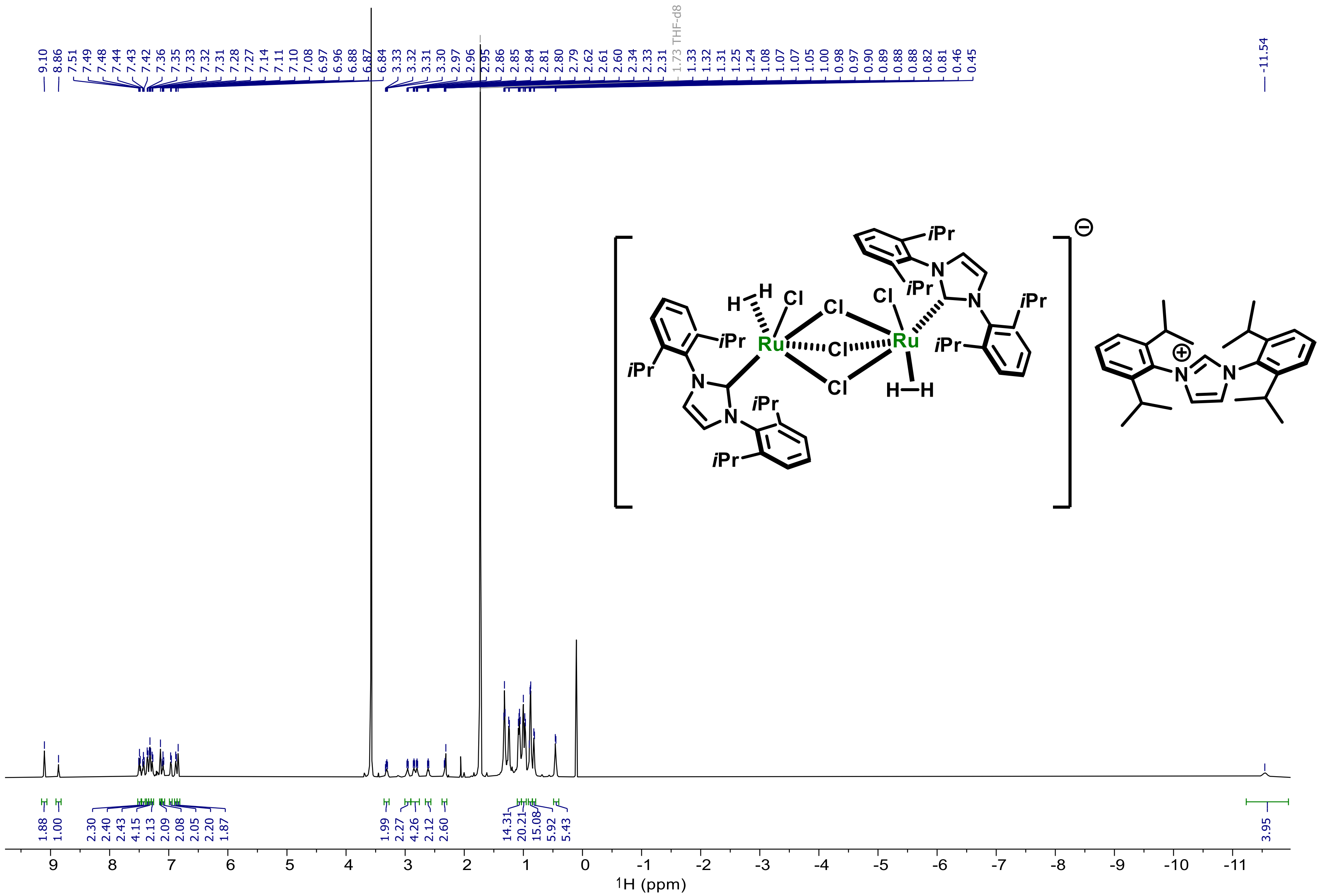

1H NMR spectra measured at different temperatures

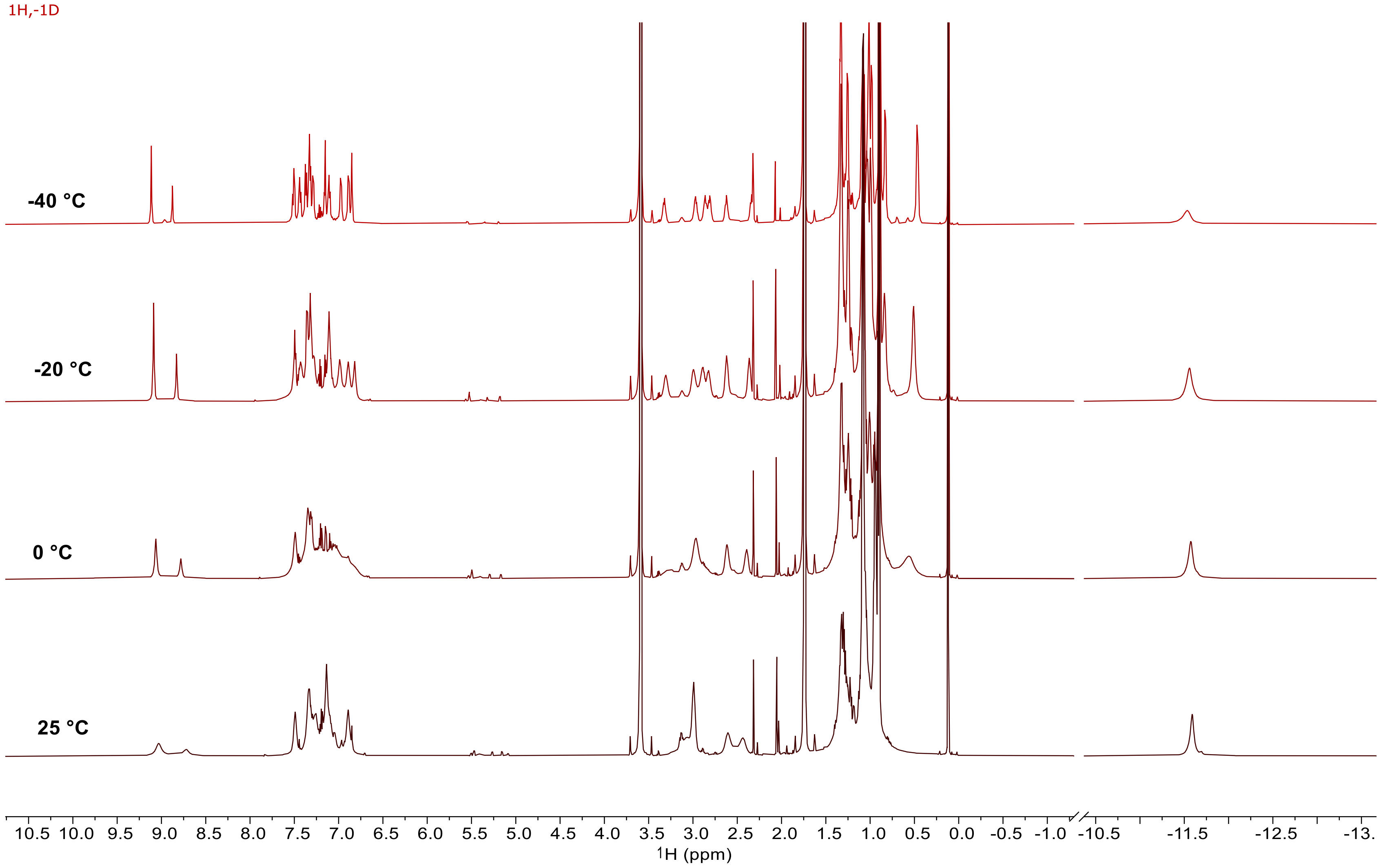

^{13}C NMR of Complex 9, 151 MHz, THF-[D$_8$], −40 °C

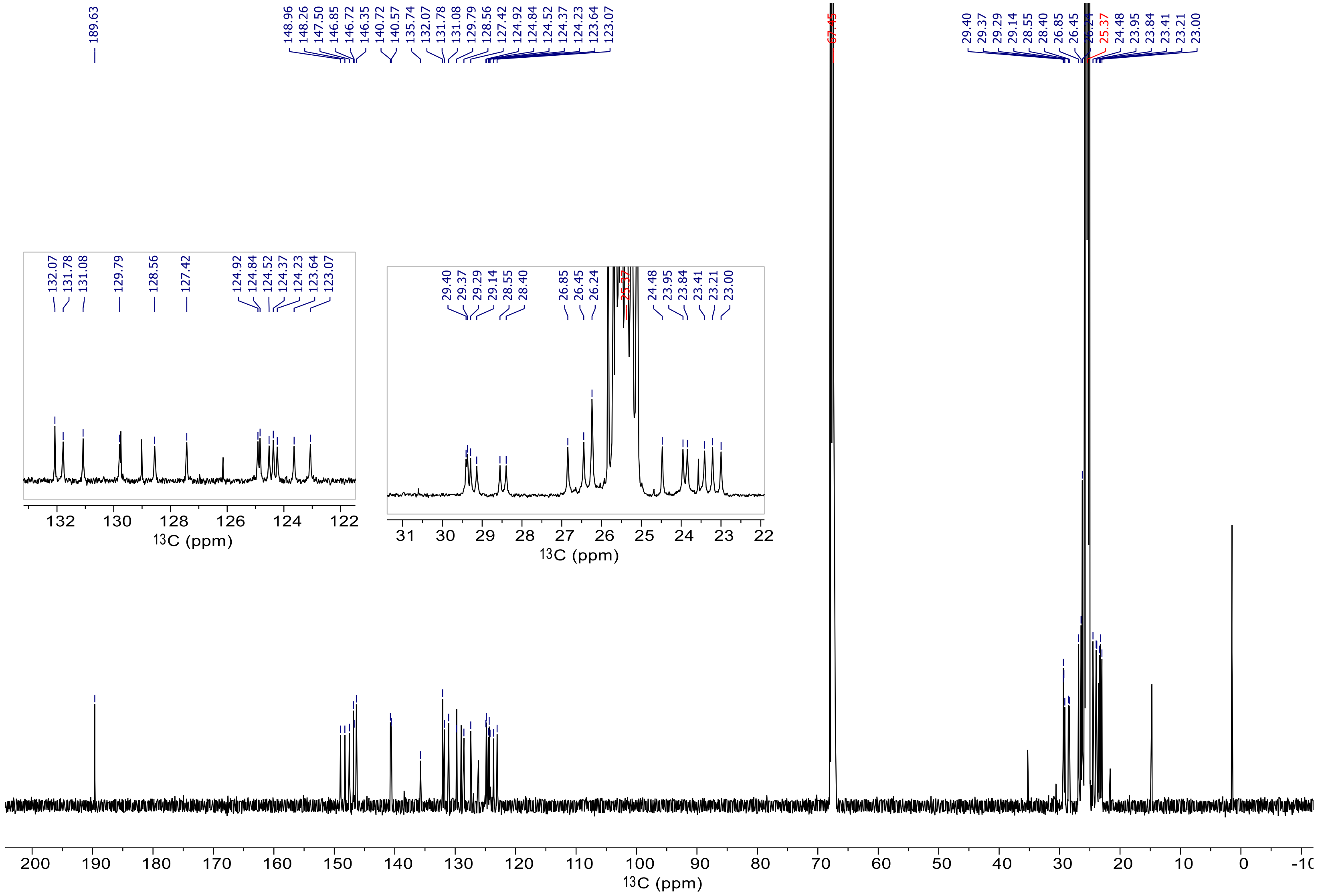

1H,13C-HSQC-EDITED

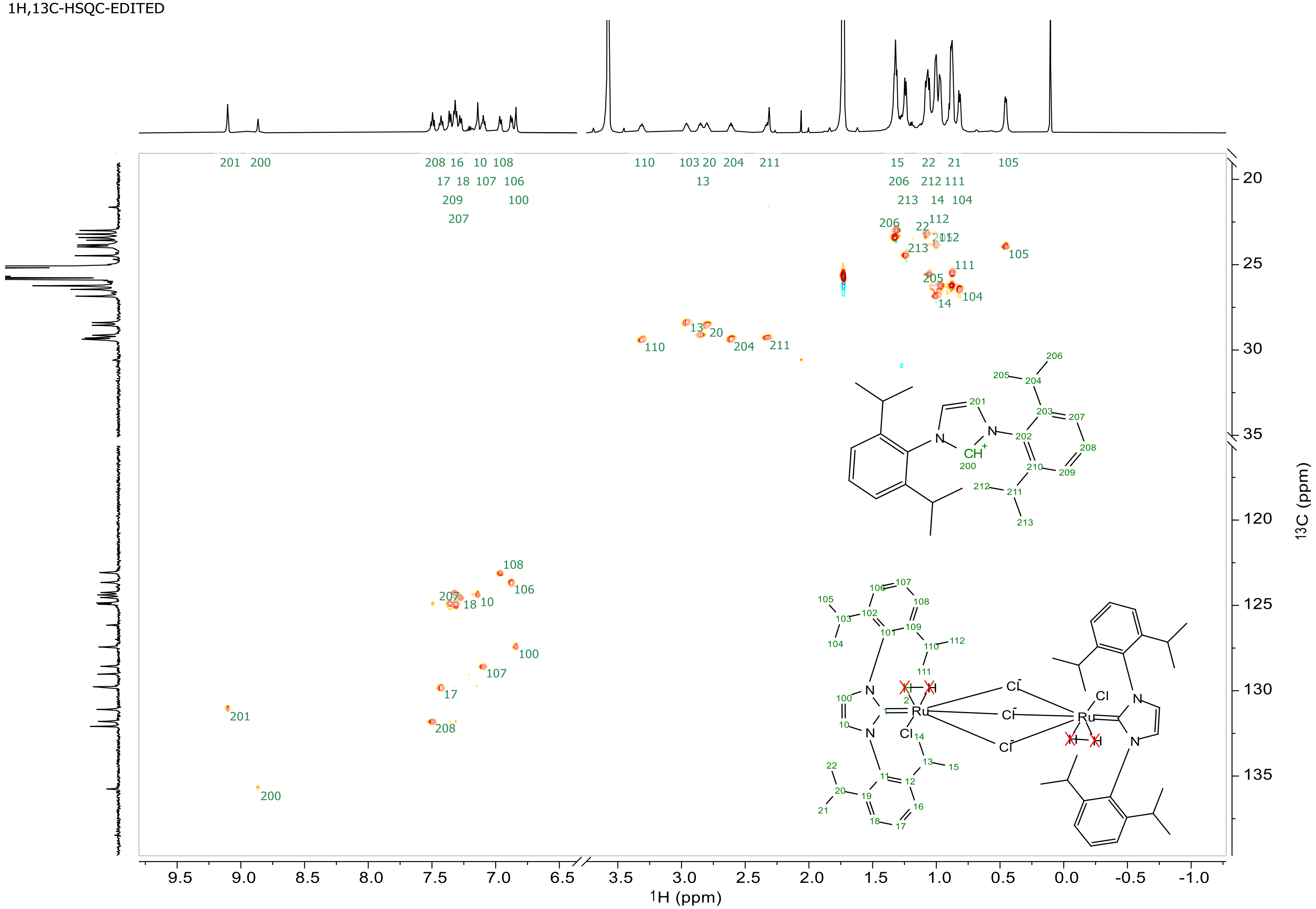

1H,13C-HSQC-EDITED

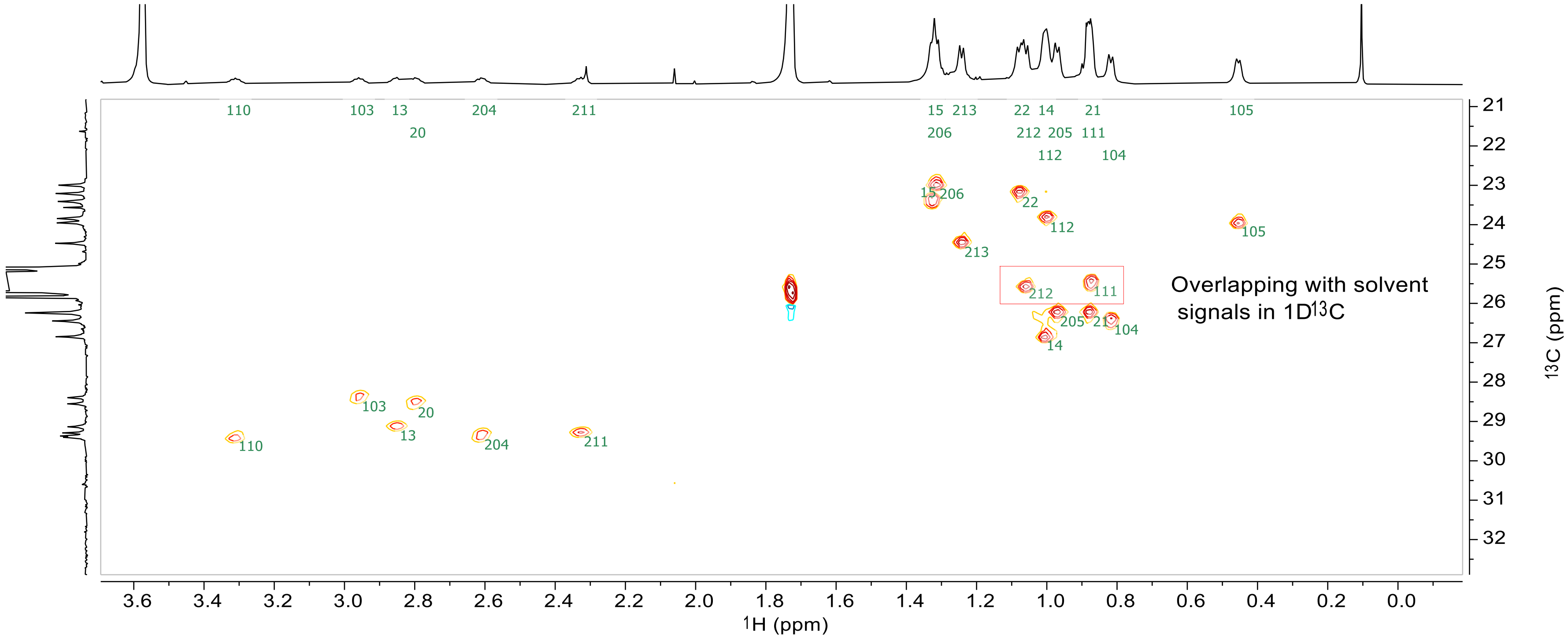

1H,13C-HSQC-EDITED

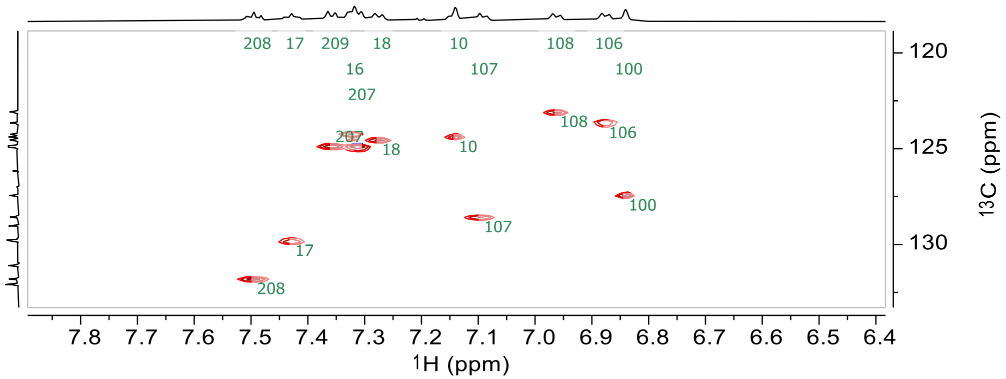

1H,13C-HMBC

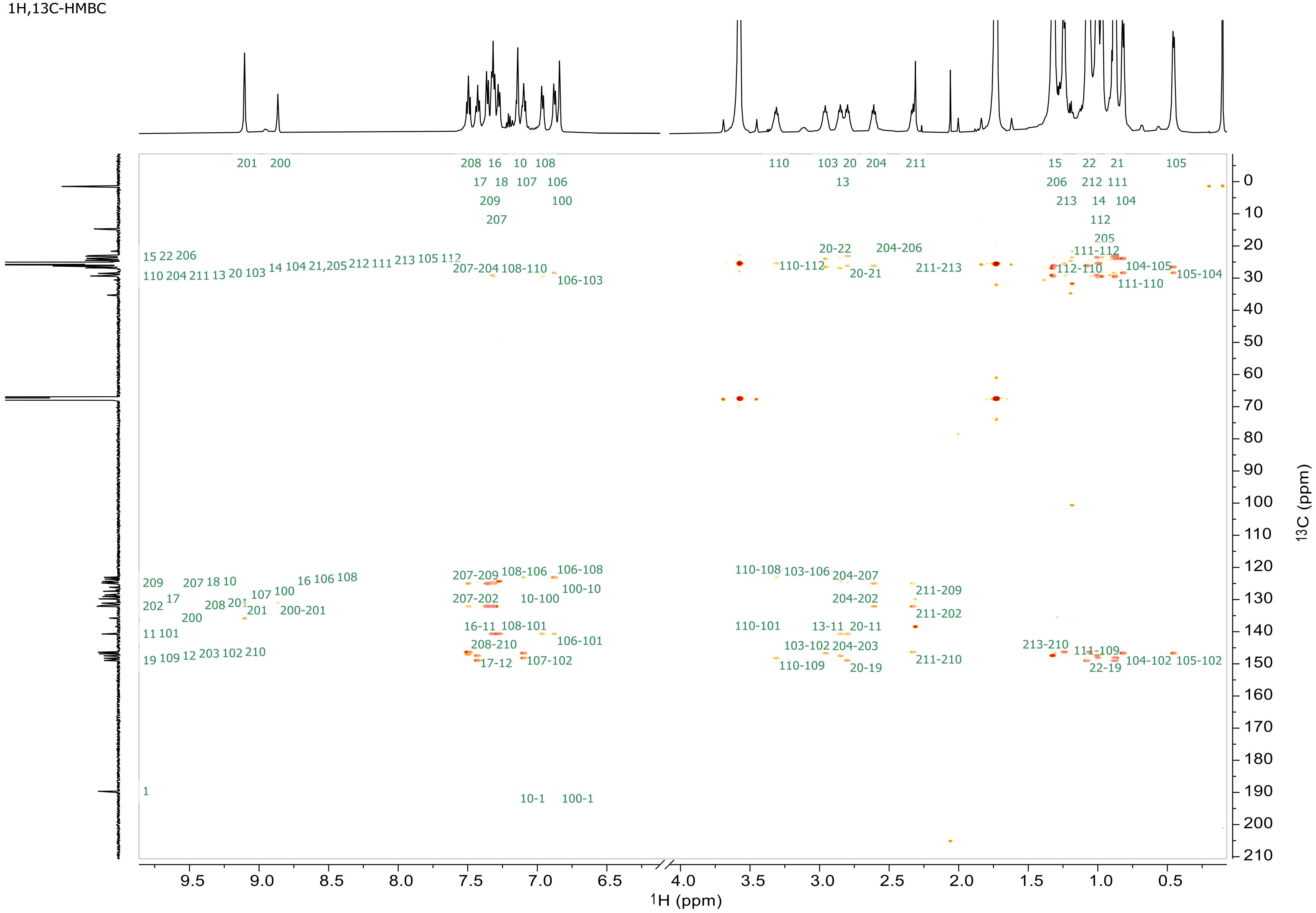

201 200 208 16 10 108 110 103 20 204 211 15 22 21 105
17 18 107 106 13 206 212 111
209 100 213 14 104
207 112
205
15 22 206
110 204 211 13 20 103 14 104 21,205 212 111 213 105 112
207-204 108-110 106-103
20-22 204-206 111-112
110-112 20-21 211-213 112-110 104-105 105-104 111-110
209 207 18 10 16 106 108
202 17 208 201 107 100 201 200-201
200
11 101
19 109 12 203 102 210
1
207-209 108-106 106-108 100-10
207-202 10-100
16-11 108-101 106-101
208-210 17-12 107-102
10-1 100-1
110-108 103-106 204-207 211-209
204-202 211-202
110-101 13-11 20-11
103-102 204-203 110-109 20-19 211-210
213-210 111-109 22-19 104-102 105-102
1H (ppm)
13C (ppm)

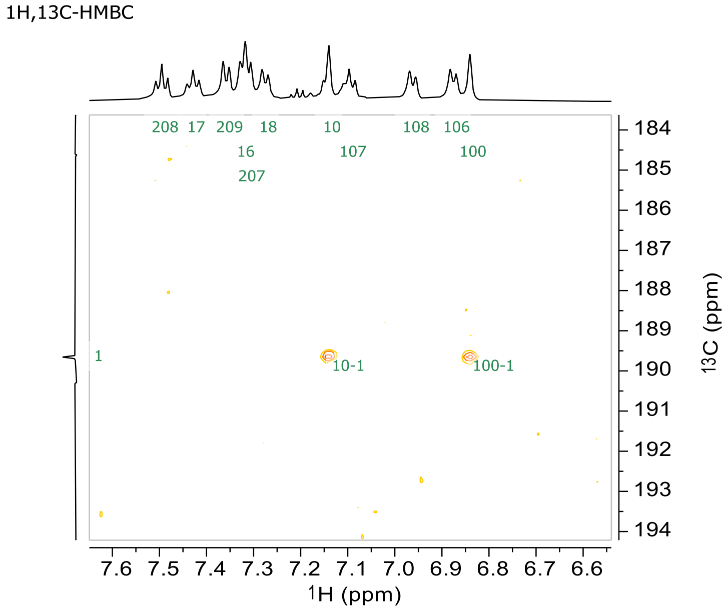
1H,13C-HMBC
208 17 209 18 10 108 106
16 107 100
207
1
10-1
100-1
1H (ppm)
13C (ppm)

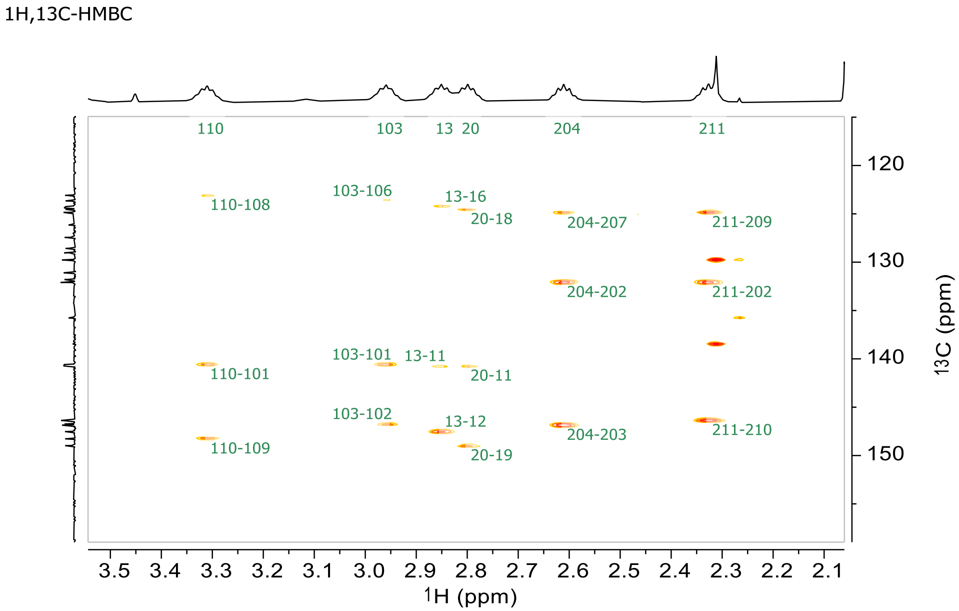
1H,13C-HMBC
110 103 13 20 204 211
110-108
103-106
13-16
20-18
204-207
211-209
204-202
211-202
103-101
13-11
110-101
20-11
103-102
13-12
204-203
211-210
110-109
20-19
1H (ppm)
13C (ppm)

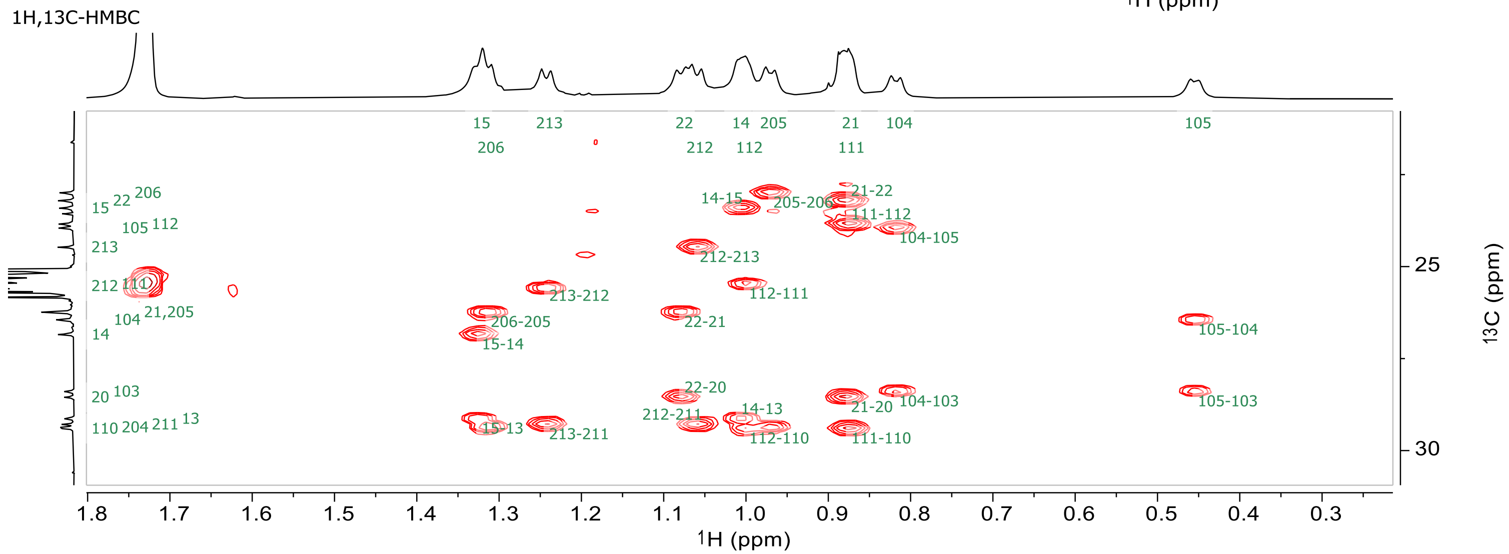
1H,13C-HMBC
15 213 22 14 205 21 104 105
206 212 112 111
15 22 206
105 112
213
212 111
104 21,205
14
20 103
110 204 211 13
14-15
205-206
21-22
111-112
104-105
212-213
213-212
112-111
206-205
22-21
105-104
15-14
22-20
104-103
105-103
21-20
212-211
14-13
15-13
213-211
112-110
111-110
1H (ppm)
13C (ppm)

1H,1H-ROESY

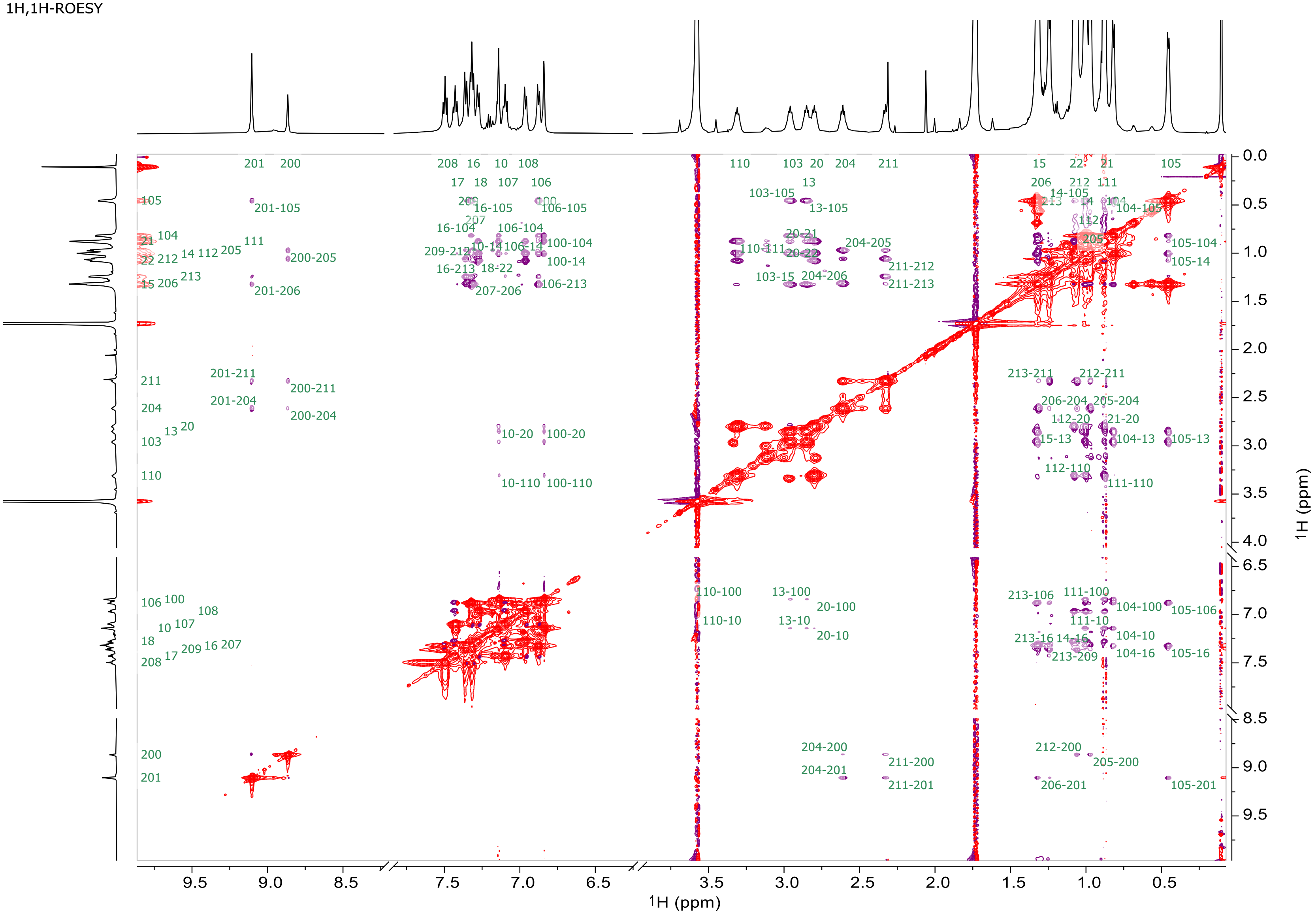

1H (ppm)
9.5 9.0 8.5 7.5 7.0 6.5 3.5 3.0 2.5 2.0 1.5 1.0 0.5
1H (ppm)
0.0 0.5 1.0 1.5 2.0 2.5 3.0 3.5 4.0 6.5 7.0 7.5 8.5 9.0 9.5

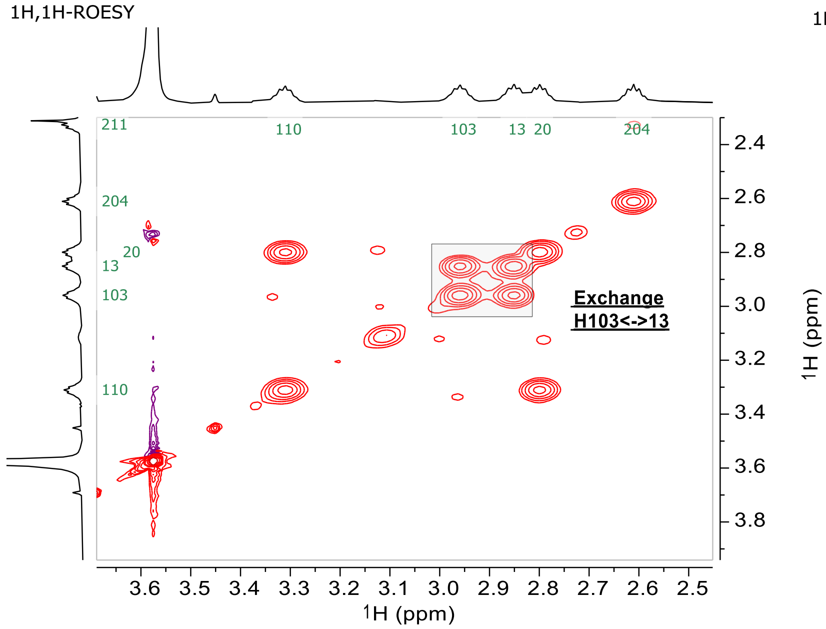

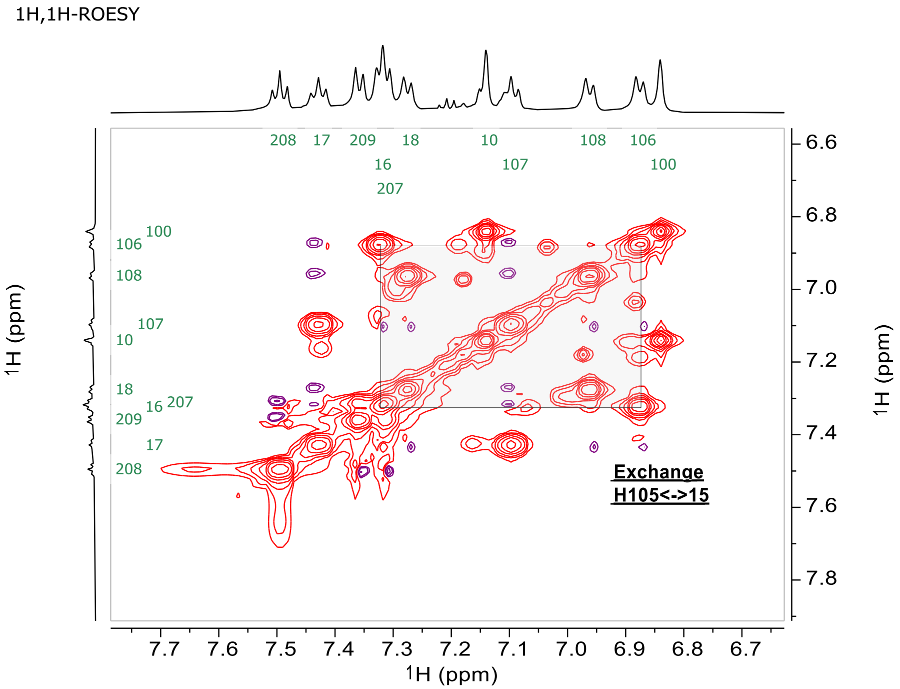

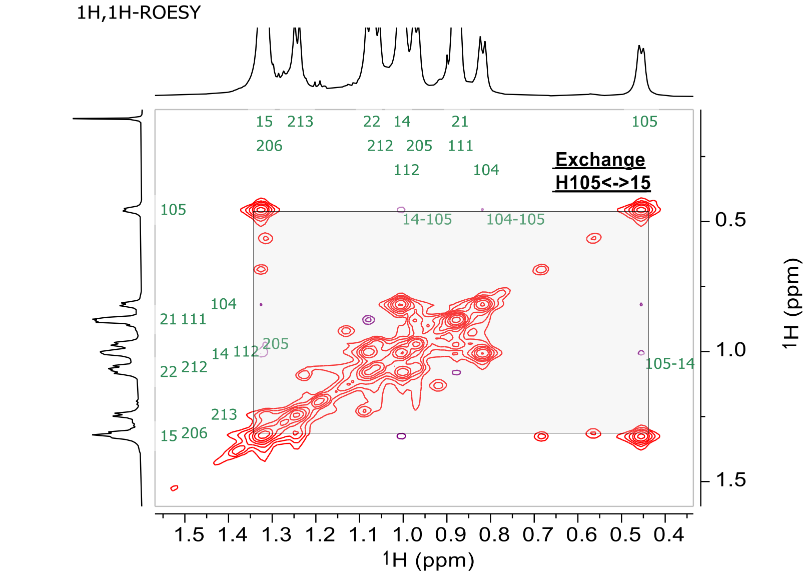

Regions showing EXSY peaks (red cross peaks)

1H,1H-ROESY

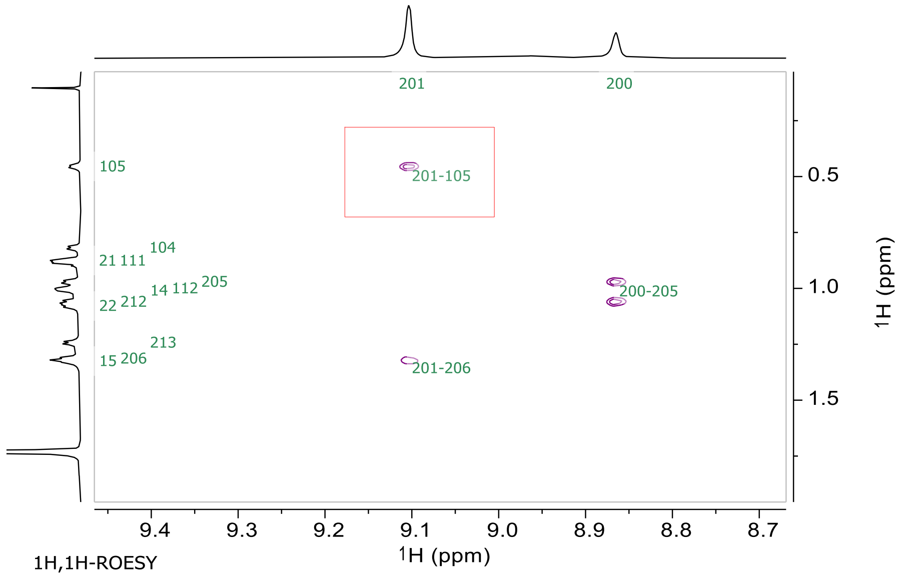

1H,1H-ROESY

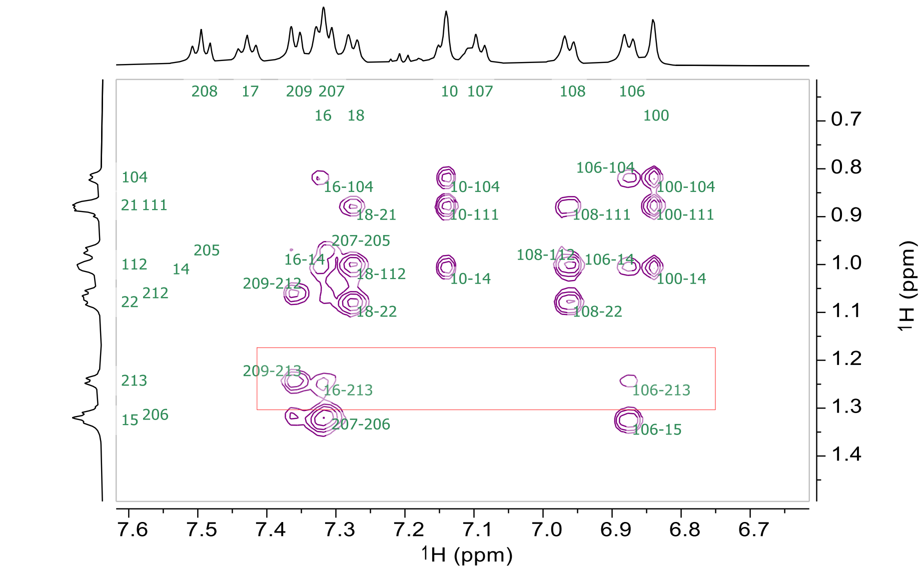

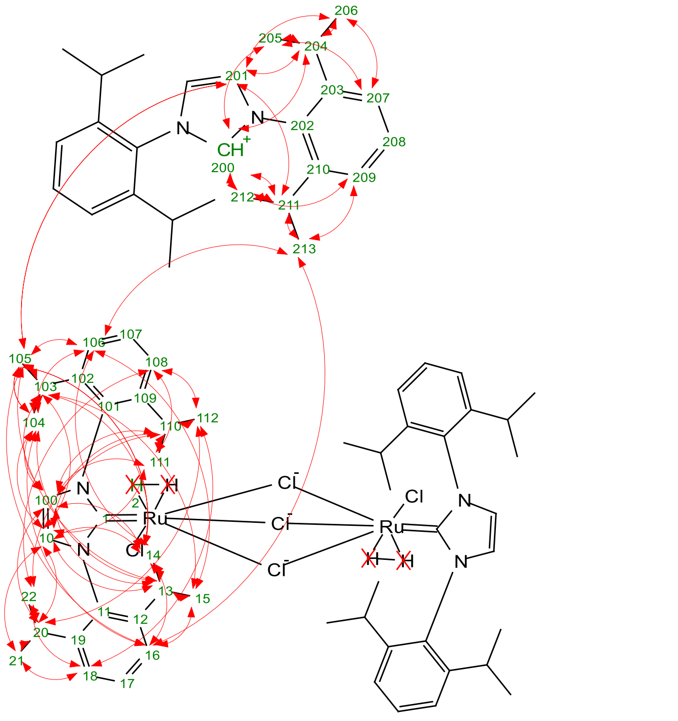

NOEs between the cation and anion are indicating their close spacial proximity

1H,1H-COSY

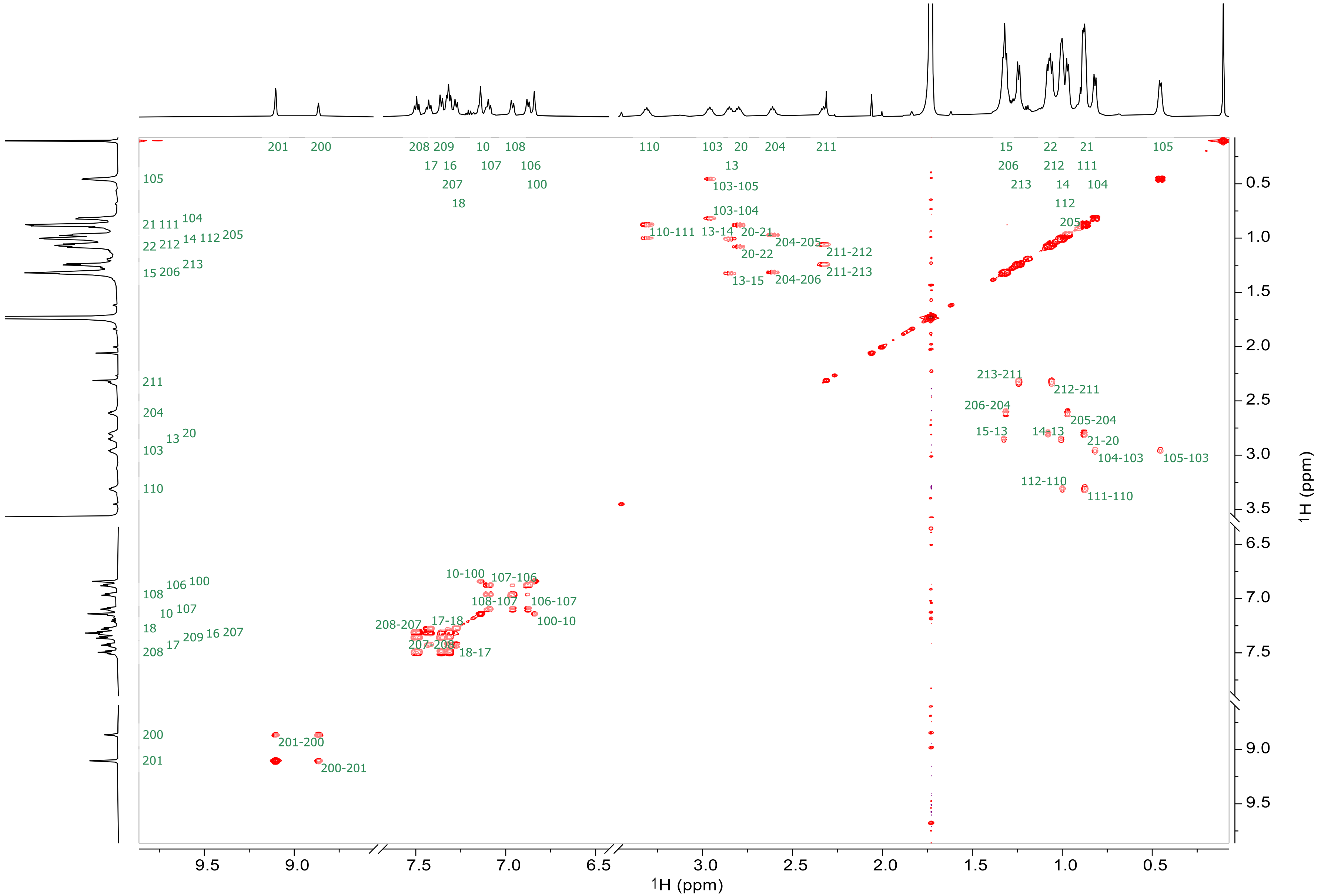
201
200
208 209
17 16
207
18
10
107
108
106
100
110
103
20
13
204
211
15
206
213
22
212
14
112
205
21
111
104
105
103-105
103-104
110-111
13-14
20-21
20-22
204-205
211-212
211-213
13-15
204-206
213-211
212-211
206-204
205-204
15-13
14-13
21-20
104-103
105-103
112-110
111-110
10-100
107-106
108-107
106-107
100-10
208-207
17-18
207-208
18-17
201-200
200-201
9.5
9.0
7.5
7.0
6.5
3.0
2.5
2.0
1.5
1.0
0.5
3.5
1H (ppm)

^{1}H NMR of Complex 9, 400 MHz, CD_2Cl_2, 23 °C

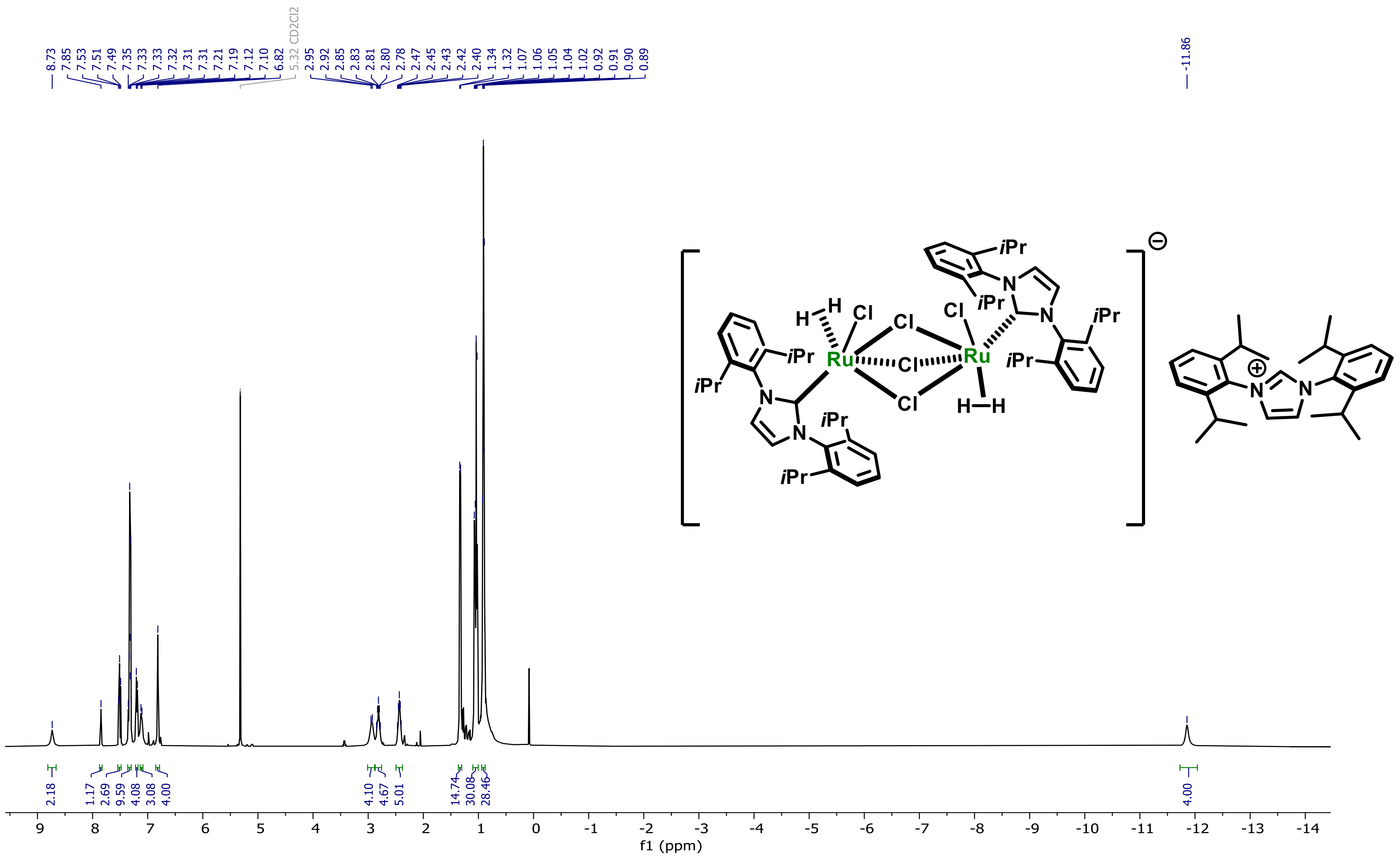

^{13}C NMR of Complex 9, 101 MHz, CD_2Cl_2, 23 °C

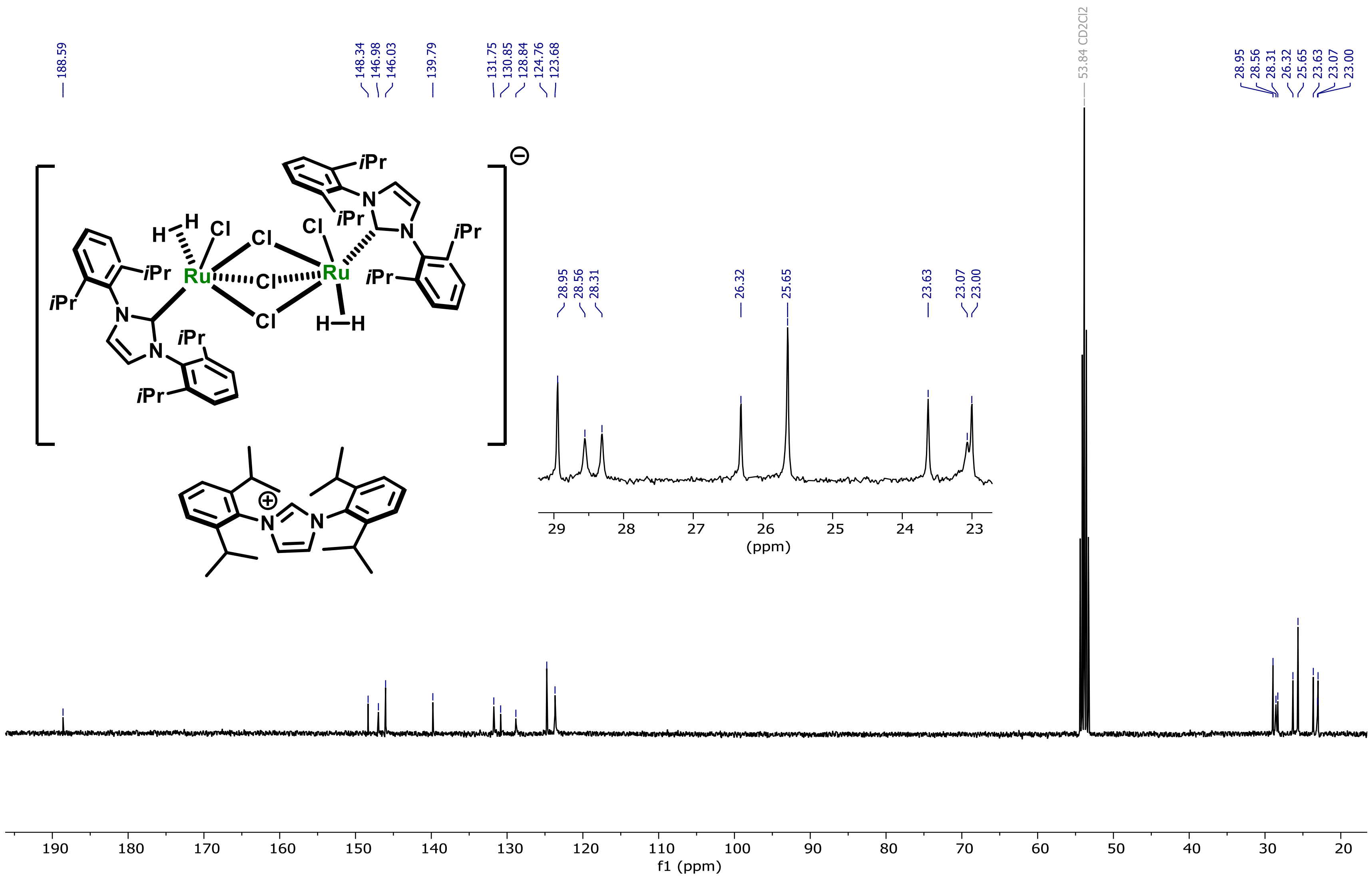

^{1}**H NMR of Complex 9**, 400 MHz, toluene-[D_8], 23 °C

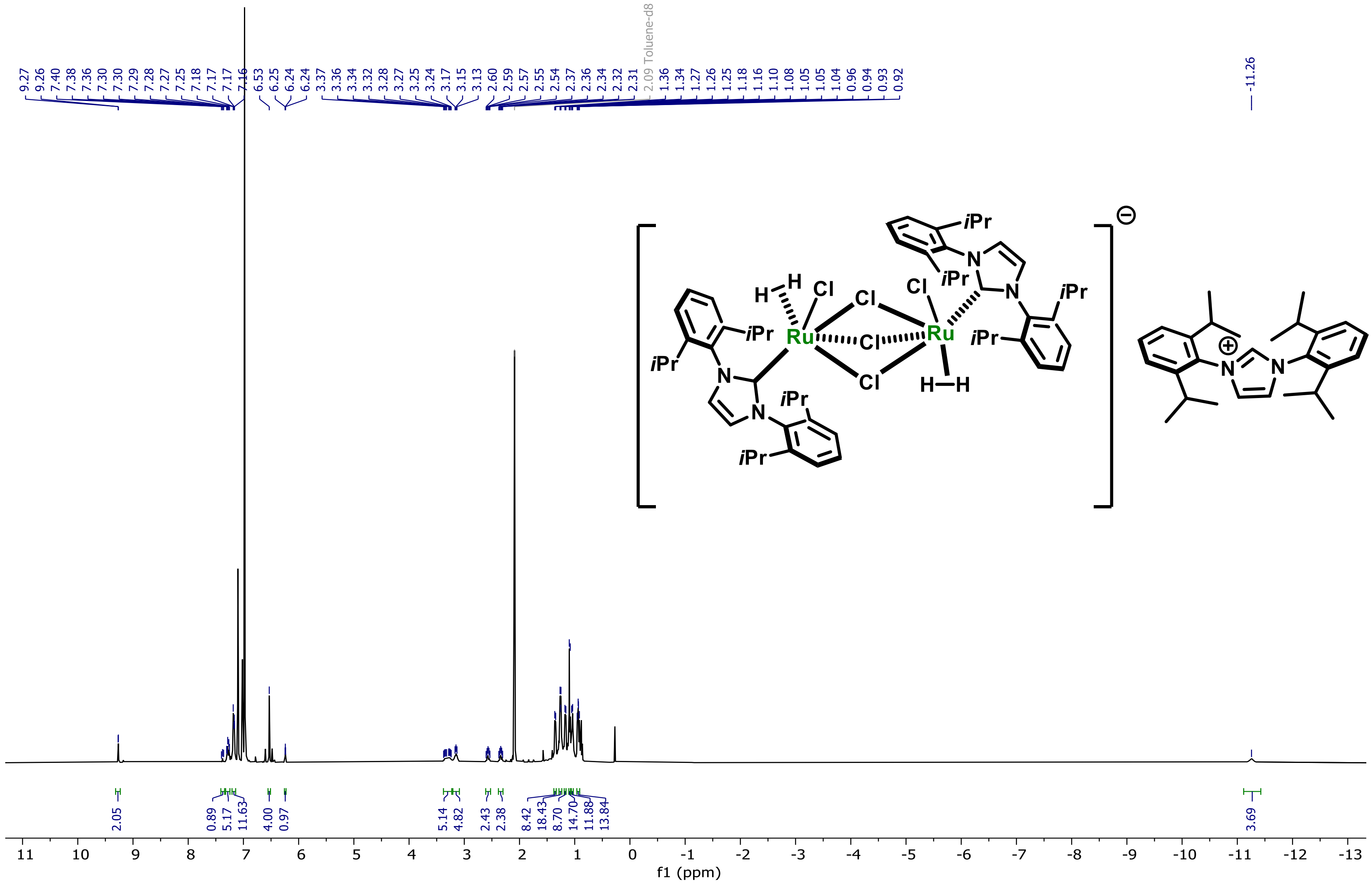

Stacked ^{1}H NMR spectra of Complex 9, top (toluene-[D$_8$]), bottom (CD_2Cl_2)

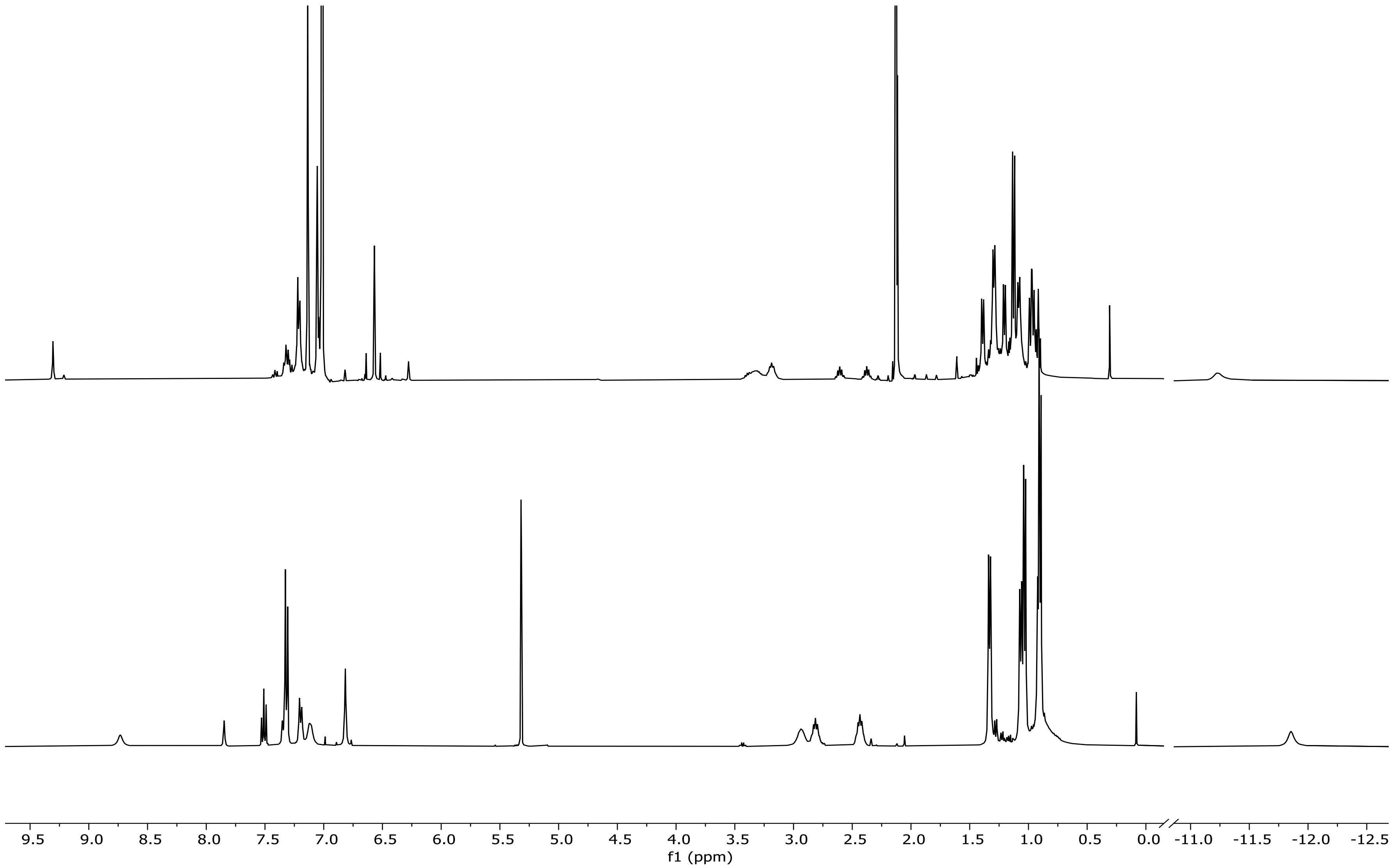

^{1}H NMR of Labelled Complex [D_2]-9, 400 MHz, CD_2Cl_2, 23 °C

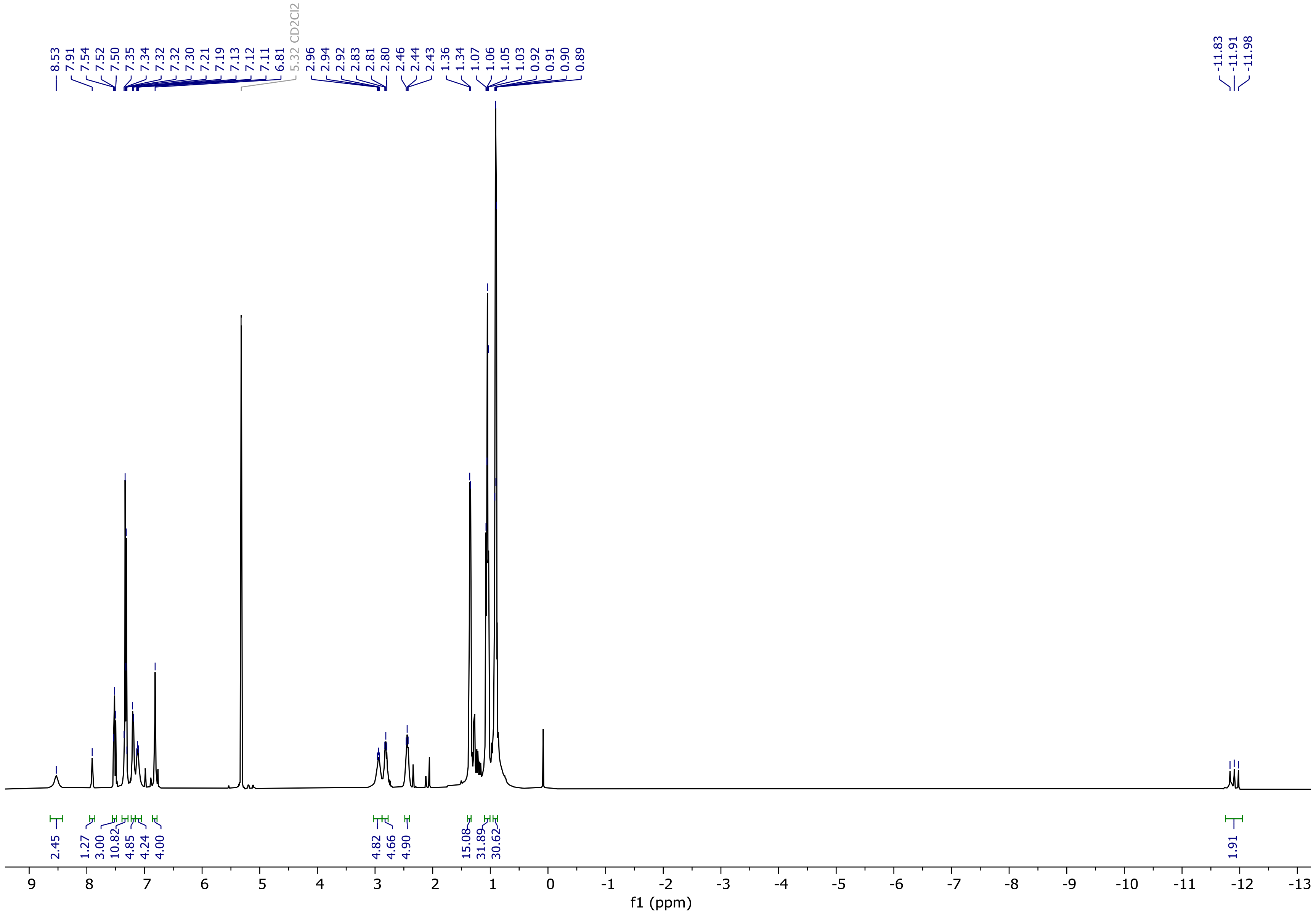

^{13}C NMR of Labelled Complex [D_2]-9, 101 MHz, CD_2Cl_2, 23 °C

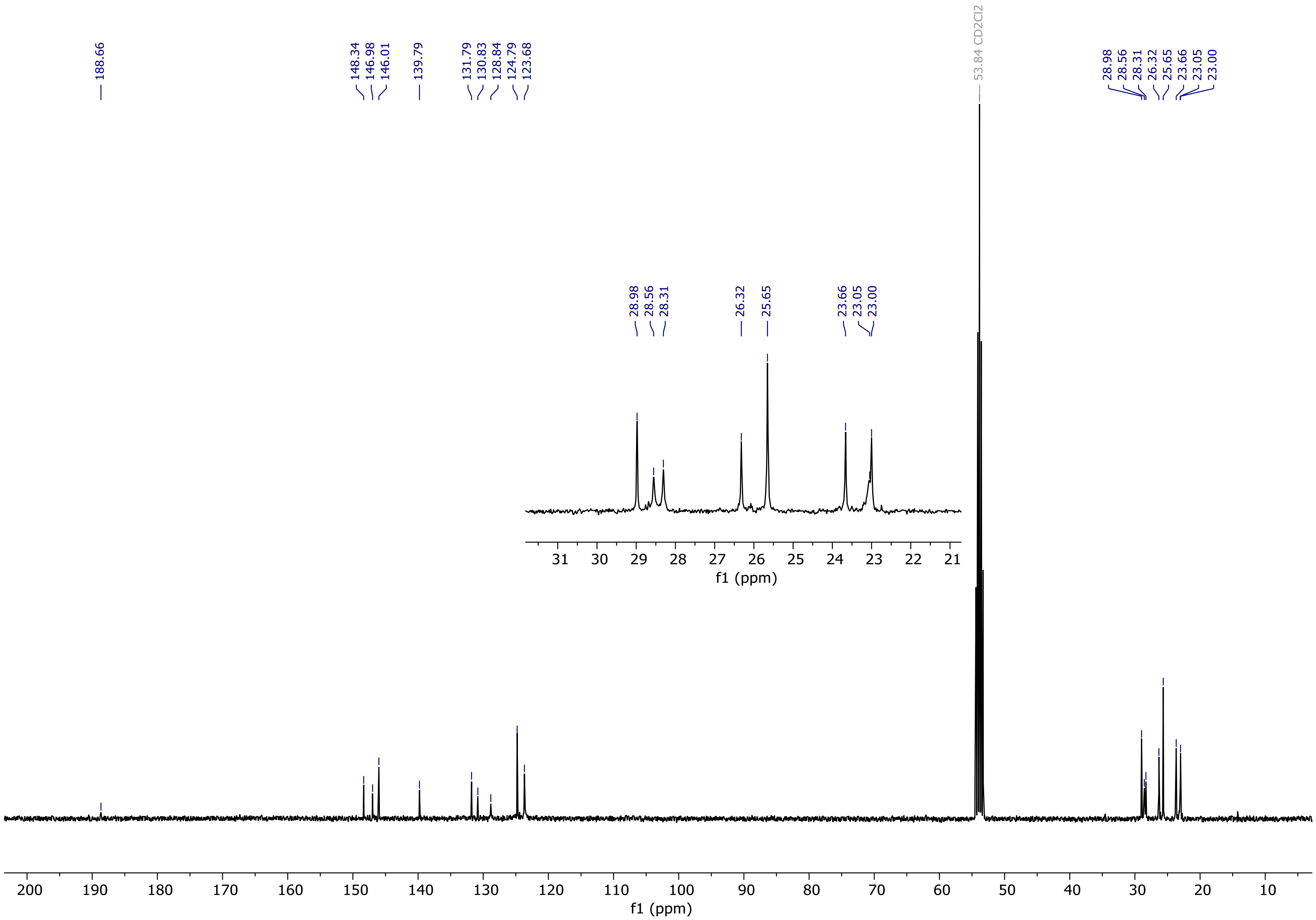

^{1}H NMR of Complex 8, 600 MHz, Toluene-[D_8], 23 °C

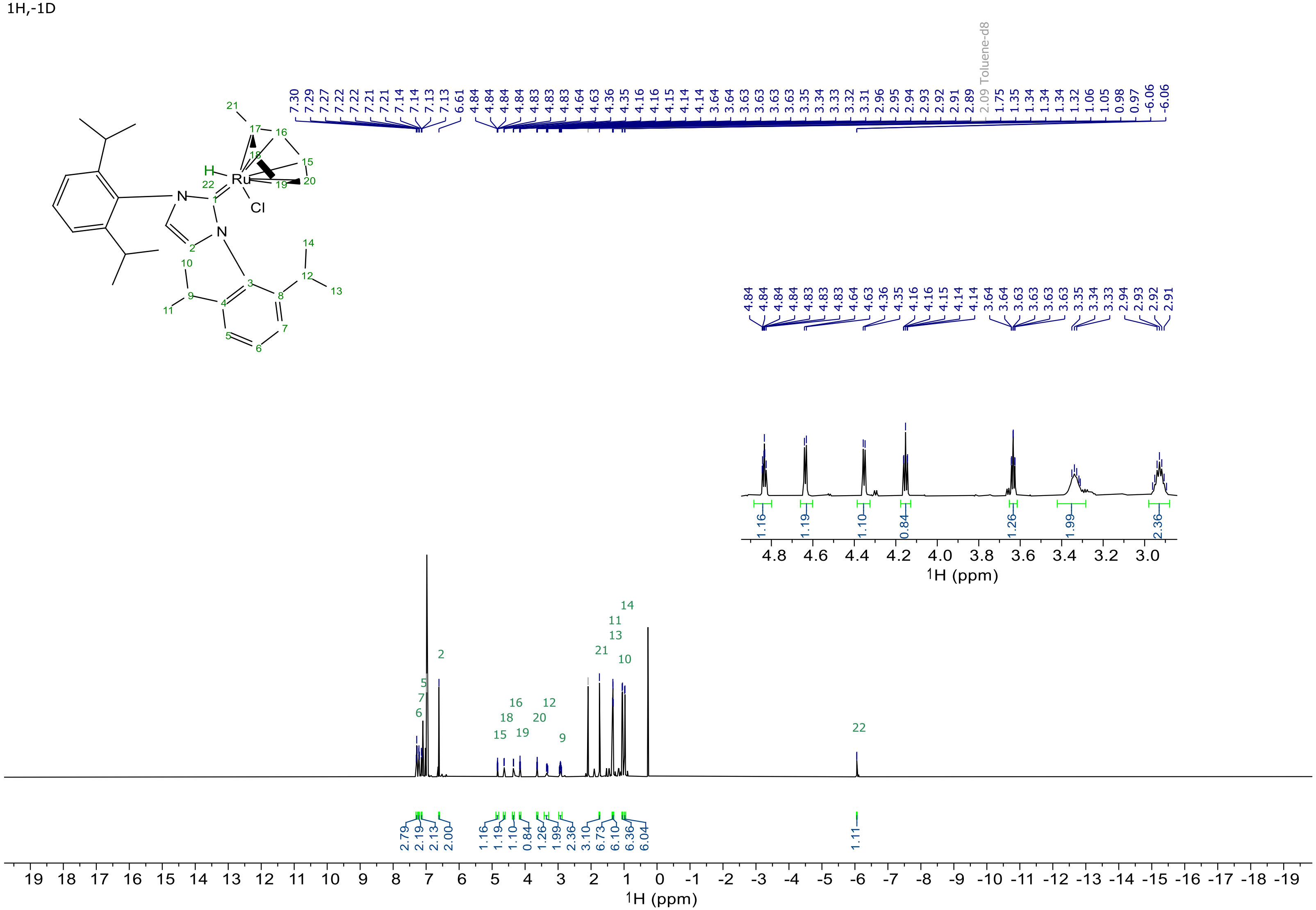

^{13}C NMR of Complex 8, 151 MHz, Toluene-[D_8], 23 °C

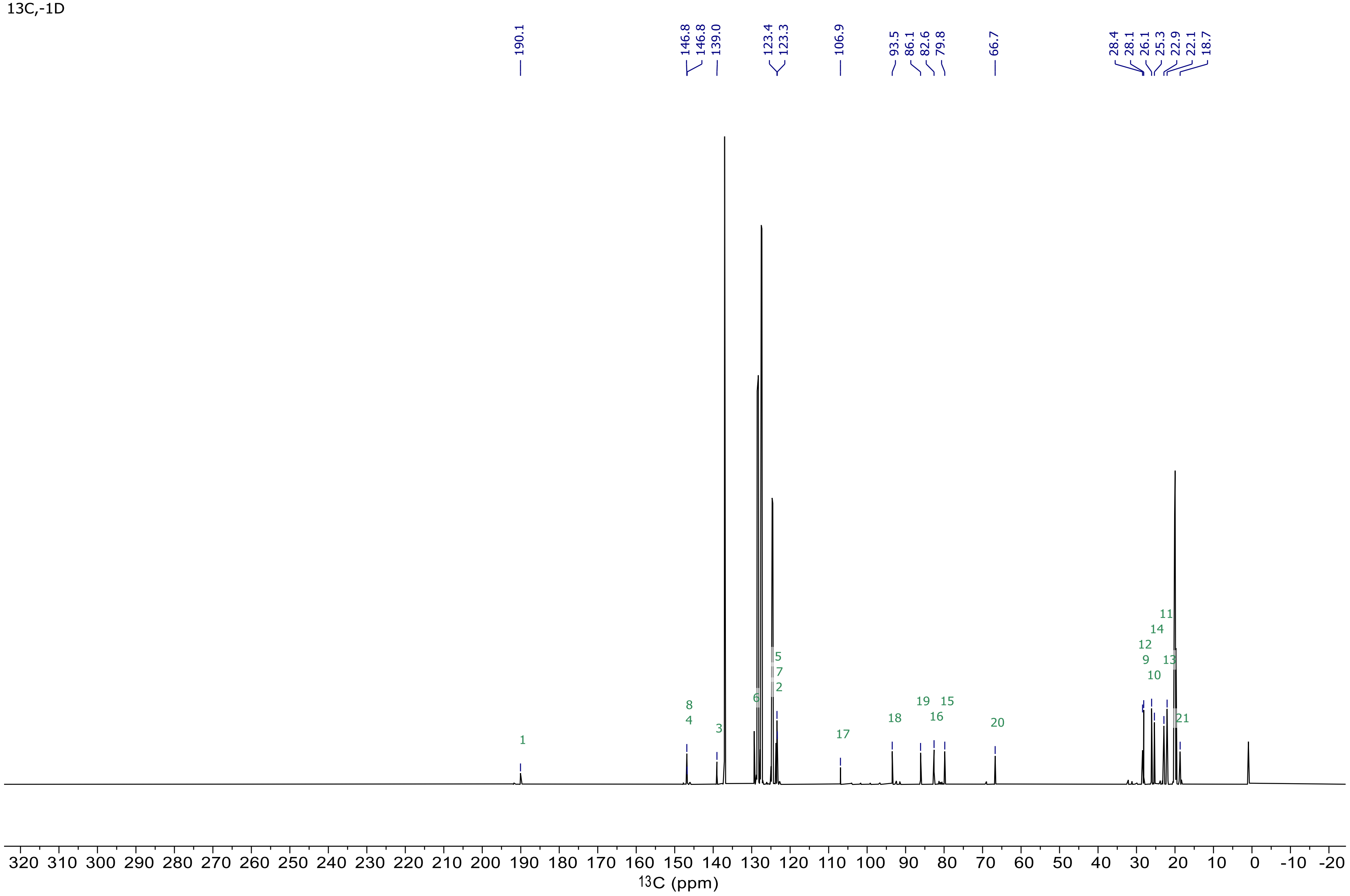

1H,13C-HSQC-EDITED

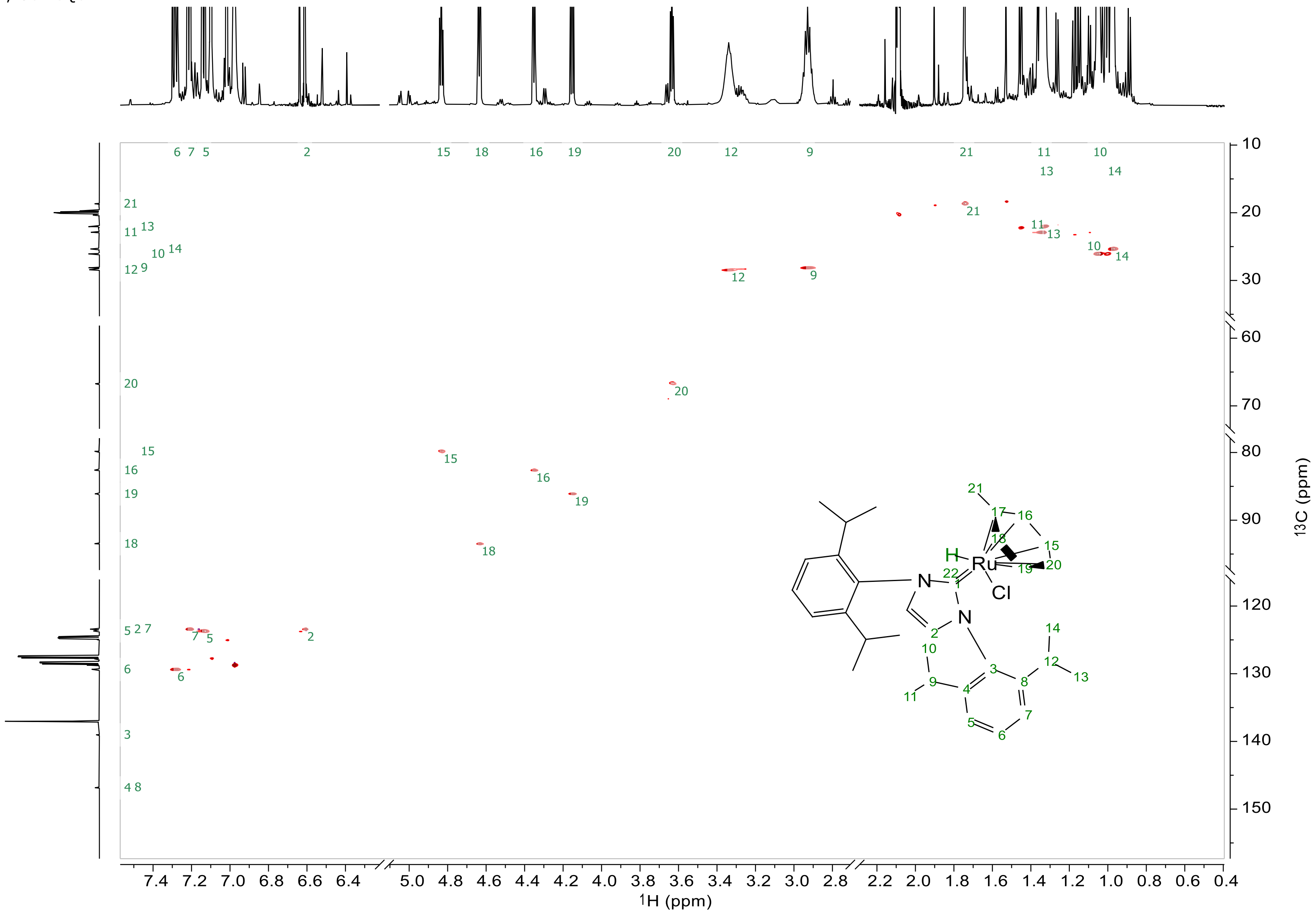

1H,13C-HMBC

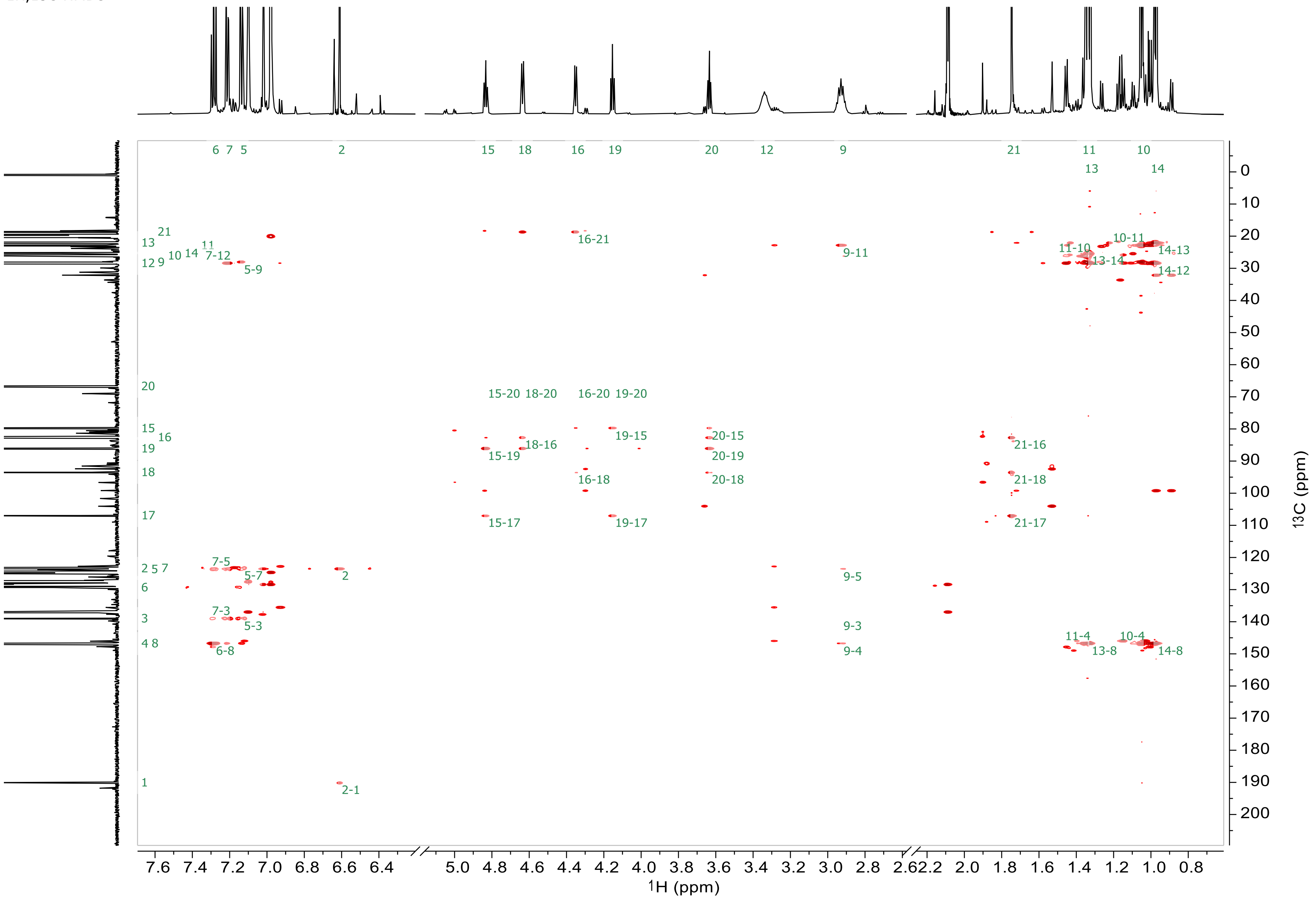

6 7 5
2
15
18
16
19
20
12
9
21
11
13
10
14
13
21
12 9 10 14 11 7-12
5-9
16-21
9-11
10-11
11-10
14-13
13-14
14-12
20
15-20 18-20
16-20 19-20
15
16
19
18
17
20-15
19-15
18-16
21-16
15-19
20-19
16-18
20-18
21-18
15-17
19-17
21-17
2 5 7
6
3
4 8
7-5
5-7
2
9-5
7-3
5-3
9-3
6-8
9-4
11-4
10-4
13-8
14-8
1
2-1
7.6 7.4 7.2 7.0 6.8 6.6 6.4
5.0 4.8 4.6 4.4 4.2 4.0 3.8 3.6 3.4 3.2 3.0 2.8 2.6
2.2 2.0 1.8 1.6 1.4 1.2 1.0 0.8
1H (ppm)
0 10 20 30 40 50 60 70 80 90 100 110 120 130 140 150 160 170 180 190 200
13C (ppm)

1H,13C-HMBC

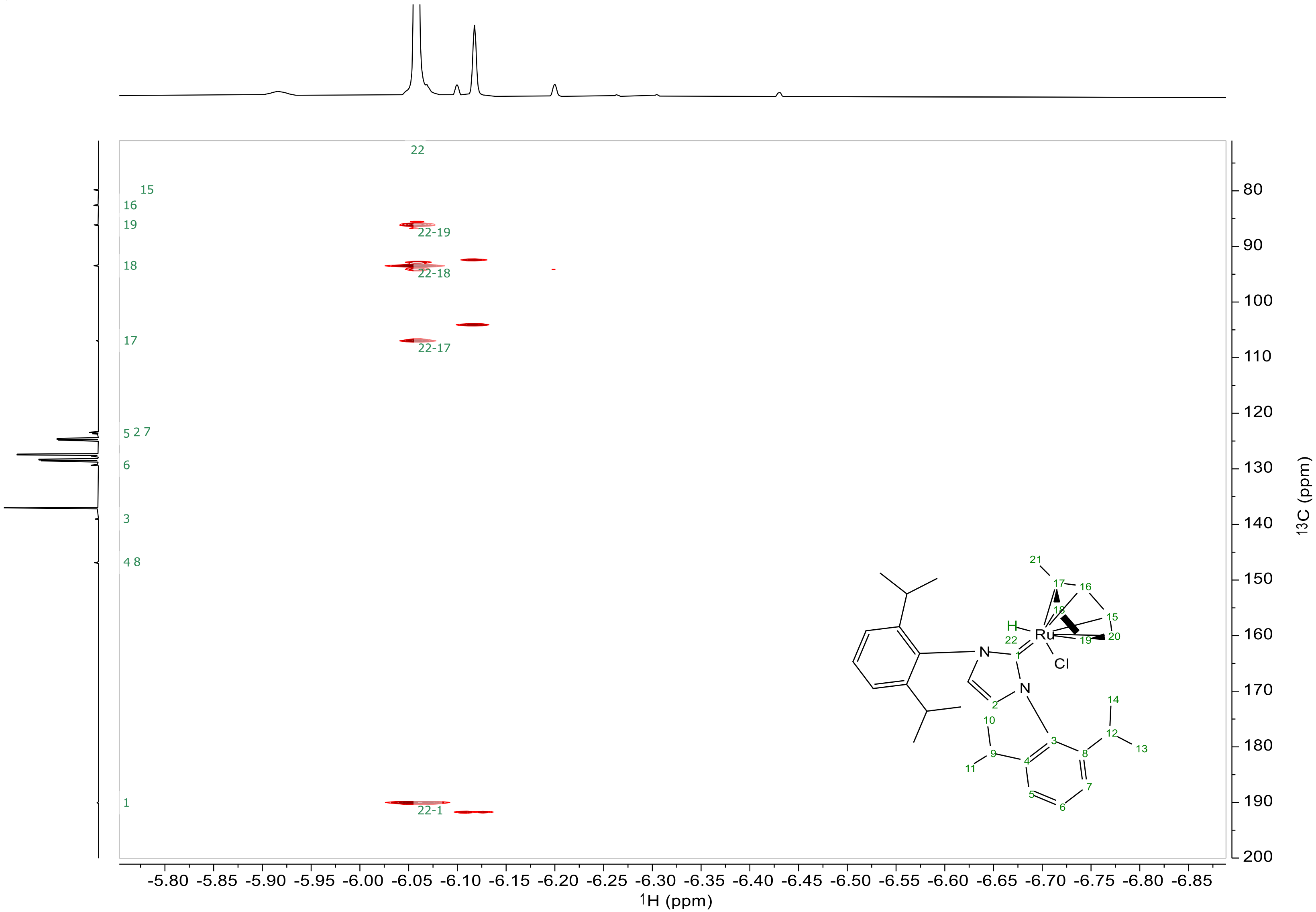

22
15
16
19
22-19
18
22-18
17
22-17
5 2 7
6
3
4 8
1
22-1
80
90
100
110
120
130
140
150
160
170
180
190
200
13C (ppm)
-5.80 -5.85 -5.90 -5.95 -6.00 -6.05 -6.10 -6.15 -6.20 -6.25 -6.30 -6.35 -6.40 -6.45 -6.50 -6.55 -6.60 -6.65 -6.70 -6.75 -6.80 -6.85
1H (ppm)
H
Ru
Cl
N
N

1H,1H-2D-COSY

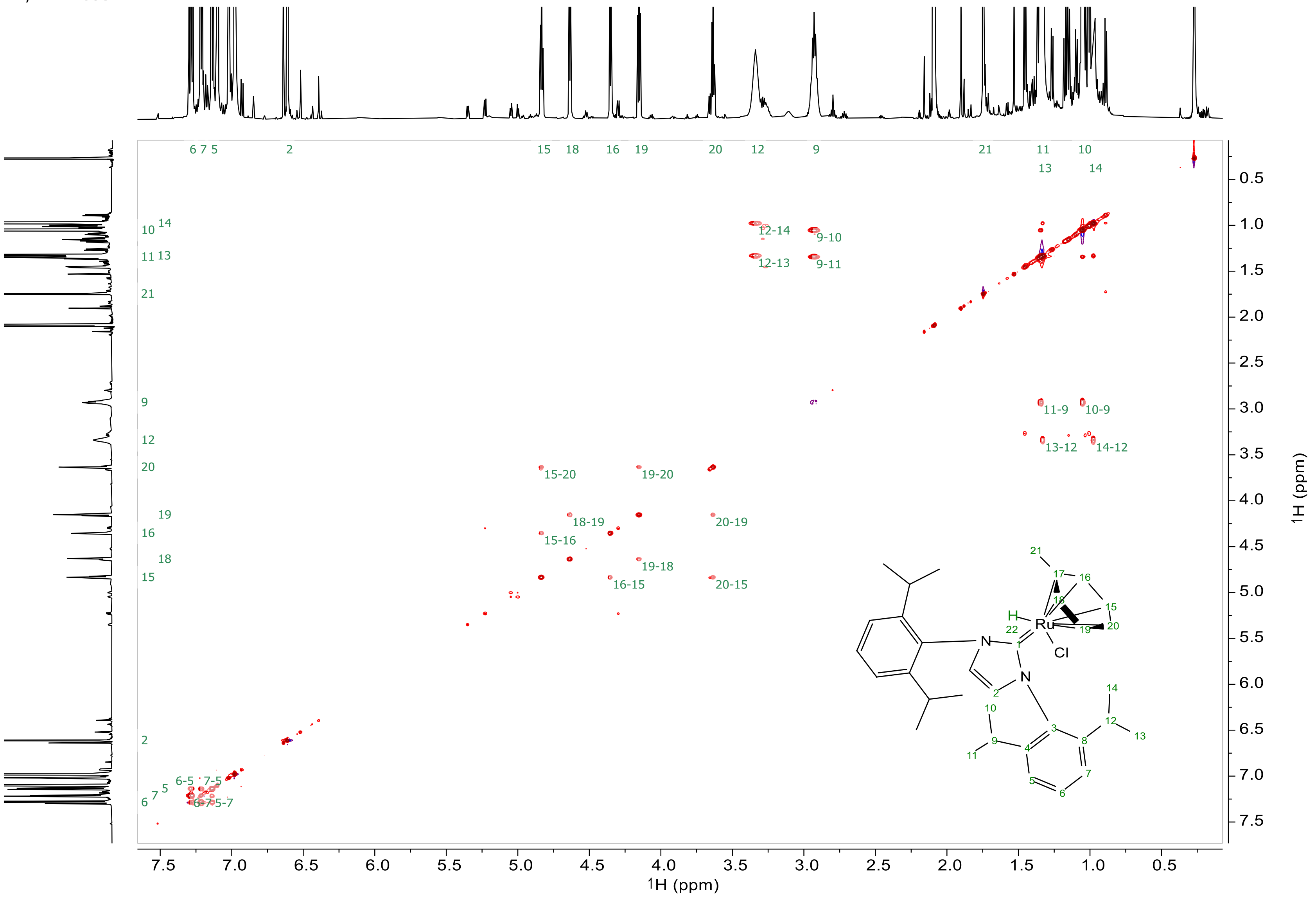

T1 inversion reconovery experiment

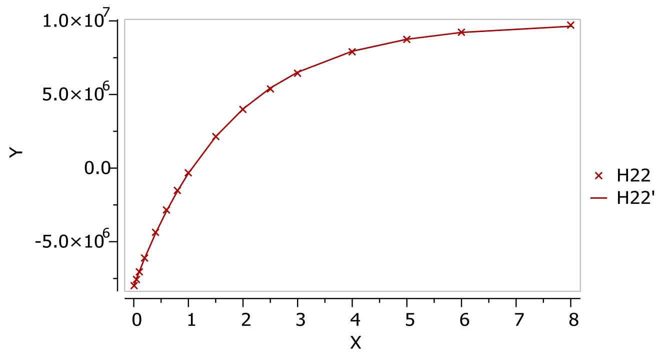

H22' = I0+P*exp(-X/T1)

I0=9.836e+6; P=-1.785e+7; T1=1.784;

Error: 1.818e+5

The relaxation time of H21 is 1.78s

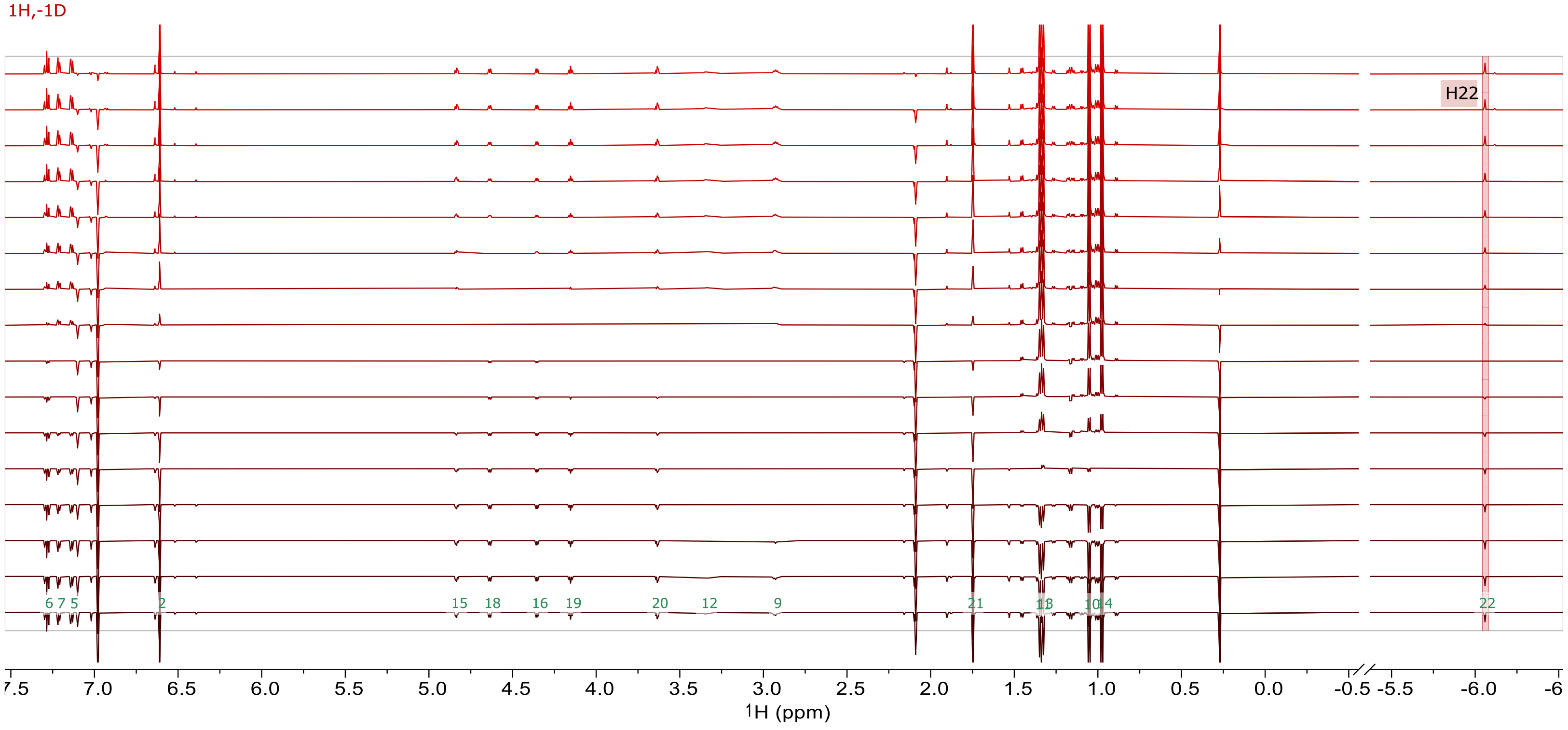

1H,1H-NOESY

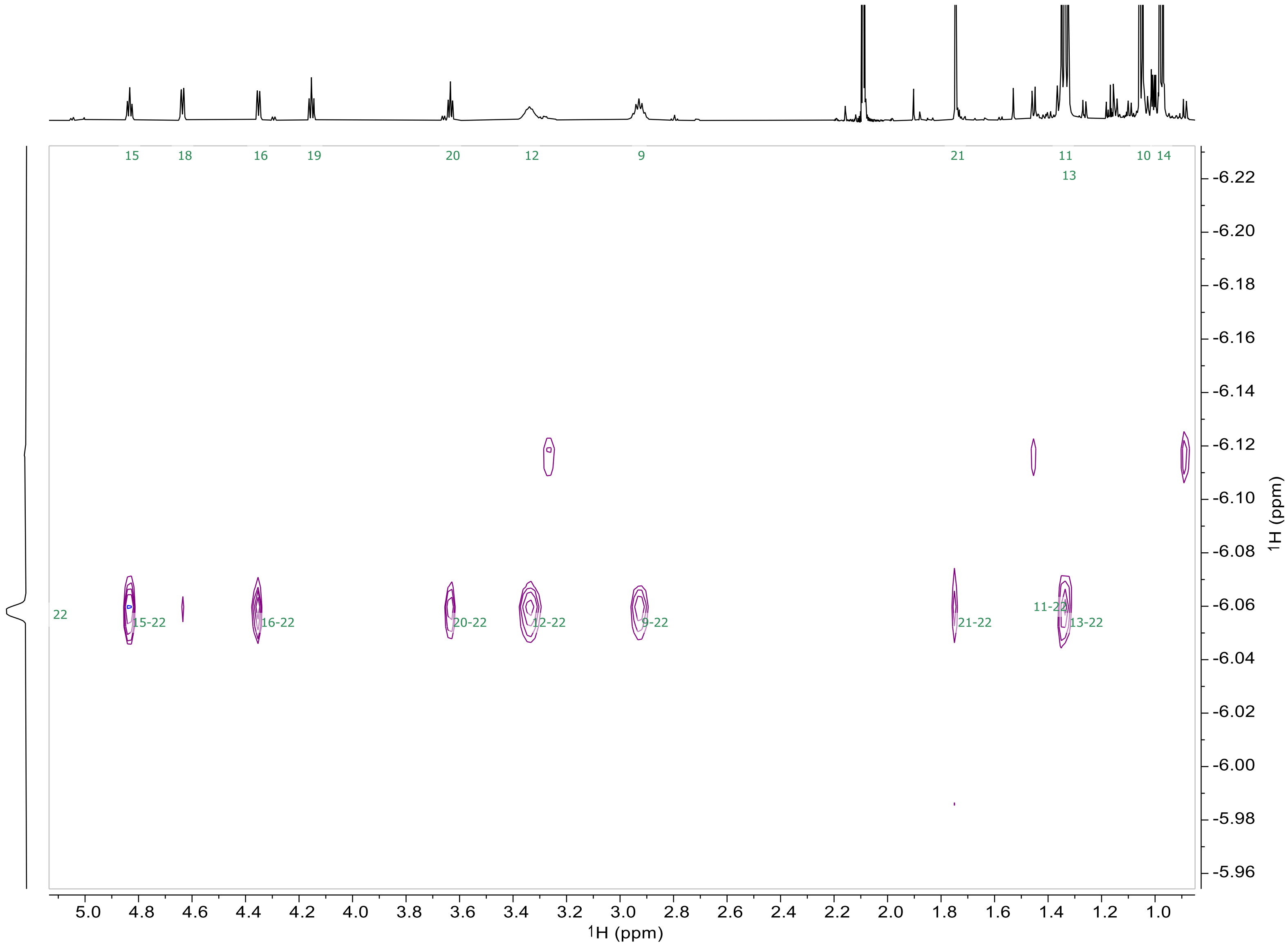

15
18
16
19
20
12
9
21
11
13
10 14
22
15-22
16-22
20-22
12-22
9-22
21-22
11-22
13-22
1H (ppm)
1H (ppm)

1H NMR spectra at different temperatures

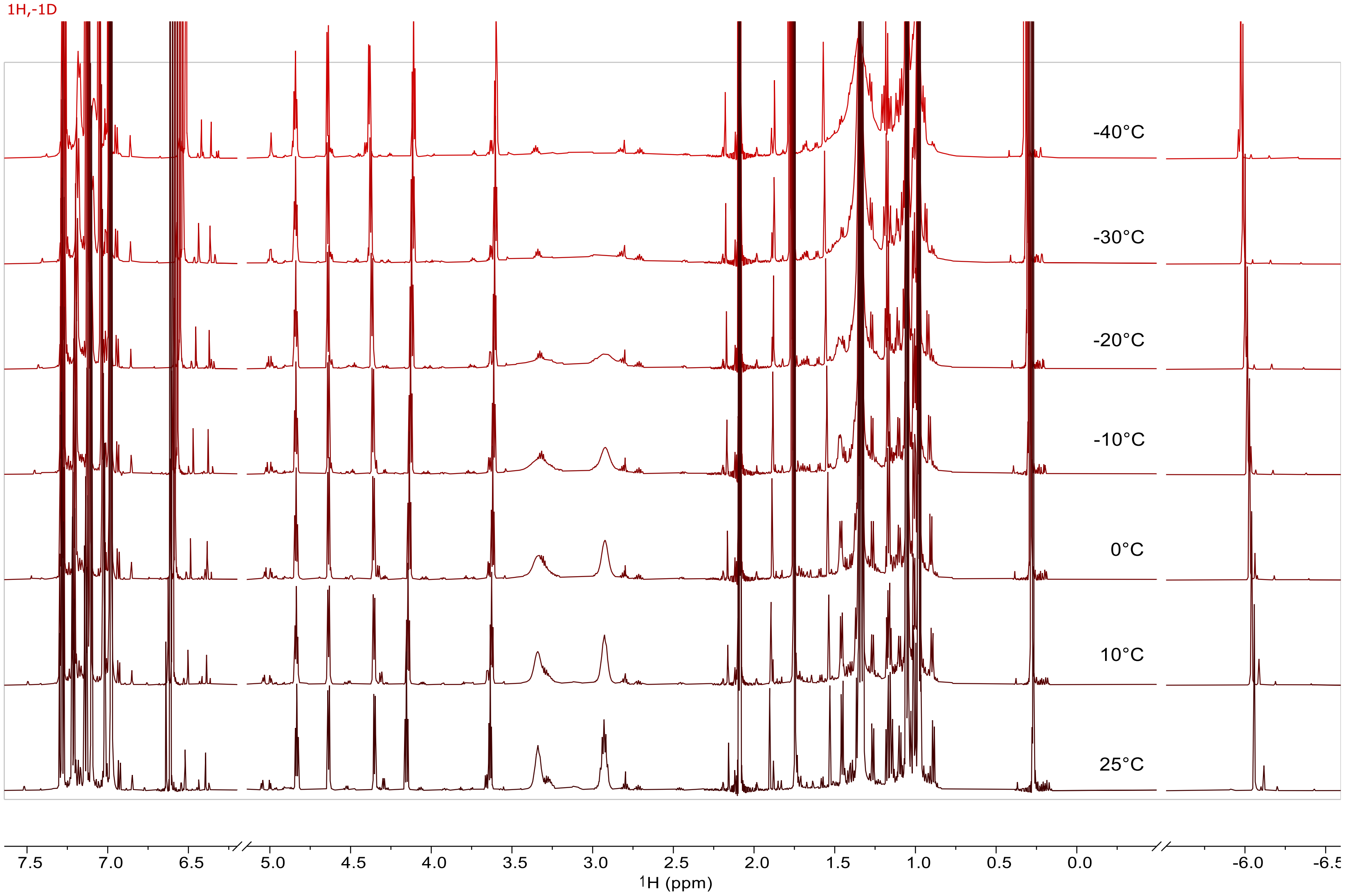

References

[1] T. Biberger, R. J. Zachmann, A. Fürstner, *Angew. Chem. Int. Ed.* **2020**, *59*, 18423-18429.

[2] P. A. Maltby, M. Schlaf, M. Steinbeck, A. J. Lough, R. H. Morris, W. T. Klooster, T. F. Koetzle, R. C. Srivastava, *J. Am. Chem. Soc.* **1996**, *118*, 5396-5407.

[3] T. A. Luther, D. M. Heinekey, *Inorg. Chem.* **1998**, *37*, 127-132.

[4] R. E. Hoffman, *Magn. Reson. Chem.* **2006**, *44*, 606-616.

[5] R. E. Hoffman, E. D. Becker, *J. Magn. Res.* **2005**, *176*, 87-98.

[6] R. H. Morris, *Coord. Chem. Rev.* **2008**, *252*, 2381-2394.

[7] K. W. Zilm, R. A. Merrill, M. W. Kummer, G. J. Kubas, *J. Am. Chem. Soc.* **1986**, *108*, 7837-7839.

[8] C. Adamo, V. Barone, *J. Chem. Phys.* **1999**, *110*, 6158-6170.

[9] M. Dolg, U. Wedig, H. Stoll, H. Preuss, *J. Chem. Phys.* **1987**, *86*, 866-872.

[10] D. Andrae, U. Haeussermann, M. Dolg, H. Stoll, H. Preuss, *Theor. Chim. Acta* **1990**, *77*, 123-141.

[11] J. M. L. Martin, A. Sundermann, *J. Chem. Phys.* **2001**, *114*, 3408-3420.

[12] F. Weigend, R. Ahlrichs, *Phys. Chem. Chem. Phys.* **2005**, *7*, 3297-3305.

[13] G. te Velde, F. M. Bickelhaupt, E. J. Baerends, C. Fonseca Guerra, S. J. A. van Gisbergen, J. G. Snijders, T. Ziegler, *Comp. Chem.* **2001**, *22*, 931. Amsterdam Density Functional (ADF) Theoretical Chemistry Vrieje Universitet see http://www.scm.com (Version 2014).
